# Kinetic Redox Shotgun Proteomics Reveals Specific Lipopolysaccharide Effects on Intestinal Epithelial Cells, Mitigated by a Mn Superoxide Dismutase Mimic

**DOI:** 10.1002/anie.202422644

**Published:** 2025-03-22

**Authors:** Martha Zoumpoulaki, Giovanni Chiappetta, Jean Bouvet, Namita‐Raju John, Gabrielle Schanne, Pauline Gehan, Samuel Diebolt, Shakir Shakir, Elodie Quévrain, Emilie Mathieu, Sylvie Demignot, Philippe Seksik, Nicolas Delsuc, Joelle Vinh, Clotilde Policar

**Affiliations:** ^1^ Laboratoire Chimie Physique et Chimie du Vivant—CPCV UMR8228 Département de Chimie, Ecole Normale Supérieure PSL University, Sorbonne Université, CNRS Paris 75005 France; ^2^ Laboratoire de Spectrométrie de Masse Biologique et Protéomique SMBP UAR2051 ESPCI Paris PSL University, CNRS Paris 75005 France; ^3^ Gastroenterology Department Sorbonne Université INSERM Centre de Recherche Saint‐Antoine CRSA Paris Center for Microbiome Medicine (PaCeMM) FHU AP‐HP, Saint‐Antoine Hospital Paris France; ^4^ EPHE PSL University Paris 75014 France

**Keywords:** Bioinorganic chemistry, Lipopolysaccharide (LPS), Oxidative stress, Quantitative redox proteomics OcSILAC, Superoxide dismutase (SOD) mimic or mimetic

## Abstract

Overproduction of reactive oxygen species and antioxidant superoxide dismutases (SOD1, SOD2) dysregulation contribute to chronic inflammation such as generated in inflammatory bowel diseases (IBD). A kinetic redox shotgun proteomic strategy (OcSILAC for Oxidized cysteine Stable Isotope Labelling by Amino acids in Cell culture) was used to explore the lipopolysaccharide (LPS) effects including LPS‐induced oxidation and inflammation cascades on a dedicated intestinal epithelial cell line (HT29‐MD2) together with the potential mitigating role of a Mn‐based SOD‐mimic **Mn1**. While LPS induced transient oxidative damages at early times (15 min), cells incubated with **Mn1** showed, in this time frame, a significantly reduced cysteine oxidation, highlighting **Mn1** antioxidant properties. Over time, cysteine oxidation of LPS‐treated cells was counteracted by an overexpression of antioxidant proteins (SOD1, NQO1) and a late (6 h) preponderant increase in SOD2 level. **Mn1**, when co‐incubated with LPS, attenuated the level of most LPS‐modified proteins, that is, proteins involved in the inflammatory response. Our results highlight **Mn1** as a potentially effective antioxidant and anti‐inflammatory agent to consider in the treatment of IBD, as well as a useful tool for exploring the interconnection between oxidative stress and inflammation.

## Graphical abstract

Proteomics and redoxomics are efficient to quantify the effects of bacterial lipopolysaccharide in intestinal epithelial cells (HT29) at the proteome and at the Cys‐redoxome levels. Oxidative stress is the initial trigger of LPS leading to inflammation. A Mn‐based superoxide mimetic (or mimic) was able to reduce oxidative stress and to mitigate the effects at the proteome level.

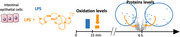

## Introduction

The increased understanding that redox imbalance in biology is a key etiological and triggering factor in many disabling processes and diseases has led to exploring antioxidants as potential drugs.^[^ ^1^ ^]^ The redox homeostasis is disrupted under chronic immunoreceptor activation^[^ ^2^ ^]^ leading to chronic inflammatory pathologies like inflammatory bowel diseases (IBD).^[^ ^3^ ^]^ Increased levels of reactive oxygen species (ROS) were detected in vitro in HT29 intestinal cells after chronic inflammatory or microbial stimuli,^[^ ^4^ ^]^ ex vivo in cultured human IBD biopsies,^[^ ^5^ ^]^ and in vivo in the inflamed intestinal mucosa of IBD patients.^[^ ^6^ ^]^ In addition, anti‐superoxide enzymes were shown to be dysregulated in IBD‐inflamed mucosal epithelium: the cytosolic Cu/Zn superoxide dismutase (SOD1) was under‐expressed, and the mitochondrial MnSOD (SOD2) was overexpressed but in an enzymatically inactive form.^[^ ^7^ ^]^ Increased ROS were shown to cause the oxidation of membrane lipids,^[^ ^8^ ^]^ cytoskeletal proteins,^[^ ^9^ ^]^ and deoxyribonucleic acid (DNA),^[^ ^10^ ^]^ in intestinal inflamed mucosa and plasma of IBD patients.^[^ ^11^ ^]^ Moreover, ROS excess activates redox‐sensitive transcription factors like the nuclear factor‐kappa B (NF‐kB), and the nuclear factor erythroid 2‐related factor 2 (NRF2), regulating several immune and inflammatory genes.^[^ ^12^ ^]^ Although current treatments of IBD exclusively target inflammatory response, new antioxidant therapeutic options are under development as the significance of oxidative stress in IBD becomes clearer.^[^ ^13^ ^]^

**Figure 1:**
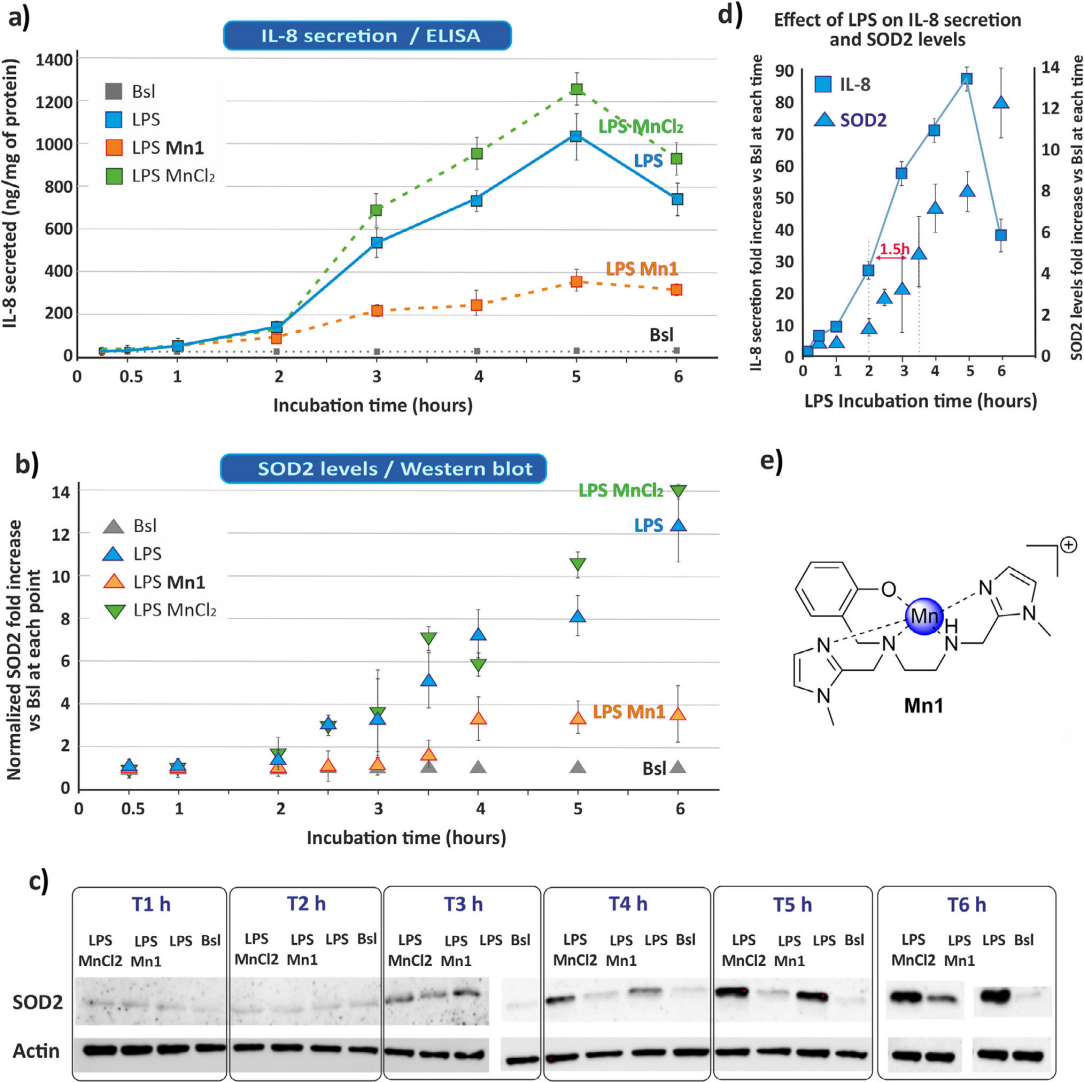
Kinetic study of IL‐8 secretion and SOD2 levels. HT29‐MD2 cells were incubated with cell culture medium (basal conditions, Bsl) or with LPS (0.1 µg/mL), LPS with **Mn1** (100 µM) or with MnCl_2_ (100 µM) [labeled LPS, LPS **Mn1** and LPS MnCl_2_, respectively] at different time points. a) IL‐8 secretion was measured in the culture medium by ELISA. The IL‐8 amount was normalized by the protein amount in each cell culture and is expressed as ng/mg of protein. b) SOD2 levels in cell lysates as assessed by WB. Actin or total proteins were used as loading controls for data normalization (representative blots shown in Figure 1c, full blots in S.3.1A and B). Results are expressed as a fold increase versus the basal condition at each time point. c) Representative WB of SOD2 (full blots in Figure S.3.1A). Actin was used for data normalization. d) Effect of LPS on IL‐8 secretion and SOD2 levels over time. Data are expressed as a fold increase versus basal value, for each time point. Independent experiments were performed at least three times and the values are given as means ± SD. e) Chemical structure of the SOD mimic [Mn(II)(enPI_2_)]^+^, labeled **Mn1**.

Low‐molecular‐weight Mn complexes reproducing the catalytic SOD activity, called SOD mimics or mimetics, can be used as catalytic anti‐superoxide agents.^[^ ^14^, ^15^, ^16^, ^17^, ^18^ ^]^ They catalyze the dismutation of superoxide (O_2_^—^), a ROS produced by the monoelectronic reduction of dioxygen (O_2_). The SOD mimic [Mn^II^(enPI_2_)]^+^, labeled **Mn1** hereafter (Figure 1e), is easily synthesized, not cytotoxic, and cell‐permeant.^[^ ^19^, ^20^, ^21^ ^]^ The HT29‐MD2 cell line^[^ ^22^ ^]^ was used as a cellular model of inflammation and oxidative stress to assay SOD mimics in a biologically relevant cellular context.^[^ ^19^, ^20^, ^21^ ^]^ The wild‐type intestinal epithelial cell line HT29 expresses the Toll‐Like Receptor 4 (TLR4), a bacterial lipopolysaccharide (LPS) receptor, but almost no myeloid differentiation protein‐2 (MD‐2), a secreted protein which is required for TLR4 activation in response to lipopolysaccharide (LPS), leading to IL‐8 production.^[^ ^23^ ^]^ IL‐8 secretion by the intestinal epithelial cells is a crucial event in the pathogenesis of IBD as IL‐8 acts as a chemotactic factor for neutrophils, leading to inflammation. IL‐8 is strongly expressed in active IBD as compared to quiescent IBD, and to normal tissue.^[^ ^24^, ^25^ ^]^ In this setting and in order to reproduce a relevant model of activated epithelial cells producing IL‐8 in response to LPS as seen in IBD, a HT29 model responsive to LPS by stably transfecting MD‐2 in this cell line was constructed. This HT29‐MD2 cell line was shown to exhibit an inflammatory response to LPS especially IL‐8 increased over 100‐fold compared to HT29.^[^ ^22^ ^]^ The LPS concentration necessary to induce IL‐8 secretion was within the range observed in vivo.^[^ ^26^ ^]^ Notably, in addition to a strong increase in the secretion of IL‐8, a 6‐h LPS stimulation led to increased levels of cyclooxygenase 2 (COX2) and SOD2.^[^ ^19^, ^20^, ^21^ ^]^ This is in line with the fact that an LPS challenge induces ROS production,^[^ ^27^ ^]^ SOD2 overexpression being considered as an antioxidant feedback response.^[^ ^19^ ^]^ Interestingly, **Mn1** displayed an anti‐superoxide and anti‐inflammatory activity on LPS‐stimulated HT29‐MD2 cells, whereas MnCl_2_ did not, showing the importance of the complex and not only of the metal cation. Indeed, after a 6‐h coincubation with LPS and **Mn1**, the levels of IL‐8, COX2, and SOD2 were reduced as compared to LPS treatment alone.^[^ ^19^, ^21^ ^]^ Moreover, an oral administration of **Mn1** decreased the weight loss observed in mice with induced colitis.^[^ ^19^ ^]^ In a previous work,^[^ ^21^ ^]^ **Mn1** incubated at 100 µM was found as one of the most active SOD mimics in the HT29‐MD2‐LPS assay within a series including nitroxide (mitoTEMPOL), a Mn‐based cyclopolyamine (M40403), an open polyamine (EUK‐134) and several porphyrins (e.g., MnTnBuOE‐2‐PyP^5+^), even if the mentioned porphyrin and Mn‐based cyclopolyamine showed to be more active than **Mn1** at a lower incubation concentration (10 µM).

To understand the full scope of the LPS effects, notably the link between oxidative stress and inflammation, and the **Mn1** mode of action, an innovative integrated proteomic and redoxomic shotgun (or bottom‐up) strategy^[^ ^28^, ^29^ ^]^ labelled OcSILAC (for Oxidized cysteine Stable Isotope Labeling of Amino acids in Cell culture) was undertaken on HT29‐MD2 cells. It provides an overview of the proteome modifications and the oxidation state evolution of proteome‐wide cysteines, the main target of ROS protein oxidation based on a bottom‐up identification of the proteome and Cys‐proteome redox state by mass spectrometry.^[^ ^30^ ^]^ We focused on reversible Cys oxidations to get information on oxidative stress and its possible reversal. In doing so, we intended first to bridge the gap between inflammation, observed as an end effect, and oxidative stress, which can act as a trigger in this model. We thus studied the effects of LPS on HT29‐MD2 cells at the proteome level and Cys redoxome level in comparison with control cells (not treated with LPS, that is, basal condition). We also aimed to investigate whether the anti‐inflammatory effects observed with **Mn1** could be associated with its antioxidant properties by comparison with LPS‐stimulated cells coincubated with **Mn1**. Interestingly, **Mn1** mitigated the effect of LPS at the proteome level (6‐h incubation). Because the effects observed after a 6‐h treatment were mainly at the proteome level, with no significant cysteine oxidation, we decided to investigate further these experimental conditions at earlier time points. Indeed, as supported by our previous observation that the SOD2 level is increased after a 6‐h treatment,^[^ ^19^ ^]^ the oxidative stress may have already been resolved at this time point. Interestingly, a transient increase in oxidized thiols in the proteome was observed as early as 15 min of incubation with LPS, which was reduced in the presence of **Mn1**.

The importance of this work is underlined by the increasing recognition in the role of redox biology in the context of health and disease. By providing a deeper understanding of how LPS induces oxidative stress associated inflammation, and how a Mn‐based SOD mimic impacts cellular systems under oxidative stress and inflammation, this study contributes to the fundamental knowledge necessary for advancing therapeutic strategies against oxidative stress‐related diseases.

## Results and Discussion

### LPS‐Induced IL‐8 Secretion and SOD2 Levels Increase Are Mitigated by Coincubation with Mn1 over Time

To get insights on the correlation between oxidation and inflammation, we measured the SOD2 levels and IL‐8 secretion in a time‐course study through the comparison of non‐stimulated cells (labeled Bsl for basal condition), LPS‐stimulated (labeled LPS), coincubated with LPS and **Mn1** (labeled LPS Mn1) or MnCl_2_ (labeled LPS MnCl_2_). MnCl_2_ was used as a control to discriminate between the effect of the complex and Mn(II) that could have been released by the ligand (N‐(2‐hydroxybenzyl)‐N,N’‐bis[2‐(N‐methylimidazolyl)methyl]‐ethane‐1,2‐diamine, see Figure 1e for the structure of **Mn1** with its ligand).^[^ ^19^, ^20^, ^21^, ^31^ ^]^ IL‐8 secretion was hardly detected by ELISA (Enzyme‐linked immunosorbent assay) in the basal condition (Figure 1a). Its level strongly increased upon LPS activation with a decrease at 6 h.

SOD2 overexpression was detected by Western blot (WB) 2 h after LPS treatment, that is 1.5 h later than the onset of IL‐8 over‐secretion and increased up to 6 h (Figure 1). The SOD2 increase may be the feedback response to earlier ROS production to resolve oxidative stress, as previously suggested.^[^ ^19^ ^]^ As shown in Figure 1, coincubation of LPS with **Mn1** mitigated LPS effects, with a limitation of both IL‐8 secretion and SOD2 expression. This reinforces the idea that oxidative stress precedes LPS‐induced inflammation, and explains how antioxidants can act as anti‐inflammatory agents.^[^ ^32^, ^33^ ^]^ In LPS MnCl_2,_ the IL‐8 increased secretion and SOD2 overexpression showed no significant change compared to LPS alone, in line with the specificity and relevance of the **Mn1** complex over the Mn(II) ion.^[^ ^19^, ^20^, ^21^ ^]^

### An OcSILAC Experiment was Designed to Detect Both Proteome and Redoxome Modifications after LPS Stimulation, Both with and Without Mn1

After the targeted analysis of IL‐8 and SOD2, a SILAC bottom‐up proteomics strategy was performed to study the effects of LPS and LPS **Mn1** on the whole HT29‐MD2 proteome after a 6‐h treatment. Light and heavy cells were grown, respectively in the classic medium (light cells, treated with different conditions), and in a lysine and arginine heavy isotope enriched medium (heavy cells, untreated basal cells) and then pooled in equal number before the cysteine redox proteomic workflow. Quantifications of both proteins and Cys‐oxidation were performed at the peptide level by mass spectrometry (MS) calculating the area under the curve of the extracted ion chromatogram of the heavy (basal, untreated or T0 for time zero) and light (treated) peptide forms. We have validated the SILAC metabolic studies on HT29‐MD2 cells, with preservation of the cell viability and no detectable bias between the heavy and light conditions (Supporting Information 1 A; Figures S.1.1 and S.1.2). Surprisingly, while fold‐changes of LPS‐modified proteins were up to 2.5, most of the mitochondrial proteins, including SOD2 previously shown to be overexpressed,^[^ ^19^ ^]^ were not identified (Table S.1.1). To improve the detection of the mitochondrial proteins, we implemented the proteomic protocol with a subcellular fractionation step (Supporting Information 1 B, Figure S.1.3). SOD2, notably, was then detected. The two most abundant subcellular fractions were used in the rest of the study, namely cytosolic and plasma membrane & organelles (PMO) fractions, providing enrichment of mitochondrial (inner/outer membrane, matrix proteins, electron transport chain (ETC) components, ribosomal subunits), endoplasmic reticulum (ER) and Golgi proteins (Figure S.1.4).

**Figure 2:**
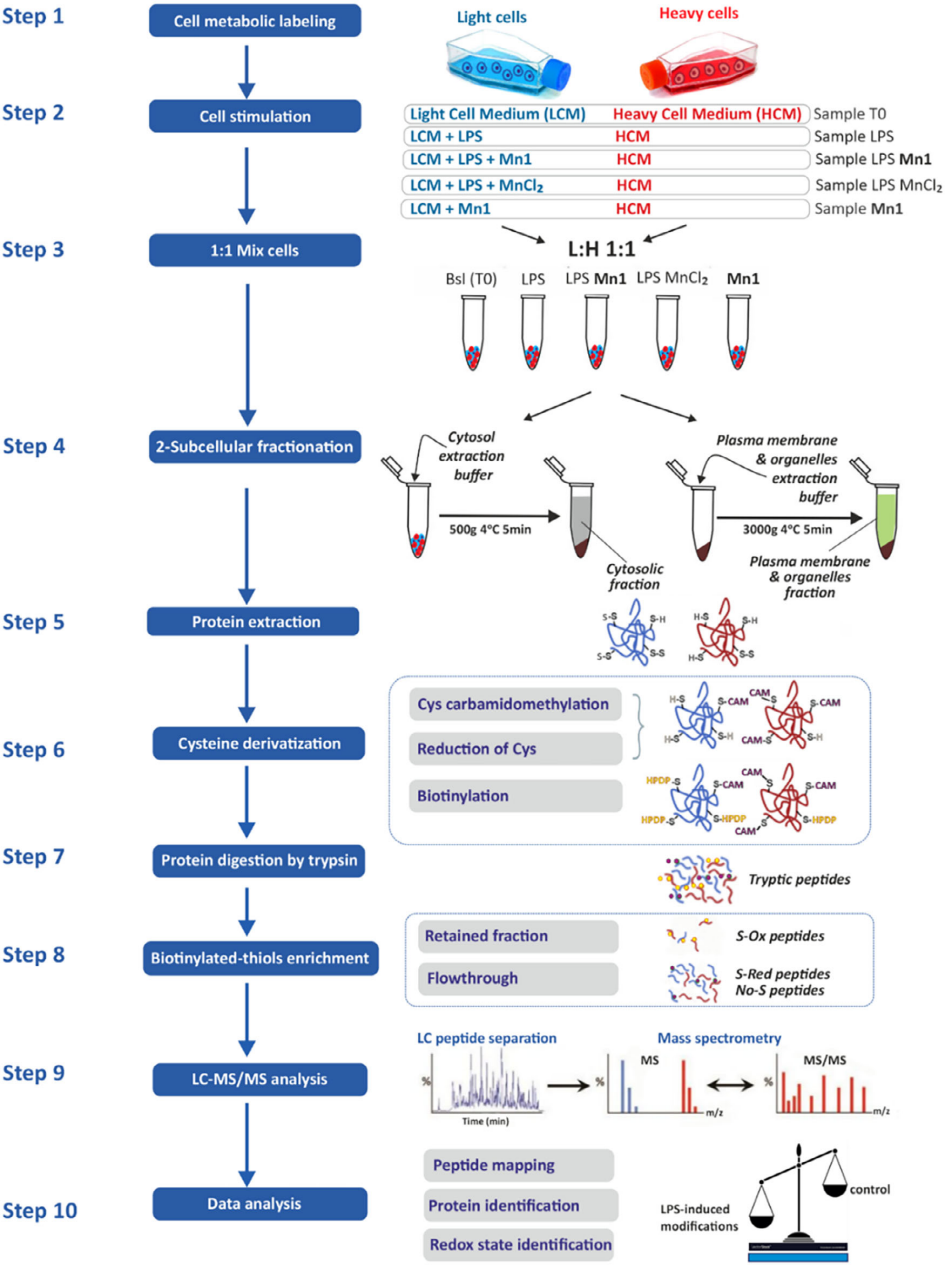
Workflow of OcSILAC combined with subcellular fractionation in HT29‐MD2 cells. Step 1: Metabolic labeling. Step 2: Cell stimulation; Light cells were treated with different conditions (cell culture medium time zero or T0, LPS, LPS **Mn1**, LPS MnCl_2_, **Mn1**) in blue, and Heavy untreated cells (cell culture medium only) shown in red. Step 3: An equal number of Light and Heavy cells were mixed for each condition. Step 4: Subcellular fractionation and recovery of cytosolic and plasma membrane and organelles (PMO) fractions. Step 5: Protein extraction. Step 6: Cysteine derivatization (alkylation using iodoacetamide, reduction using 1,2‐dithiothreitol (DTT), biotin‐HPDP labeling). Step 7: Protein tryptic digestion. Step 8: Streptavidin affinity chromatography, biotinylated peptides are retained and eluted by DTT to constitute the enriched fraction containing oxidized peptides (S‐Ox), and the flowthrough is the non‐enriched fraction containing reduced peptides (S‐Red), and peptides without cysteines (called no‐S). Step 9: Liquid chromatography separation of peptides of the two fractions and MS/MS analysis. Step 10: Data Analysis. The intensities of the heavy and light peptides are used for quantification (L:H ratio). Abbreviations: CAM = carbamidomethyl, biotin‐HPDP = N‐[6‐(Biotinamido)hexyl]‐3′‐(2′‐pyridyldithio) propionamide.

The OcSILAC experiment (Figure 2) was designed to provide an overview of the protein expression levels and thiol‐redox state in these fractions. OcSILAC targets the reversible oxidation of cysteine (Cys) residues, namely sulfenic acids, S‐nitro groups, and disulfide bridges.^[^ ^30^ ^]^ The present workflow was adapted from previous applications^[^ ^28^, ^29^ ^]^ and validated (Supplementary Information 1C, Figures S.1.5–7). After sample processing, three fractions were obtained: S‐Ox containing peptides with oxidized Cys, S‐red with reduced Cys, and no‐S with no Cys (see Supplementary Information 4, Materials and Methods). The no‐S peptides were used to quantify the protein levels, and the S‐Red and S‐Ox peptides were used to evaluate the redox status.

### A 6‐h LPS Treatment Led to an Alteration of the Protein Levels but Not of the Cysteine Oxidation Levels

**Figure 3:**
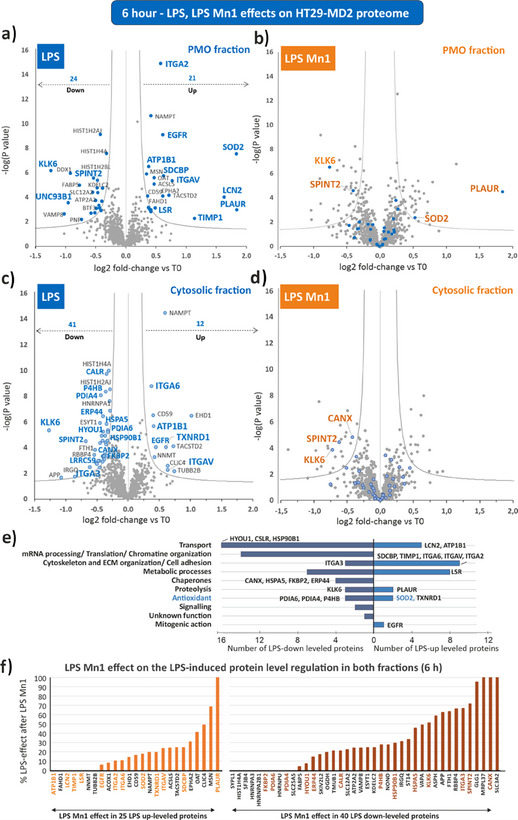
Effects of LPS and LPS **Mn1** treatment for 6 h on protein levels in comparison with untreated cells at T0. The experiments were performed in biologically independent triplicate. Volcano plots of differentially quantified proteins in the plasma membrane & organelles (PMO) fractions (a, b) or cytosolic fractions (c, d) between LPS treated cells and untreated cells (a, c), and between LPS **Mn1** treated cells and untreated cells (b, d). The y‐axis t‐test p‐value in ‐log10 scale (significant if p‐value < 0.05) is plotted against the fold‐change between two conditions in log2 scale (x‐axis). Increased levels appear with positive log2 values and decreased levels with negative log2 values. In a and c, the name of the proteins mentioned in the main text are highlighted in blue (closed and open symbols, respectively). In b and d, proteins whose level was modified by LPS (i.e., highlighted in blue in a and c) are indicated in blue (closed and open circles in b and d respectively) and those which remain modified in LPS **Mn1** condition have their name indicated in orange. See Tables S.2.1 and S.2.2, and S.2.4 for complete detailed information. e) The LPS‐regulated proteins were reported according to their biological function. The number of up‐ and down‐leveled proteins in each category is indicated in the x axis. f) Histogram representing the effect of **Mn1** on the level of the proteins modulated by LPS, expressed as a percentage. A low percentage means that **Mn1** causes the protein level to return toward basal, that is, toward levels observed at T0‐basal state (e.g., for LCN2). A high percentage means that **Mn1** did not resolve the LPS‐induced effect (e.g., for PLAUR). Proteins indicated in orange and dark brown are those discussed in the text.

At 6 h, the protein levels were affected (33 up‐leveled in comparison with basal (T0) and 65 down‐leveled proteins in both fractions, Figure 3, Tables S.2.1 and S.2.4). In contrast, only weak changes of Cys oxidation state were detected (Figure 5a, Figure S.2.2b, Tables S.2.3 and S.2.11).

As previously shown,^[^ ^19^, ^20^, ^34^, ^35^ ^]^ SOD2 was overexpressed and emerged here as one of the most overexpressed proteins in the PMO fraction, along with urokinase plasminogen activator surface receptor (PLAUR) and lipocalin‐2 (LCN2) (Figure 3a). To note, in IBD, PLAUR has been associated with the damages of the intestinal epithelial barrier,^[^ ^36^ ^]^ and LCN2, involved in metal ion sequestration, is more a biomarker of inflammation.^[^ ^37^ ^]^ The level of cytosolic thioredoxin reductase (TXNRD1), meant to reduce the disulfide bridge in thioredoxin (TXN), was enhanced (Figure 3c). The increase of SOD2 and TXNRD1, both involved in ROS detoxification, indicate indirectly an increased oxidative stress. Several proteins being able to counteract inflammation by impairment of the TLR4 trafficking and/or signaling were impacted: we observed a decreased level of the generalist ER chaperones calnexin (CANX), calreticulin (CALR), heat shock protein 90 kDa beta member 1 (HSP90B1), and hypoxia up‐regulated protein 1 (HYOU1). Moreover, an increase in syntenin‐1 (SDCBP) level, a negative regulator of TLR4 signaling,^[^ ^38^ ^]^ was recorded. Another observation is the decreased levels of protein disulfide isomerases (PDIs; PDIA4, PDIA6, and P4HB) (Figure 3c) and other ER chaperones [ER resident protein 44 (ERP44), heat shock protein A member 5 (HSPA5) and peptidyl‐prolyl cis‐trans isomerase (FKBP2)]. In line with this observation, a recent work has shown that the PDI levels may be regulated by mitochondrial ROS (mtROS) and the upregulation of SOD2 correlated with decreased levels of PDIs.^[^ ^39^ ^]^ A set of proteins contributing to the integrity of the intestinal epithelium such as integrins (ITGAV, ITGA6, ITGA2), epidermal growth factor receptor (EGFR), sodium/potassium‐transporting ATPase subunit beta‐1 (ATP1B1), and lipolysis‐stimulated lipoprotein receptor (LSR) were up‐leveled upon LPS treatment, whereas integrin ITGA3 was decreased (Figure 3a,c). These proteins, contributing to maintaining the epithelial barrier, are also believed to play a role in the innate immune response. Proteins involved in the extracellular matrix remodeling such as tissue inhibitor of metalloproteinase 1 (TIMP1), kallikrein‐6 (KLK6), and Kunitz‐type protease inhibitor 2 (SPINT2)^[^ ^40^, ^41^ ^]^ were altered upon LPS treatment (Figure 3a and c). In Figure 3e are represented the most LPS‐affected proteins, discussed here above, sorted according to their function (Table S.2.4).

**Figure 4:**
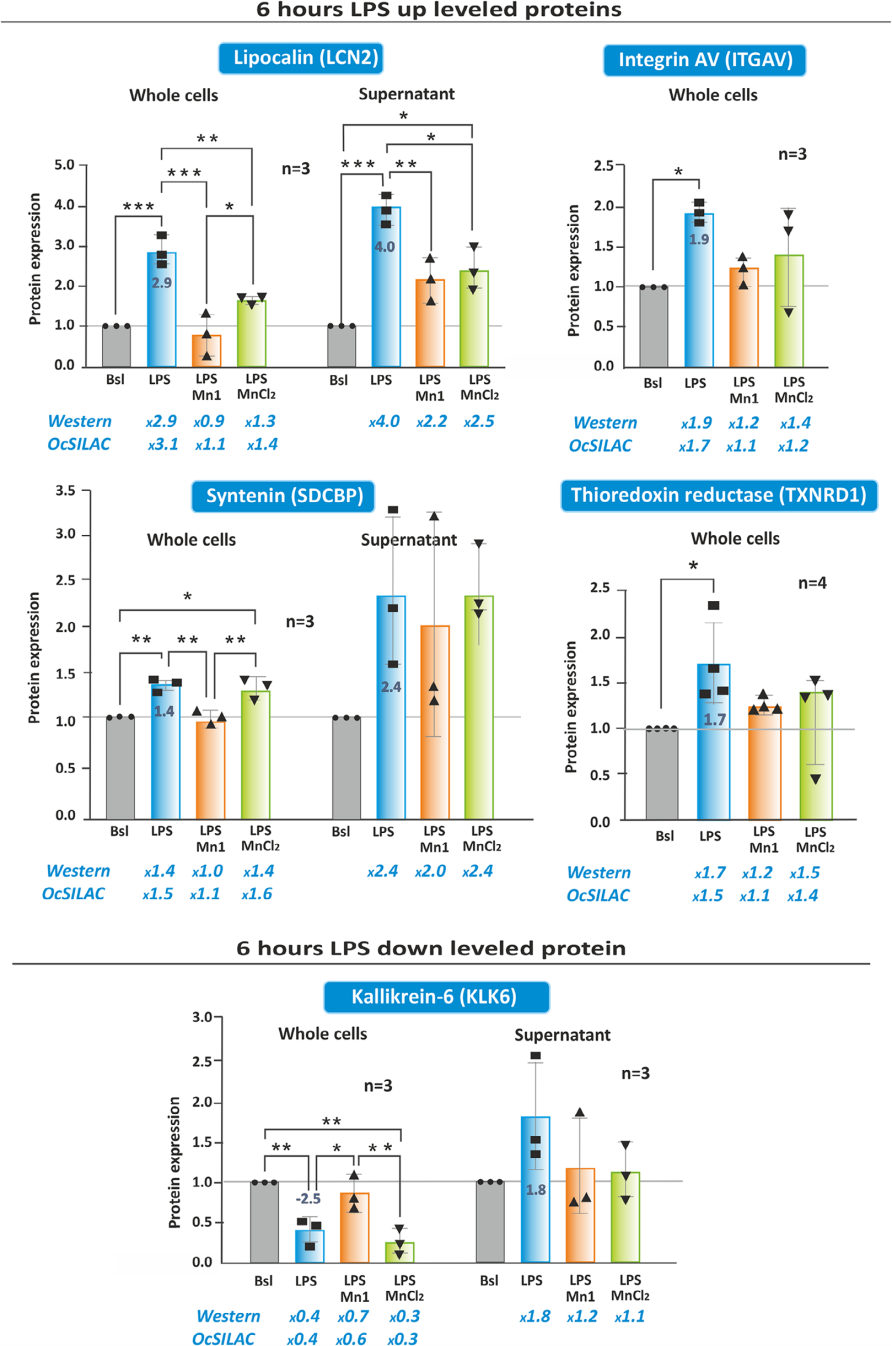
Validation of the proteomic data by Western blot with five proteins newly identified in the whole cell lysates of HT29‐MD2 cells. Proteins were quantified in cell lysates for LCN2, ITGAV, TXNRD1, SDCBP, and KLK6, and in cell culture media for LCN2, SDCBP, and KLK6. HT29‐MD2 cells were incubated for 6 h with cell culture medium, LPS (0.1 µg/mL), LPS **Mn1** (100 µM) or LPS MnCl_2_ (100 µM). See Table S.3.1 for antibody information. Full blots are shown in Figure S.3.2. The abundance of each protein was normalized to the total amount of protein in each lane. The protein level intensity was normalized by Bsl which was set at 1 for each independent experiment. Data represent mean ± SEM for n independent biological experiments, n indicated for each graph. The p‐values were calculated using the ordinary one‐way ANOVA test. The mean rank of each column was compared with the mean of every other column with (***) *p* < 0.001, (**) *p* < 0.01, and (*) *p* < 0.05.

To confirm the proteomic results, we analyzed by WB, in the whole cell lysates of HT29‐MD2 cells, the levels of five of the proteins identified as modified the most after the 6‐hour LPS treatment and relevant to oxidative stress or inflammation. As shown in Figure 4 (and Figure 1 for SOD2) results were consistent with the OcSILAC results: levels of SOD2, LCN2, ITGAV, TXNRD1, and SDCBP were increased while the one of KLK6 was decreased after 6‐h LPS treatment. Since the localization and/or known biological action of LCN2, SDCBP, and KLK6 is extracellular, we also analyzed their secretion and found that all three were more secreted after LPS treatment (Figure 4), as for IL‐8 (Figure 1).

**Figure 5:**
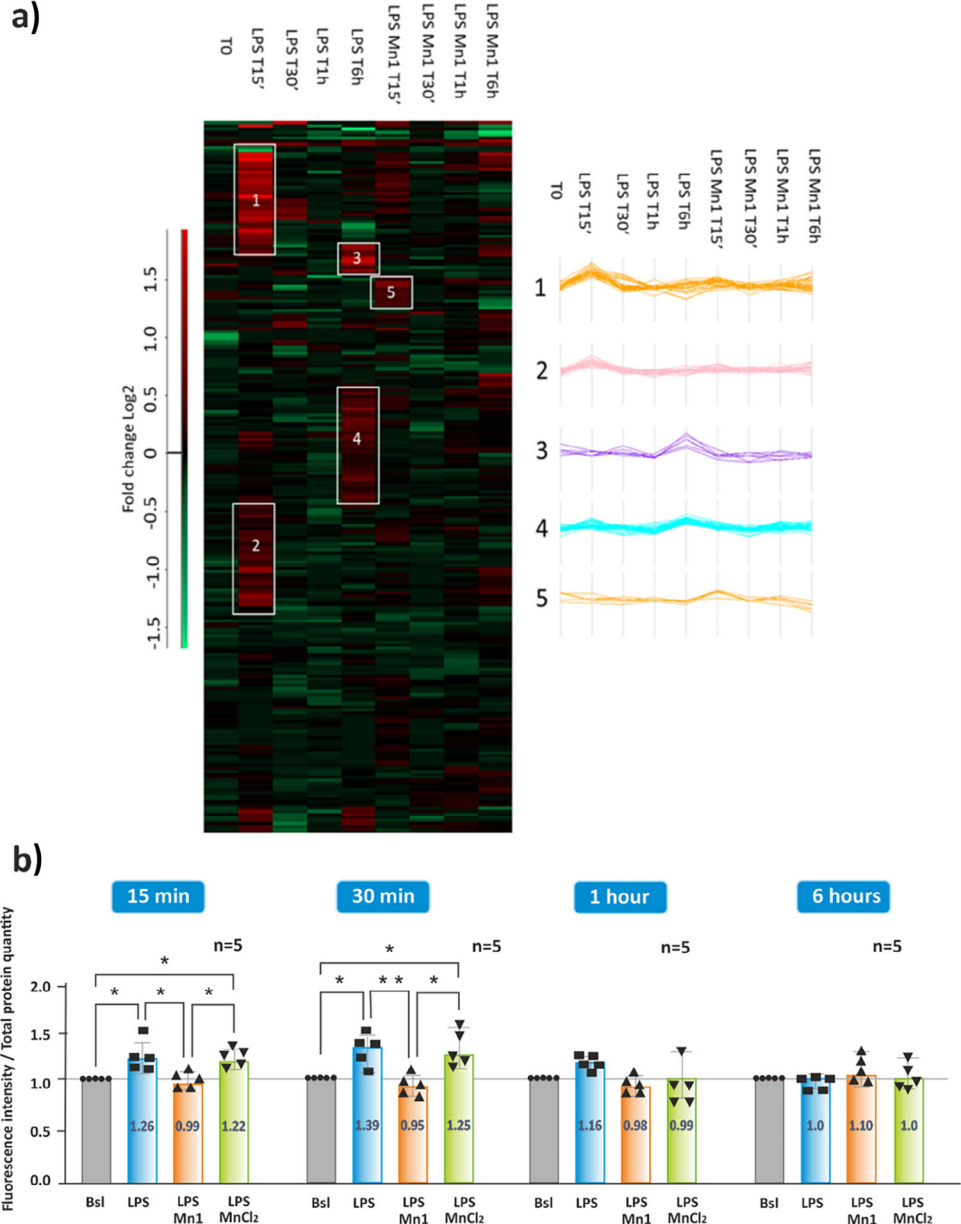
a) Left panel: Hierarchical clustering of the oxidized cysteine fold change (Log2) normalized by the associated protein expression levels. The hierarchical clustering has been performed at row level only. Each column contains the median values of the oxidized cysteine fold changes measured in three biological replicates and the two subcellular fractions. Right panel: The graphic contains the fold changes at different time points of the oxidized peptides present in the 5 clusters detected in the hierarchical cluster. b) Fluorescence gel imaging quantitation of cysteine oxidation. Fluorescence intensity was measured in HT29‐MD2 cells incubated for 15 min, 30 min, 1 h, and 6 h in cell culture medium only (Bsl), or medium containing LPS (0.1 µg/mL), LPS **Mn1** (100 µM) or LPS MnCl_2_ (100 µM). The fluorescence intensity measured for the Bsl state sample is set at 1 for each independent experiment. The fluorescence intensity was normalized to the total amount of protein in each lane as assessed by Coomassie blue densitometry. Data represent mean ± SEM for five independent biological experiments (n = 5 indicated above each set). The p‐values were calculated using the ordinary one‐way ANOVA test. The mean rank of each column was compared with the mean of every other column with (***) *p* < 0.001, (**) *p* < 0.01, and (*) *p* < 0.05. Full gels are shown in Figure S.3.4.

The normalized oxidized cysteine fold changes, defined as the fold change of the oxidized Cys containing peptide divided by the protein fold change, are represented by hierarchical clustering graphics in Figure 5a. In the condition LPS 6 h, the two clusters 3 and 4 are indicative of cysteine oxidation (in red), but the corresponding ratios were under the threshold of statistical significance (see Supplementary information 4). This shows that the level of oxidative stress is close to the basal redox status. This result could be rationalized by the activation of antioxidant proteins such as thioredoxin reductase 1 (TXNRD1) and SOD2, suggesting that there has been an occurrence of oxidative stress upon LPS activation at earlier times, but that this is resolved at 6 h. This prompted us to look at shorter times (see below, 15 min, 30 min, and 1 h). But before delineating the results at these time points, the effects of **Mn1** coincubation with LPS at 6 h are presented.

### Mn1 Coincubation Counterbalanced the Effect on the Proteome of a 6‐h LPS Challenge

The level of most of the proteins altered upon LPS treatment returned to basal upon coincubation LPS **Mn1**: on Figure 3, one can compare b to a for the PMO fraction and d to c for the cytosolic fraction. Note that the blue circles in b (respectively d) show the proteins that were altered by LPS (Figure 3f, Table S.2.2). Notably, the levels of the antioxidant enzymes SOD2 and TXNRD1, strongly increased by LPS, were not significantly altered when LPS was coincubated with **Mn1**. Similarly, PDIs and other ER chaperone levels were not decreased in the condition LPS **Mn1**, unlike when in the presence of LPS only. In contrast, PLAUR and KLK6 remained respectively over and under‐expressed in the condition LPS **Mn1**, as they were with LPS. Note that these two proteins are among the few proteins affected by **Mn1** treatment alone (Figure S.2.2a, Table S.2.2). Figure 3f sums up the mitigation effect of **Mn1** coincubation with LPS on LPS‐altered proteins. Note that MnCl_2_ was also able to limit the LPS effects but with a smaller magnitude and a profile different from **Mn1** (Figure S.2.3). These results, showing that **Mn1** coincubation mitigates the effect of LPS incubation, particularly on the level of the antioxidant proteins (SOD2, TXNRD1, for instance), are consistent with the idea that **Mn1** acts as an antioxidant, limiting the ROS flux induced by LPS, and thus the cellular feedback leading to the overproduction of SOD2^[^ ^19^, ^20^ ^]^ and TXNRD1.

As levels of antioxidant proteins, such as SOD2 or TXNRD1, were impacted (Figures 3 and 4), we decided to investigate NRF2, known to be involved in the activation of the antioxidant response element (ARE) leading to the expression of these proteins. Upon oxidative stress, the partner of NRF2, KEAP1, is oxidized leading to NRF2 accumulation and translocation into the nucleus. As shown by WB (Figure S.3.3), NRF2 was not significantly altered after a 6‐h LPS incubation, although with a tendency to increase. But, interestingly, upon coincubation of LPS with **Mn1**, the NRF2 level was strongly reduced, indicating the capacity of **Mn1** to limit oxidative stress. This result is consistent with the fact that under LPS‐induced oxidative stress conditions, SOD2 overexpression, and more generally NRF2 activation, are no more induced in the presence of **Mn1**.

It is noteworthy that **Mn1** and closely related compounds induce a decrease in SOD2 levels in LPS‐stressed cells.^[^ ^19^, ^20^, ^21^, ^31^, ^35^ ^]^ This contrasts with other reported SOD mimics, such as Mn‐porphyrins (e.g., Mn(III) meso‐tetrakis(N‐nbutoxyethyl‐pyridinium‐2yl)porphyrin Mn(III)TnBuOE‐2‐PyP^5+^), that were described to increase the levels of antioxidant enzymes under the NRF2 control, like NAD(P)H dehydrogenase [quinone] 1 (NQO1), catalase, and SOD2.^[^ ^42^ ^]^ Similarly, M40403, a Mn(II) cyclic polyamine, was reported to increase the NRF2 level.^[^ ^43^ ^]^ This was proposed to be associated with a pro‐oxidant effect linked with a fast production of high levels of H_2_O_2_ induced by the superoxide dismutation which exceed the toxicity threshold.^[^ ^42^ ^]^ These classes of SOD mimics have thus an indirect effect, additional to the direct superoxide flow control, linked to NRF2 activation possibly longer lasting.^[^ ^18^ ^]^ In contrast, **Mn1** displays a direct effect on superoxide flow through the catalysis of the superoxide dismutation, slower than the porphyrins and thus leading to a lower H_2_O_2_ level. **Mn1** plays thus an early effect, whereas the other SOD mimics mentioned display an activity that depends on protein synthesis but that may last longer, making the two classes of SOD mimics complementary.^[^ ^18^ ^]^ Interestingly, Mn(III)TnBuOE‐2‐PyP^5+^ has been shown to mitigate the effect of doxorubicin on mitochondrial dysfunction in rats, on the proteome, and on an early oxidative stress (1 h).^[^ ^44^ ^]^

### OcSILAC at Short Time Points (15 min) Revealed that LPS Induced a Proteome‐Wide Cys Oxidation that was Mitigated by Mn1

The fact that no or weak effect on the Cys oxidation was recorded at 6 h LPS (Figure 5a, Tables S.2.3 and S.2.11) could be due to the fact that the LPS‐induced oxidative phase occurring at earlier time points was resolved at 6 h to a large extent. We thus performed proteomic and redoxomic analyses at earlier timescales, namely 15 min, 30 min, and 1 h.

**Figure 6:**
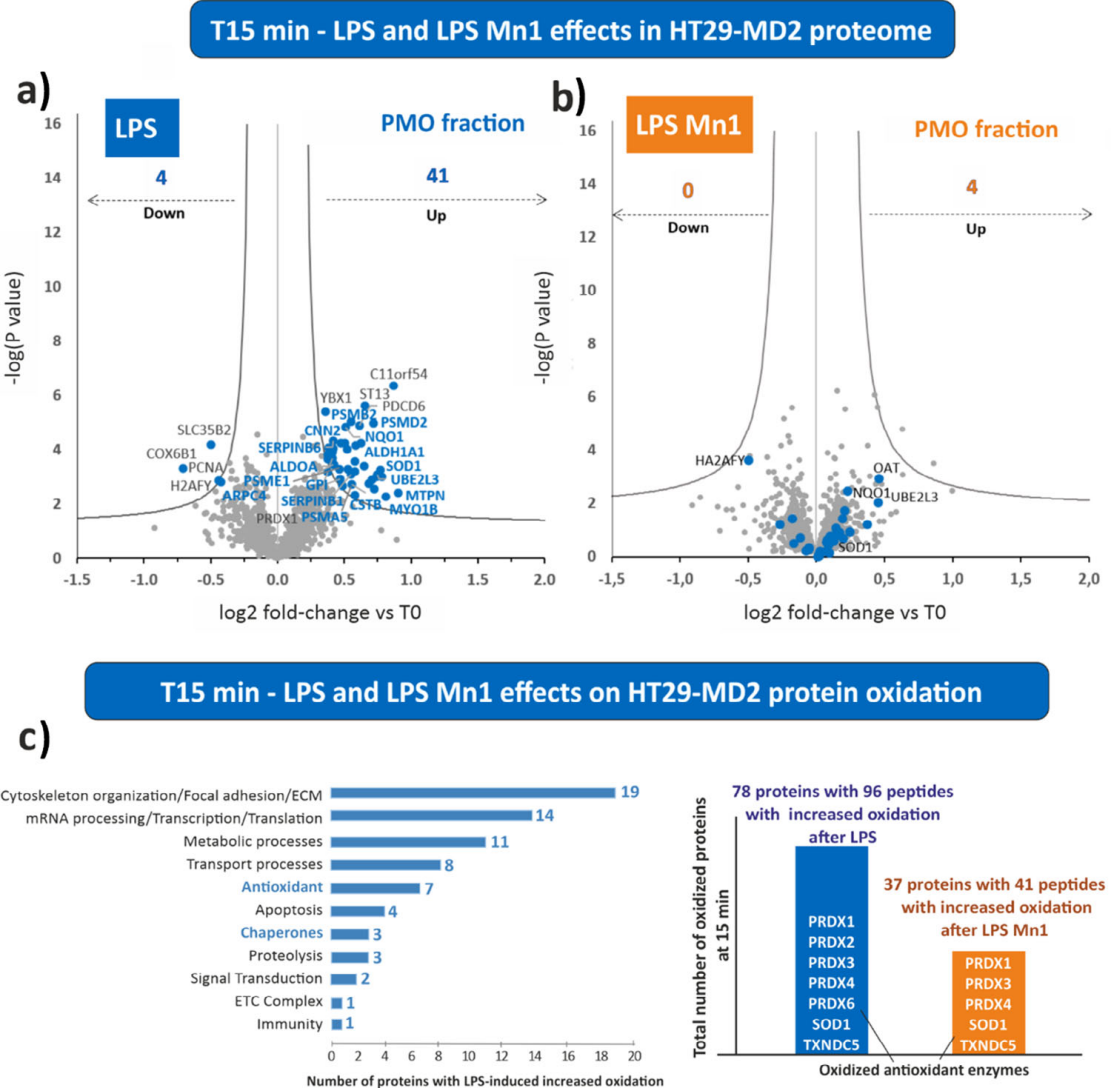
Effects of LPS and LPS **Mn1** treatment for 15 min. a) and b): effects on protein levels in comparison with untreated cells at T0. Volcano plots of differentially quantified proteins in the plasma membrane & organelles (PMO) fractions between LPS treated cells and untreated cells (a) and between LPS **Mn1** treated cells and untreated cells (b). The y‐axis t‐test p‐value in ‐log10 scale (significant if p‐value < 0.05) is plotted against the fold‐change between two conditions in log2 scale (x‐axis).^[^ ^47^ ^]^ Increased levels appear with positive log2 values and decreased levels with negative log2 values. In a, the name of the proteins mentioned in the main text are highlighted in blue. In b, proteins whose level was modified by LPS (i.e., highlighted in blue in a) and whose level has returned to that of untreated conditions in LPS **Mn1** condition are indicated in blue. See Figure S.2.4, Tables S.2.4 and S.2.5 for complete detailed information. c) LPS and LPS **Mn1** effects on protein oxidation status in both PMO and cytosolic fractions. Left: LPS‐oxidized proteins reported according to their biological function, right: LPS‐oxidized proteins in the LPS **Mn1** sample. The LPS condition is shown in blue, and the LPS **Mn1** condition is shown in orange.

After 15 min of LPS stimulation, 45 protein levels modifications were detected (Figure 6, Figure S.2.4 a‐b, Table S.2.5 and references therein). Pathway analysis (Reactome Pathway database, Table S.2.4) revealed that four of them are involved in the operation of the innate immune system (MIF, LTA4H, APRT, TSPAN6). Increased levels of proteasome proteins (PSMD2, UBE2L3, PSMA5, PSMB2, PSME1) involved in the processing of antigens for the presentation through the MHC class I pathway and in the inflammation regulating the NF‐κB pathway were also detected. Associated with the inflammatory response, our results also evidenced the increased levels of a set of proteinase inhibitor proteins such as serpin‐B1 (SERPINB1), serpin‐B6 (SERPINB6), cystatin‐B (CSTB) that represent a defense against pathogen proteases and they are regulators of cathepsin pro‐inflammatory activity. Filament reorganization is also a well‐known effect of LPS cell exposure: in the present study, we observed the modulation of a set of cytoskeleton proteins, such as myotrophin (MTPN), myosin‐Ib (MYO1B), calponin 2 (CNN2), and actin‐related protein 2/3 complex subunit 4 (ARPC4). In addition, we detected increased levels of ROS‐detoxifying proteins (SOD1, NQO1). Notably, SOD1 was found increased in the mitochondria and not in the cytosol. A similar observation was already described in the presence of antimycin A known to induce a superoxide increase in the mitochondria, with a trapping of SOD1 in this organelle.^[^ ^45^, ^46^ ^]^ The level of retinal dehydrogenase 1 (ALDH1A1) known to attenuate oxidative stress was also increased. It was shown in the literature (see Table S.2.5 for references) that the expression of ALDH1A1 was increased in macrophages as part of the early acute‐phase inflammatory response, and induction of its expression contributed to the inflammatory phenotype of Crohn's in IBD patients. The glycolytic enzymes glucose‐6‐phosphate isomerase (GPI) and fructose‐bisphosphate aldolase A (ALDOA), compatible with NRF2 activation, are also increased and evidenced the activation of a cellular response against oxidative stress.

Cysteine redoxomic analysis of LPS‐treated cells after 15 min evidenced an increase in oxidation of cysteines (Figures 5a and 6c, Tables S.2.6 and S.2.11). More specifically, we observed increased oxidation levels of the resolving cysteines of the cytoplasmic and the mitochondrial peroxiredoxins, which are part of the first antioxidant line against H_2_O_2_ (PRDX1 Cys^173^, Cys^71^, Cys^83^, PRDX2 Cys^172^, PRDX3 Cys^229^, PRDX6 Cys^91^), and of the ER peroxiredoxin (PRDX4 Cys^51^). However, we did not observe alterations of the catalytic cysteines of TXN and TXNRD1, suggesting that the cellular antioxidant system is not overloaded. A set of plasma transmembrane proteins (EPCAM Cys^135^, APMAP Cys^149^, ITGB1 Cys^691^, CD46 Cys^157^) showed increased levels of oxidation. We also observed that the cysteine Cys^231^ of KLK6 was oxidized.

Interestingly, almost all the LPS‐regulated proteins at 15 min were at normal levels when LPS was coincubated with **Mn1** (Figure 6b). In addition, the analysis of the protein levels showed no increase in the level of inflammatory factors (see above: SERPINB1, SERPINB6, CSTB) in contrast to what was observed with LPS alone. This result agrees with the limitation of IL‐8 secretion reported in Figure 1 and previously.^[^ ^19^, ^20^, ^21^, ^35^ ^]^ Furthermore, no statistically significant cysteine oxidation was detected in the presence of **Mn1,** in contrast with LPS alone (Figure 5a and Table S.2.6). Globally, this shows at different levels (proteome, cysteine oxidation) that coincubation of LPS with **Mn1** mitigates the effects induced by LPS alone as early as 15 min.

We also investigated 30 min and 1 h of incubation. After LPS treatment very weak effects were recorded in terms of protein levels (Figures S.2.5 and S.2.7, Tables S.2.7 and S.2.9) or in terms of Cys oxidation level (Figure 5a, Tables S.2.8, S.2.10, and S.2.11). Interestingly, after LPS **Mn1** coincubation for 30 min and/or 1 h, a set of ER proteins (P4HB, PDIA4, PDIA6, ERP44, HYOU1, TXND5) involved in oxidative folding and other redox‐mediated processes, were found down‐leveled, as already observed at 6 h LPS treatment (Figures S.2.5, S.2.6, and S.2.7, Tables S.2.7 and S.2.9). This effect was not observed in cells exposed to **Mn1** or LPS alone (30 min, 1 h). Furthermore, cysteine oxidation was found lower than at 15 min (Figure 5a, Tables S.2.8 and S.2.10).

To confirm the OcSILAC data, we performed quantification of LPS‐induced Cys oxidation by gel fluorescence quantitation^[^ ^30^ ^]^ of whole cell lysates. In a kinetic study using a maleimide fluorophore to specifically label the S‐Ox species, the Cys oxidation was increased after 15 min (1.3‐fold) and 30 min (1.4‐fold) of LPS stimulation. LPS **Mn1**, but not LPS MnCl_2_, limited this observed oxidation. The oxidation appeared resolved at 6 h (Figure 5b and Figure S.3.4). These results are consistent with those described for the Cys redoxome analysis using OcSILAC (Figure 5a).

Overall, these results are consistent with our hypothesis that **Mn1** displayed an antioxidant activity in cells, as seen with Cys oxidation levels at 15 min (Figure 5a, Table S.2.6). As previously suggested,^[^ ^19^ ^]^ the presence of **Mn1** allows an antioxidant early effect on superoxide. In a way, this early action anticipates the antioxidant effect of SOD2 that is dependent on enzyme biosynthesis induced by LPS treatment at longer time points.

## Conclusion

In the present study, we investigated the outcomes of LPS activation in the epithelial intestinal cell line HT29‐MD2,^[^ ^22^, ^48^ ^]^ together with the role of the SOD‐mimic **Mn1**, previously reported for its antioxidant and anti‐inflammatory activity.^[^ ^19^, ^20^, ^21^, ^35^, ^49^, ^50^ ^]^ For that purpose, HT29‐MD2 cells were incubated with LPS or LPS **Mn1**. LPS MnCl_2_ and **Mn1** alone were also studied as controls and experiments were performed at different time points (15 min, 30 min, 1 h, 6 h).

**Figure 7:**
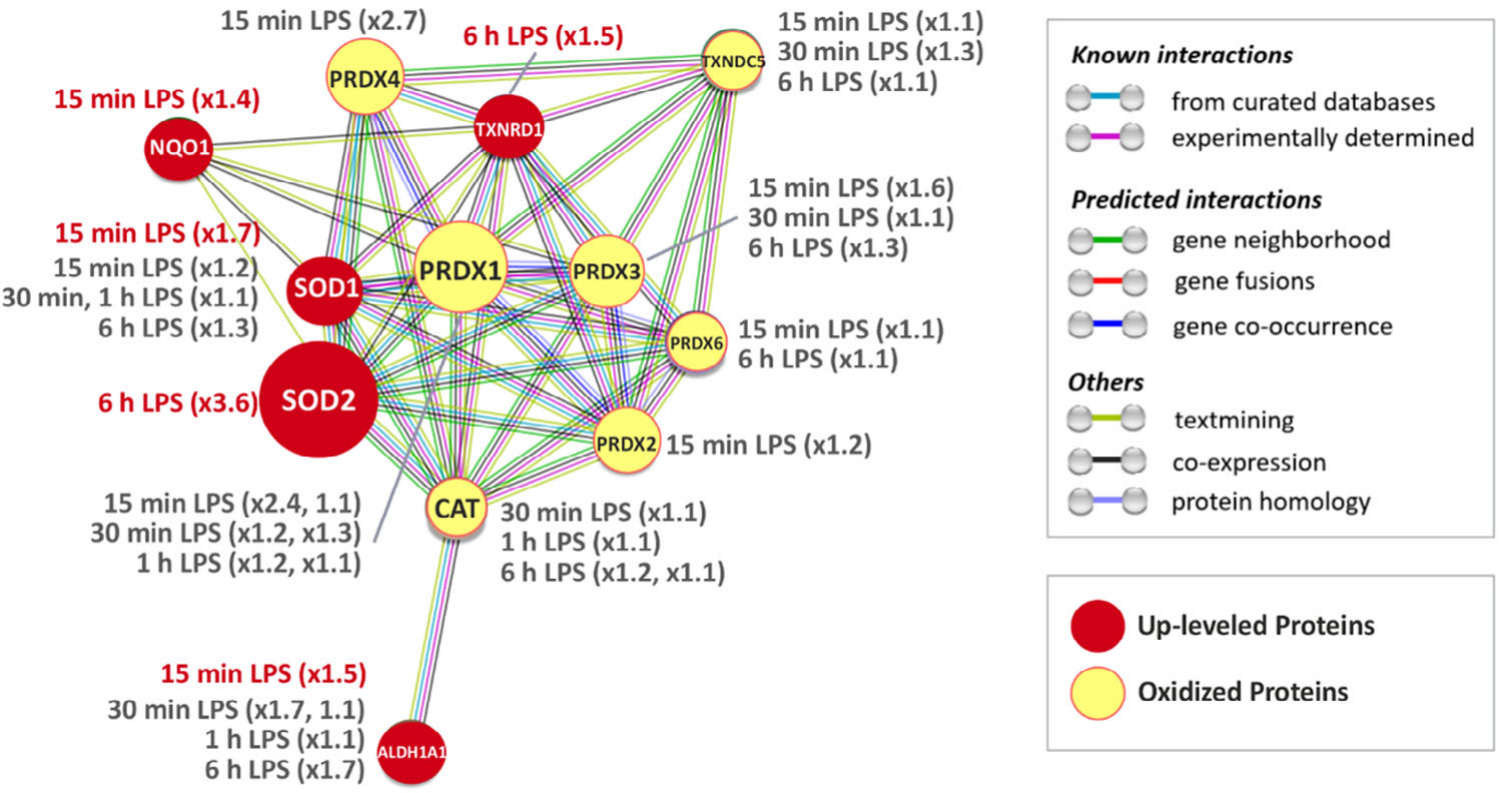
Modified antioxidant enzymes. Proteins up‐regulated after LPS treatment are in red (levels indicated in red), and oxidized proteins are in yellow (levels indicated in grey). The protein network connects different ROS scavenger proteins and we can distinguish the early LPS response via thiol chemistry and the late response by the increase of protein expression. The size of the nodes is proportional to the more significant fold change. Biological clustering of proteins was performed with the STRING database V.12 (FDR ≤ 0.05, strength ≥ 0.01). The nodes represent proteins and the edges represent both functional and physical protein‐protein associations based on known and predicted interactions.

So far, few studies involving proteomic and/or redoxomic analyses have been recorded on SOD mimics, notably using shotgun proteomics as here.^[^ ^51^, ^52^, ^53^, ^54^, ^55^, ^56^ ^]^ In this work, the mass‐spectrometry analyses were performed in two cell fractions (cytosol and plasma membrane & organelles), the fractionation allowing more proteins to be identified. This kinetic redox proteomic analysis enables the identification of biologically important protein alterations (either expression levels or cysteine oxidations) upon LPS activation. Interestingly, most of them were associated with the regulation of oxidative stress (Figure 7) or inflammation. LPS was shown to induce an early oxidative stress effect (15 min) that preceded inflammation. This resulted in the activation of an antioxidant cell feedback in which SOD2 is the main over‐expressed protein at 6 h. These findings support the fact that oxidative stress and inflammation are closely intertwined and, more precisely, that oxidative stress is at the onset of the inflammatory response. Importantly, coincubation with **Mn1** was shown to mitigate the effects observed under LPS stimulation, both at the early oxidation level and at the later protein level picture. The early effect of **Mn1** evidenced in the present work advocates for a direct effect of this SOD mimic on the ROS concentration. This early effect of **Mn1** also advocates for a link between an early oxidation induced by LPS and a later inflammation. Indeed, the most important class of the LPS‐modified proteins were those involved in antioxidant defense, with the preponderant increased levels of SOD2 at 6 h. Note that the effects, notably on oxidative stress, are moderate: this was anticipated as the intestinal epithelial HT29‐MD2 cell line is not meant to produce high levels of ROS, as could have done macrophages. However, the relevance of this cell line to reductionist models of IBD makes them interesting to study. The powerful OcSILAC approach allowed us to monitor these moderate but biologically relevant effects. This showed an unambiguous direct link between oxidative stress and inflammation: they are not only associated but oxidative stress precedes inflammation.

ROS are central in inflammation signaling; impaired redox homeostasis is a key mechanism of IBD progression.^[^ ^12^ ^]^ Currently used medications can cause multiple side effects and do not provide sufficient control over the illness. Antioxidant substances like SOD mimics,^[^ ^57^, ^58^ ^]^ as catalytic antioxidant agents, acting at the onset of inflammation, could probably be a useful contribution to the therapeutic armamentarium for IBD.

## Conflict of Interests

Philippe Seksik received consulting fees from Takeda, Abbvie, Merck‐MSD, Biocodex, Janssen, Amgen, Astellas and Pfizer and grants from Biocodex and Janssen but declares no conflict of interest for the present work. The other authors declare no conflict of interest.

## Supporting information



Supporting Information

## Data Availability

The data that support the findings of this study are openly available in ProteomeXchange. ProteomeXchange accession number: PXD05241. FTP download: https://ftp.pride.ebi.ac.uk/pride/data/archive/2025/03/PXD052414.
