# Supplementary material for: Kinetic Redox Shotgun Proteomics Reveals Specific Lipopolysaccharide Effects on Intestinal Epithelial Cells, Mitigated by a Mn Superoxide Dismutase Mimic
Source: Angew Chem Int Ed Engl. 2025 Mar 22;64(19):e202422644. doi: 10.1002/anie.202422644 (PMC12051783; doi:10.1002/anie.202422644)
Supplement: Supplementary file 1 — Supporting Information [file ANIE-64-e202422644-s001.pdf]

Kinetic Redox Shotgun Proteomics Reveals Specific Lipopolysaccharide Effects on Intestinal Epithelial Cells, Mitigated by a Mn Superoxide Dismutase Mimic

Martha Zoumpoulaki [1-3], Giovanni Chiappetta [2], Jean Bouvet [1], Namita-Raju John [1], Gabrielle Schanne [1,3], Pauline Gehan [1], Samuel Diebolt [2], Shakir Shakir [2], Elodie Quévrain [1,3], Emilie Mathieu [1], Sylvie Demignot [3,4], Philippe Seksik [3], Nicolas Delsuc [1], Joelle Vinh [2,*] and Clotilde Policar [1,*]

1 Laboratoire Chimie Physique et Chimie du Vivant—CPCV, UMR8228, Département de Chimie, Ecole Normale Supérieure, PSL University, Sorbonne Université, CNRS, 75005 Paris, France
2 Laboratoire de Spectrométrie de Masse Biologique et Protéomique, SMBP, UAR2051, ESPCI Paris, PSL University, CNRS, 75005 Paris, France
3 Gastroenterology Department, Sorbonne Université, INSERM, Centre de Recherche Saint-Antoine, CRSA, Paris Center for Microbiome Medicine (PaCeMM) FHU, AP-HP, Saint-Antoine Hospital, Paris, France
4 EPHE, PSL University, 75014 Paris, France
* Correspondance e-mail : joelle.vinh@espci.psl.eu (0000-0001-7184-2668) & clotilde.policar@ens.psl.eu (0000-0003-0255-1650)

Content:

SI1. Supplementary Information 1: validation of proteomic experiments

Abbreviations

A. SILAC proteomics in whole cells

Figure S.1.1 Validation of the SILAC experiment: cell culture. a. Effect of SILAC media on cell viability measured by MTT assay. **b.** Labeling efficacy % distribution of heavy K8 and R6 labeled proteins or peptides of HT29-MD2 heavily-grown cells. **c** Digestion efficiency by visualization of MS1 data.

Figure S.1.2 Validation of the SILAC experiment: mass spectrometry a. Total number of identified and quantified proteins of whole HT29-MD2 cells. **b.** Distribution of quantified proteins in T0 sample by a Venn diagram

Table S.1.1 Up- and down-leveled proteins in LPS, LPS **Mn1,** and **Mn1** whole cell samples, classified according to their associated biological processes

B. OcSILAC (Oc: Oxydized cysteine) validation in five subcellular fractions

Figure S.1.3 Validation of the subcellular fractionation a. Subcellular protein fractionation workflow**. B.** Fractionation efficiency of the OcSILAC adapted fractionation protocol in HT19-MD2 cells.

Figure S.1.4 Gene ontology cellular compartment enrichment on proteins specific to whole cells and fractioned cells

C. OcSILAC final experiment in two subcellular fractions

Figure S.1.5 Set-up of the OcSILAC conditions and workflow in whole cells. a Fluorescence imaging in blue light (460 nm). DyLight 448 dye excitation max at 493 nm and emission max at 518 nm. **b** Colorimetric imaging after 2 h Coomassie blue staining and five washes with Milli-Q water.

Figure S.1.6 Proteomics metadata

Figure S.1.7 Reproducibility and population differentiation by scatter plot representation.

Bibliography SI1

SI2. Supplementary Information 2: Analyses of the Proteomic and Redoxomic Data

A. 6 h

LPS, LPS Mn1, Mn1 alone and LPS $MnCl_2$ effects on the proteome level and Cys oxidation level, after 6h-incubation

Figure S.2.1 Comparison of the effects of LPS and LPS Mn1 6h-incubation vs. T0 sample (non incubated, basal sample) on the protein levels.

Figure S.2.2 Comparison of LPS Mn1 and Mn1, 6-h incubation.

a Effects on protein levels of a 6h-incubation sample **with LPS Mn1, and Mn1 (**vs. T0 sample) in the PMO and cytosolic fractions of HT29-MD2 cells.

b LPS and LPS **Mn1** effects on protein oxidation status in both PMO and cytosolic fractions.

Figure S.2.3 Comparison of LPS Mn1 and $MnCl_2$, 6-h incubation.

a LPS **Mn1** and **LPS $MnCl_2$** effects on protein levels

b Comparison of the effects on LPS **Mn1** with those of LPS $MnCl_2$, in preventing/attenuating the LPS-induced protein level modification, in PMO and cytosolic fractions

Table S.2.1 Up- and down-leveled proteins after 6-h LPS**-**stimulation of HT29-MD2 cells (vs. T0 sample (non-incubated, basal sample).

Table S.2.2 Up- and down-leveled proteins after 6-h LPS **Mn1, Mn1** alone, and LPS $MnCl_2$-stimulation of HT29-MD2 cells, vs. T0 (non-incubated or basal sample).

Table S.2.3 Proteins oxidation level after 6-h LPS-stimulation of HT29-MD2 cells, in the PMO and cytosolic fraction, compared with the corresponding LPS **Mn1** and LPS $MnCl_2$ conditions.

Table S.2.4 Up-regulated proteins after 15-min and 6-h LPS incubation vs. T0 (see Supplementary Table S.2.1, and S.2.5), classified according to their biological effect.

B. 15 min

B.1. LPS, LPS Mn1 effects on the proteome level, after 15-min incubation

Figure S.2.4 Comparison of LPS and LPS Mn1, 15-min incubation

a. Effects on protein levels of 15-min incubation of LPS, and LPS Mn1 (vs. T0 sample) in cytosolic fractions of HT29-MD2 cells.

b. LPS and LPS **Mn1** effects on protein oxidation status in both PMO and cytosolic fractions.

Table S.2.5 Up- and down-leveled proteins after 15-min LPS- and LPS **Mn1-**stimulation of HT29-MD2 cells, vs. T0 (non-incubated or basal sample).

B.2. LPS and LPS Mn1 effects on the protein oxidation level, after 15-min incubation

Table S.2.6 Proteins with increasing oxidation level after 15-min LPS**-**stimulation of HT29-MD2 cells, in the PMO and cytosolic fraction compared to the corresponding LPS **Mn1** conditions.

C. 30 min

C.1. LPS, LPS Mn1 effects on the proteome level, after 30-min incubation

Figure S.2.5 Comparison of LPS, LPS Mn1 and Mn1 (30-min incubation). Effects of 30-min incubation of LPS, LPS **Mn1**, and **Mn1** alone (vs. T0 sample)

a. on the proteome

b. on the Cys oxidation levels oxidation status

Figure S.2.6 Comparison of LPS Mn1 and LPS $MnCl_2$, 30-min incubation, proteins levels (vs. T0 sample)

Table S.2.7 Up- and down-leveled proteins after 30-min LPS, LPS **Mn1**, LPS $MnCl_2$, and **Mn1** alone stimulation of HT29-MD2 cells, vs. T0 (non-incubated or basal sample).

C.2. LPS and LPS Mn1 effects on the Cys oxidation levels after 30-min incubation

Table S.2.8 Proteins with increasing oxidation level after 30-min LPS stimulation of HT29-MD2 cells, in the PMO and cytosolic fractions, were compared to the corresponding LPS **Mn1** and LPS $MnCl_2$ conditions.

D. 1 h

D.1. LPS, LPS Mn1 effects on the proteome level and Cys oxidation level, after 1-h incubation

Figure S.2.7 Comparison of LPS and LPS Mn1, 1h on protein levels and oxidation status (vs. T0 sample). a Effects on the proteome
b Effects on protein oxidation status in both PMO and cytosolic fractions.

Table S.2.9 Up- and down-leveled proteins after 1 hour of LPS and LPS **Mn1** stimulation of HT29-MD2 cells, vs. T0 (non-incubated or basal sample).

D.2. LPS and LPS Mn1 effects on the protein oxidation level, after 1-h incubation

Table S.2.10 Proteins with increasing oxidation level after 1-h LPS stimulation of HT29-MD2 cells, in the PMO and cytosolic fractions, were compared to the corresponding LPS **Mn1** conditions.

Table S.2.11 Proteins with increasing oxidation level after 15-min to 6-h LPS-stimulation of HT29-MD2 cells, in the PMO and cytosolic fractions.

Bibliography SI2

SI3. Supplementary Information 3: Western Blots and Gels

Supplementary Table S.3.1 Selected antibodies for proteomic data validation by Western blot quantitation

Supplementary Figure S.3.1 Kinetic study of SOD2 expression in LPS-activated HT29-MD2 cells

Supplementary Figure S.3.1-A and B Kinetic study of SOD2 expression in LPS-activated HT29-MD2 cells. Western blots

Supplementary Figure S.3.2 Full Western blots for validation of proteomic data for the selected proteins (LCN2, ITGAV, SDCBP, TXNRD1, KLK6, SOD2) after incubation of HT29-MD2 cells for 6 h

Supplementary Figure S.3.3 Western Blot quantitation of NRF2 in cell lysates

Supplementary Figure S.3.4 Gel imaging to evaluate total oxidation in whole HT29-MD2 cells incubated a. 15 min, b. 30 min, c. 1 h, d. 6 h, with cell media, LPS (0.1 µg/mL), LPS Mn1 (100 µM), LPS $MnCl_2$ (100 µM)

Bibliography of SI3

SI4. Supplementary Information 4: Materials and Methods

UV-Vis spectroscopy
HT29-MD2 cell culture
LPS cell activation
Protein quantitation
IL-8 quantitation
Western blot
SILAC metabolic labeling
SILAC cell treatment
Media cytotoxicity (MTT assay)
Incorporation rate
Protein extraction from whole cells
Protein extraction from subcellular fractioned cells
Protein Digestion
Enrichment of oxidized cysteine-peptides, OcSILAC protocol in fractioned cells
LC-MS/MS
Data analysis
Fluorescence gel imaging

Bibliography of SI4

Supplementary Information 1

Validations of Proteomic Experiments

Abbreviations

BCA: Bicinchoninic acid assay
Biotin-HPDP: N-[6-(biotinamido)hexyl]-3'-(2'-pyridyldithio)propionamide
BSA: Bovine serum albumin
DMEM: Dulbecco's modified eagle medium
DPBS: Dulbecco's phosphate buffered saline
DTT: Dithiothreitol
EDTA: 2,2',2'',2'''-(Ethane-1,2-diyldinitrilo)tetraacetic acid (Ethylenediaminetetraacetic acid)
ELISA: Enzyme-linked immunosorbent assay
ER: Endoplasmic reticulum
ETC: Electron transfer chain
FBS: Fetal bovine serum
HEPES: 4-(2-hydroxyethyl)-1-piperazineethanesulfonic acid
HRP: Horseradish peroxidase
IAM: Iodoacetamide
IBD: Inflammatory bowel diseases
IECs: Intestinal epithelial cells
IL-8: Interleukin-8
LC-MS/MS: Liquid chromatography-tandem mass spectrometry
LPS: Lipopolysaccharide
MnSOD: Manganese superoxide dismutase (SOD2)
MTT: 3-(4,5-dimethylthiazol-2-yl)-2,5-diphenyltetrazolium bromide
OcSILAC: Oxidized cysteine SILAC
PBS: Phosphate-buffered saline
ROS: Reactive oxygen species
SDS: Sodium dodecyl sulfate
SILAC: Stable isotope labeling by amino acids in cell culture
SOD(2): Superoxide dismutase (MnSOD)
TCA: trichloracetic acid
TMB: 3,3',5,5'-tetramethylbenzidine

A. SILAC proteomics in whole cells

A SILAC proteomic experiment was performed on HT29-MD2 whole-cell extracts. Heavy cells, grown in heavy cell media with $^{13}C^{15}N$-Lys (Lys8) and ^{13}C-Arg (Arg6) were used as basal cells (no treatment), whereas light cells grown in light cell media were treated with LPS, LPS+**Mn1**/$MnCl_2$, or **Mn1** at 6 h. The SILAC labeling did not bias the detected proteome (Fig. S.1.1a), reaching 90-95 % labeling efficiency, controlled using heavy untreated cells (Fig. S.1.1b).[1] After successful tryptic digestion, the proteolytic peptides followed a Gaussian distribution (Fig. S.1.1c).[1] In the combined results of three independent biological experiments for T0 (light and heavy cells in light or heavy cell media at 0 min) 1,485 proteins were identified (detected either in the light or heavy fraction), among which 1,340 proteins (90 %) were quantified (detected both in the light and heavy fraction, therefore possessing an L/H ratio), with a four magnitude order dynamic range of distribution of the quantified protein intensities (Fig. S.1.2a) similarly to other proteomic experiments.[2–6] Up to 36 % (856) of the quantified proteins were quantified in three biological replicates and 59 % (1,415) in at least two replicates, and the ratio distribution (L/H) of the quantified proteins showed that more than 90 % are close to 1 in the three replicates (Fig. S.1.2b), in agreement with bibliography.[7] The number of identified proteins was equivalent to previously reported SILAC proteomic experiments in whole cells.[4,5,6] These results validated the SILAC protocol for the HT29-MD2 whole-cell proteome.

The fold-changes of LPS-regulated proteins vs. T0 were up to 2.5 at 6 h LPS (Supplementary Table S.1.1). There were few mitochondrial LPS-regulated proteins (4.5 % of the total LPS-regulated proteins, i.e. 5 out of 112 proteins) but, interestingly, they had the highest fold-changes vs. T0. Noticeably, SOD2 was not identified despite being previously overexpressed upon LPS activation. Whole cells bottom-up proteomics did not reach the required in-depth proteome detection, especially at the mitochondrial level, the main source of ROS and antioxidant defense enzymes. Interestingly, LPS **Mn1** limited the 6 h LPS effect for most regulated proteins, while **Mn1** alone had a weak impact on the protein level regulation at 6 h.

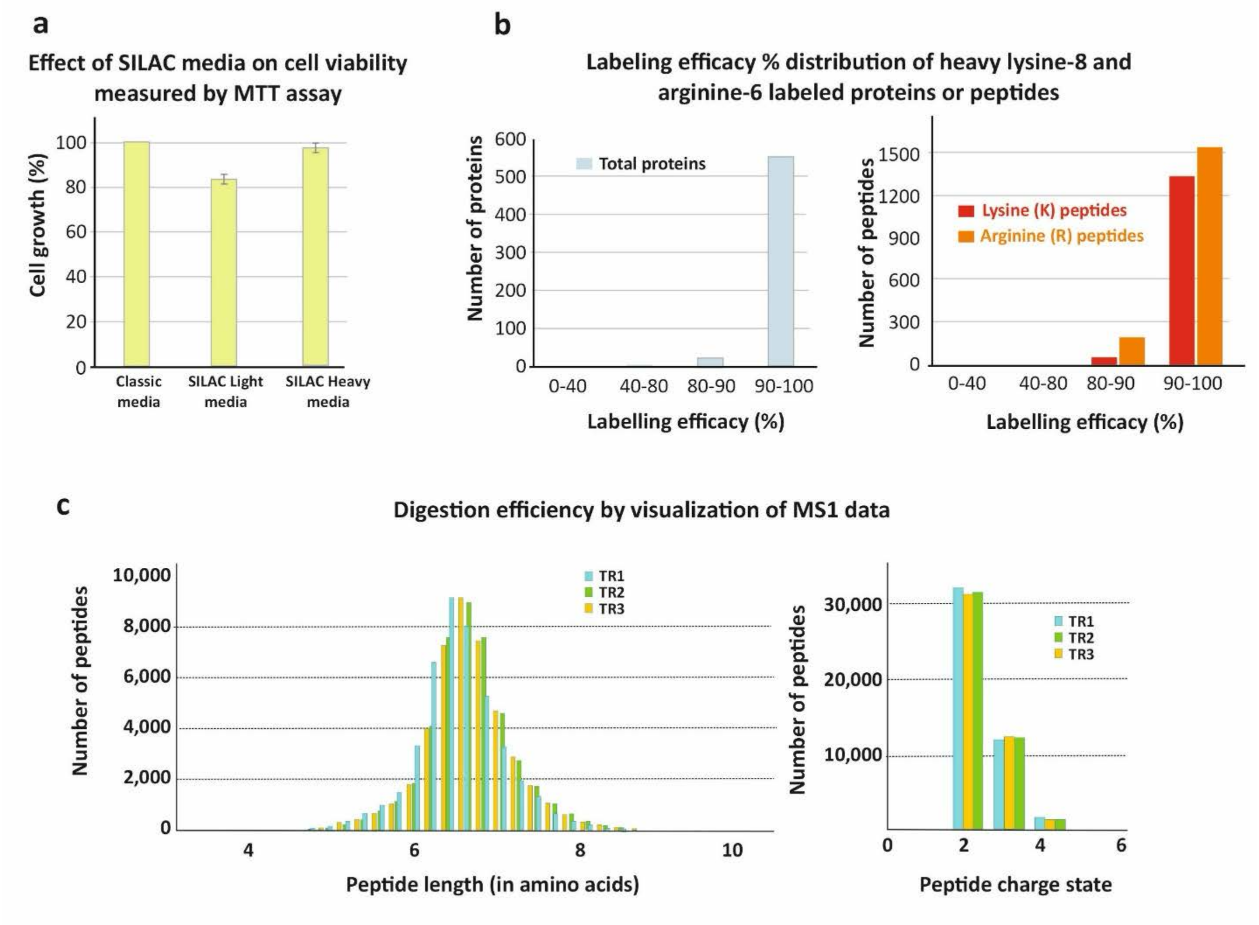

Figure S.1.1 Validation of the SILAC experiment: cell culture. a Effect of SILAC media on cell viability measured by MTT assay. HT29-MD2 cells incorporating light K0, R0, and heavy K8, R6, compared to cells grown in classic cell media. The values for cells grown in heavy and light SILAC media are normalized % to cells grown in classic media at 37 °C under a 5 % CO_2 atmosphere. Data represent ± SEM for four TR in two absorbance measurement repetitions. **b** Labeling efficacy % distribution of heavy K8 and R6 labeled proteins or peptides of HT29-MD2 heavily-grown cells. Total proteins are distributed according to their labeling efficiency %, and peptides that were trypsinated after K8 or after R6 are distributed according to their efficiency % to incorporate heavy amino acids. Results on one of the five independent heavy cell growing experiments. Proteins were identified using the MaxQuant software, and the intensities [heavy (H) and light (L), if there exist] of all the identified peptides are used to estimate the efficacy of heavy labeling, according to the following equation: $\mathbf{Labeling\ efficacy}\ (\%) = \mathbf{Intensities}\left[\frac{\mathbf{H}}{(\mathbf{H+L})}\right] \times \mathbf{100}$. A 95 % incorporation rate, which is essential for reliable quantification results. **c** Digestion efficiency by visualization of MS1 data. Proteins were digested by the serine protease trypsin (1 µg/mL), and the proteolysis is carried out in ammonium bicarbonate 50 mM, pH 7, 18 h at 37 °C. HT29-MD2 cells were analyzed by LC-MS/MS in TR following the SILAC procedure. MS1 data were visualized by the RawMeat discontinued) data quality assessment software from Vast Scientific. Peptides were evenly distributed following a Gaussian distribution with a maximum 6 to 7 amino acids. Results represent data by LC-MS/MS in three TRs.

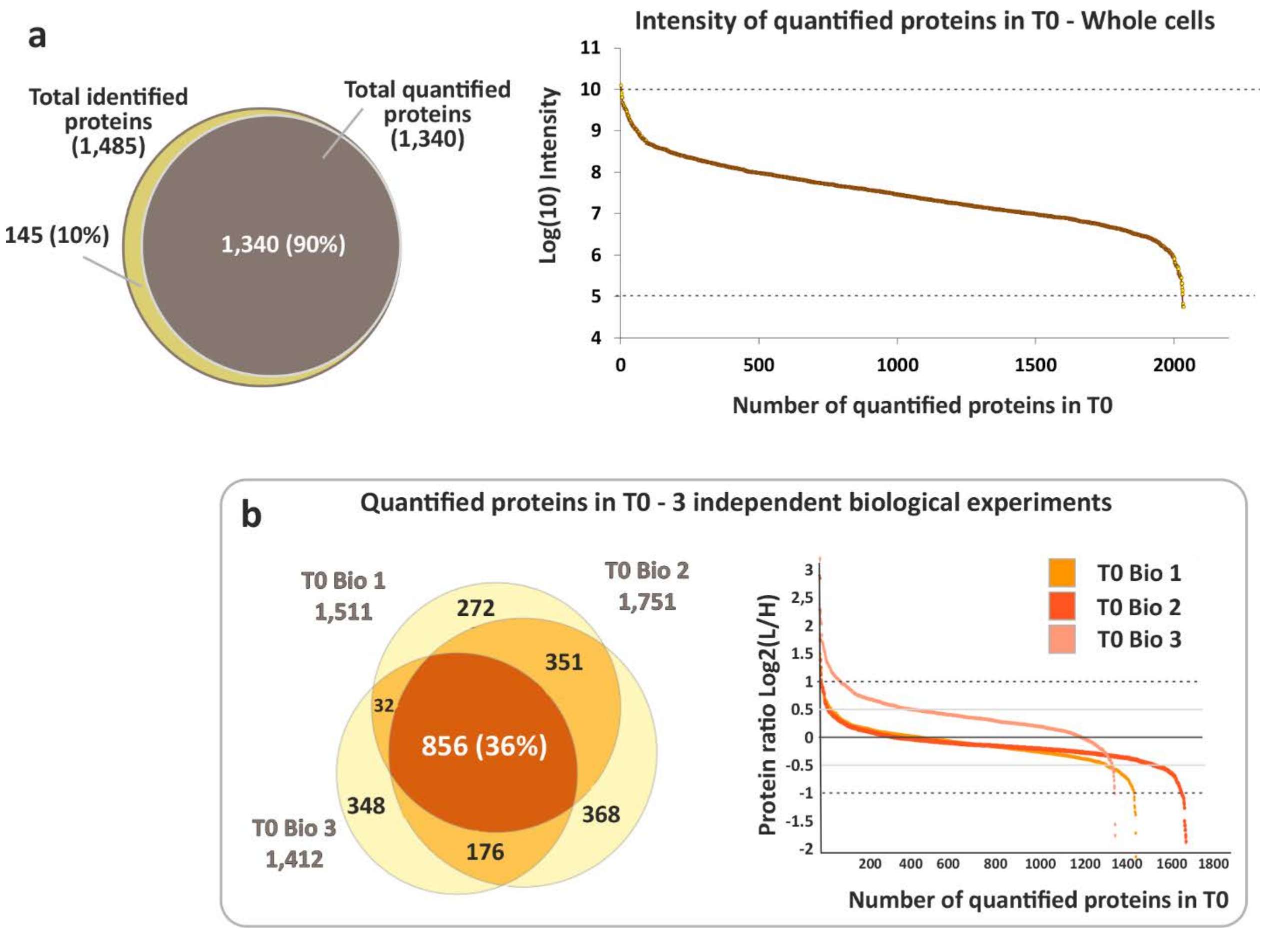

Figure S.1.2 Validation of the SILAC experiment: mass spectrometry. a Total number of identified and quantified proteins of whole HT29-MD2 cells in T0, and the dynamic range of quantified protein abundance. Data correspond to three combined independent biological experiments analyzed in technical triplicates in LC-MS/MS. **b** Distribution of quantified proteins in T0 sample in three biological experiments by a Venn diagram and the distribution of log2 intensities of quantified proteins of each triplicate.

Table S.1.1 The Up- and down-leveled proteins in LPS, LPS **Mn1,** and **Mn1** whole cell samples, classified according to their associated biological processes, using a combination of bioinformatic tools (DAVID, STRING, Perseus, UniProt) and validation from literature. In red bold underlined are highlighted the most impacted proteins, and in blue bold italics the proteins seen in all conditions (6-h LPS, 6-h LPS **Mn1**, and 6-h **Mn1**).

T6h LPS (Up) [55 proteins]	T6h LPS Mn1 (Up) [16 proteins]	T6h Mn1 (Up) [20 proteins]	T6h LPS (Down) [57 proteins]	T6h LPS Mn1 (Down) [15 proteins]	T6h Mn1 (Down) [16 proteins]
RNA processing, Transcription, Translation					
HIST2H2AC [x1.4]	***HIST2H2AC [x1.2]***	***HIST2H2AC [x1.3]***	**GARS [x0.6]**	SF3B2 [x0.6]	HNRNPH3 [x0.8]
SRSF3 [x1.4]	YBX1 [x1.2]	EIF3F [x1.3]	**AARS [x0.6]**	SERBP1 [x0.8]	DDX17 [x0.8]
RBM12 [x1.3]	BANF1 [x1.2]	SF3A1 [x1.2]	**RPRD1B [x0.6]**	LUC7L2 [x0.8]	SNRPA1 [x0.9]
EIF1 [x1.2]	RBM3 [x1.1]	TRIM28 [x1.2]	RPL35A [x0.7]	DDX17 [x0.9]	HNRNPUL2 [x0.9]
TARDBP [x1.2]	EIF4H [x1.1]	RPS6 [x1.1]	RPL28 [x0.8]		HNRNPA0 [x0.9]
HNRNPH2 [x1.2]		HNRNPH2 [x1.1]	SF3A3 [x0.8]		SF3B2 [x0.9]
RPL18 [1.2]		U2AF2 [x1.1]	GTF2I [x0.8]		SRSF7 [x0.9]
DEK [1.2]		TOP1 [x1.1]	METAP2 [x0.8]		PSPC1 [x0.9]
NHP2L1 [1.2]		ACIN1 [x1.1]	TSN [x0.8]		
PTRHD1 [1.2]		H2AFY [x1.1]	ZC3H4 [x0.8]		
DOHH [1.2]			ZC3H15 [x0.8]		
RCC2 [1.2]			TBR1 [x0.8]		
			ZNF185 [x0.8]		
			HDGFRP2 [x0.8]		
			PDDC1 [x0.8]		
			TSNAX [x0.8]		
			MAGOH [x0.8]		

**Mitochondrial proteins**					
**ATP5C1 [x2.4]**<br>**TOMM22 [x1.7]**<br>PDHA1 [x1.4]		DLST [x1.2]	**PGAM [x0.6]**<br>CYB5A [x0.8]		CS [x0.9]<br>STOML2 [x0.9]
**Innate immunity response**					
**SDCBP [x1.7]**<br>B2M [x1.4]<br>HLA-A-36 [x1.4]<br>HLA-A-gnas24 [x1.2]	FKBP1A [x1.3]		SERPINB1 [x0.7]<br>FKBP3 [x0.8]		
**Antioxidant**					
TXNRD1 [x1.2]		ESD [x1.1]	NQO1 [x0.8]		
**Metabolic processes**					
PSPH [x1.3]<br>GOT1 [x1.3]<br>NAMPT [x1.2]<br>UGT1A10 [x1.2]<br>MAT2A [x1.2]			ATIC [x0.8]<br>PNP [x0.8]<br>HPRT1 [x0.8]<br>APMAP [x0.8]<br>UGP2 [x0.8]<br>EPHX1 [x0.8]<br>PFKP [x0.8]<br>FAM3C [x0.8]		
**Cytoskeleton organization / Cell adhesion / Epithelial cell polarity / ECM reorganization**					
LAD1 [x1.4]<br>CTNND1 [x1.3]<br>PODXL [x1.3]<br>ARPC4 [x1.2]<br>BAIAP2L1 [x1.2]	SERPINH1 [x1.2]<br>ARPC2 [x1.2]	SERPINH1 [x1.3]<br>RTN4 [x1.1]<br>LDHB [x1.1]<br>WDR1 [x1.1]	***ANXA11 [x0.7]***<br>***EPS8L1 [x0.7]***<br>***TACSTD2 [x0.7]***<br>CDC42EP4 [x0.8]<br>KRT80 [x0.8]<br>FBL [x0.8]	***ANXA11 [x0.8]***<br>***EPS8L1 [x0.8]***<br>STMN1 [x0.8]<br>TFG [x0.8]<br>***TACSTD2 [x0.9]***<br>ITGA6 [x0.9]<br>RTN4 [x0.9]	***ANXA11 [x0.8]***<br>ITGA6 [x0.9
**Protein degradation**					
PCMT1 [x1.4]<br>PSMC6 [x1.2]<br>PSMA6 [x1.2]	OTUB1 [x1.3]<br>UBE2N [x1.2]<br>PSMA1 [x1.2]	OTUB1 [x1.5]<br>PPT1 [x1.3]	**KLK6 [x0.6]**<br>USP14 [x0.7]<br>SUMO4 [x0.8]<br>PSMB7 [x0.8]	UBXN1 [0.8]<br>PSMC3 [x0.9]	
**Transport / Vesicle endocytosis**					
**SDF4 [x1.6]**<br>ATP1B1 [x1.4]<br>ARL6IP5 [x1.3]<br>CLIC4 [x1.3]<br>LAMP1 [x1.3]<br>RAB2A [x1.3]<br>SAR1A [x1.2]<br>PDCD6 [x1.2]<br>OSBP [x1.2]	CALB2 [x1.2]		**IPO9 [x0.6]**<br>TNPO1 [x0.7]<br>SLC2A1 [x0.7]<br>AP1B1 [x0.8]<br>CHP1 [x0.8]<br>CRABP2 [x0.8]<br>ATXN2L [x0.8]<br>TMED2 [x0.8]<br>ARF4 [x0.8]<br>KPNA2 [x0.8]		CRABP2 [x0.9]
**Signaling**					
**ARHGEF1 [x2.2]**<br>GNAS [x1.4]<br>***YWHAQ [x1.2]***	***YWHAQ [x1.2]***<br>SH3KBP1 [x1.1]	YWHAQ [x1.2]	**KRT1 [x0.6]**<br>GNB2L1 [x0.8]	CRK [x0.9]	
**Chaperones / stress response**					
LZIC [x1.3]<br>GNAI2 [x1.2]<br>SLC3A2 [x1.2]<br>CNPY2 [x1.2]<br>SSR3 [x1.2]<br>SSR2 [x1.2]<br>SSR1 [x1.2]<br>RAP1A [x1.2]<br>RHOG [x1.2]<br>NOSIP [x1.2]	DNAJB1 [x1.2]<br>HSPH1 [x1.2]		***DNAJA2 [x0.7]***<br>PDIA4 [x0.8]<br>HSPBP1 [x0.8]	***DNAJA2 [x0.8]***	DNAJA1 [x0.9]<br>VBP1 [x0.9]
**Apoptosis**					
		TGM2 [x1.1]	STK24 [x0.8]<br>AIFM1 [x0.8]		EFHD2 [x0.9]

B. OcSILAC (Oc: Oxidized cysteine) validation in five subcellular fractions

SILAC proteomics in five subcellular fractions provided better coverage of the proteome (Fig S.1.3a). Considering that whole cell protein quantification decreases the signal-to-noise ratio for proteins specific to a particular subcellular compartment and compresses the ratios for proteins with localized variations, we combined the proteomic experiment with subcellular fractionation, known to improve proteome analysis depth. The subcellular fractionation study was validated by identifying specific protein markers in the expected subcellular fractions (Fig S.1.3b). As expected, many proteins including SOD2, were identified exclusively in the samples of the membrane-rich fraction containing organelles. The subcellular fractions increased the number of identified proteins (data not shown). It also provided an enrichment of mitochondrial (inner/outer membrane, matrix proteins, ETC components, ribosomal subunits), endoplasmic, and Golgi proteins (Fig. S.1.4). The two more abundant subcellular fractions were the cytosolic and plasma membrane & organelles (PMO) fractions on which we focused the rest of the study.

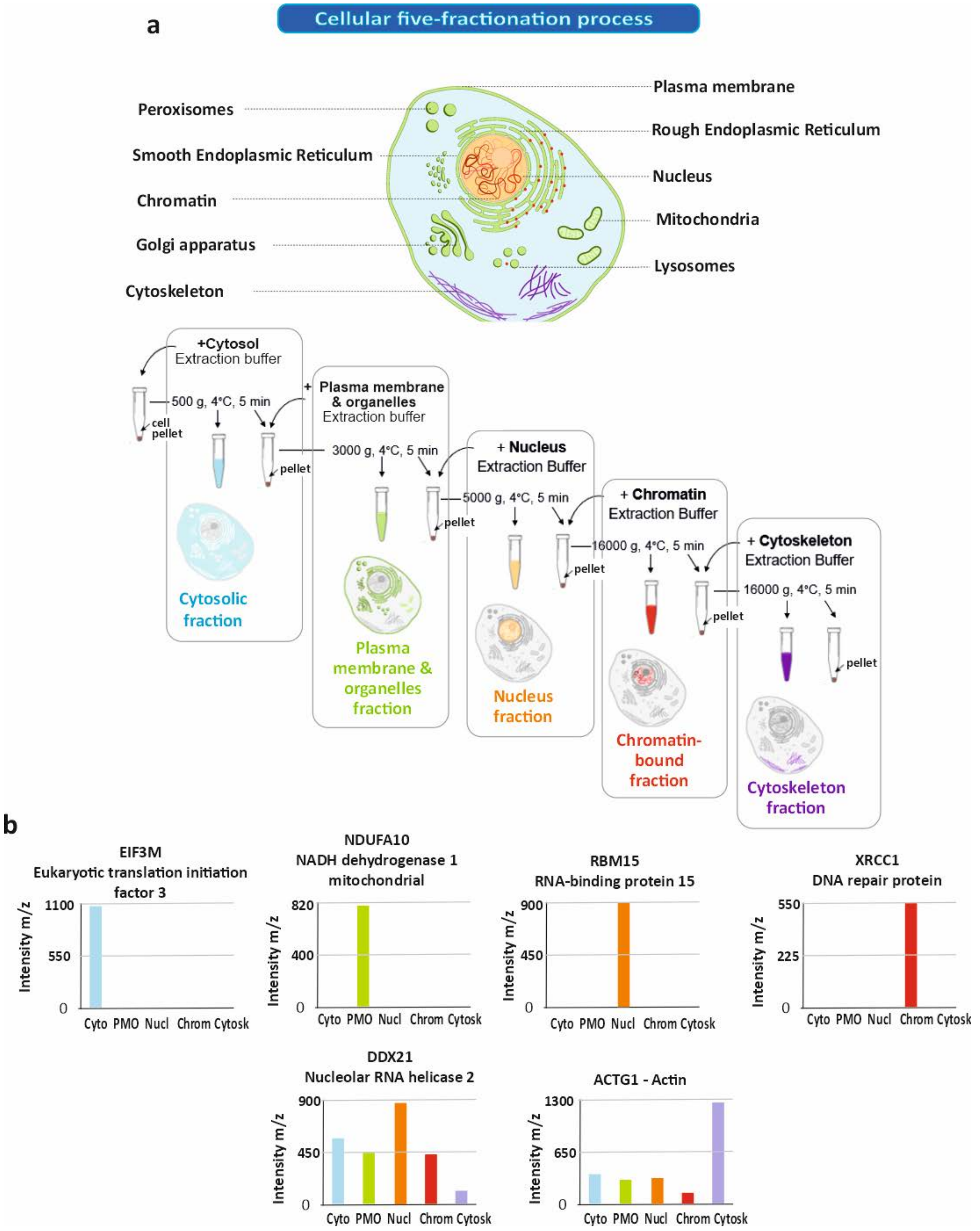

Figure S.1.3 Validation of the subcellular fractionation a Subcellular protein fractionation workflow**.** Stepwise lysis of mammalian cells was performed by using different extraction buffers to separate proteins in five separate fractions coming from the cytosol (in blue), the plasma membrane & organelles, like mitochondria, Golgi, endoplasmic reticulum, lysosomes, peroxisomes, etc. (in green), the nucleus (in orange), the chromatin-bound proteins (in red), and the cytoskeleton (in purple). The first kit reagent, when added to a cell pellet, causes selective membrane permeabilization, releasing the soluble cytosolic content. The second reagent dissolves the plasma membrane, mitochondria, and ER-Golgi membranes but does not solubilize the nuclear membranes. The fraction that was extracted in each step is colored respectively. **b** Fractionation efficiency of the OcSILAC adapted fractionation protocol in HT19-MD2 cells. Heavy cells (10^6, non-treated) were mixed with light cells (10^6, non-treated) from the T0 sample, fractionated by following the OcSILAC adapted fractionation protocol. Peak intensities obtained for six proteins

(whose specific compartmentalization is well established in the literature) in the different fractions are highlighted. EIF3M, a translation initiator factor, with predicted subcellular location by UniProt the cytosol, was detected only in the cytosolic fraction (Cyto); the mitochondrial NDUFA10 dehydrogenase was identified only in the plasma membrane and organelles fraction (PMO); RBM15, acting in the methylation of RNA in the nucleus, was identified only in the nucleus fraction (Nucl); XRCC1, involved in DNA single-strand break repair by mediating the assembly of DNA break repair protein complexes, was identified in the chromatin-bound fraction (Chrom); and ACTG1, actin, was mainly identified in the cytoskeleton fraction (Cytosk), and with a lesser extent in the other fractions. However, proteins such as DDX21, located in different subcellular compartments were identified in different fractions with equal intensities.

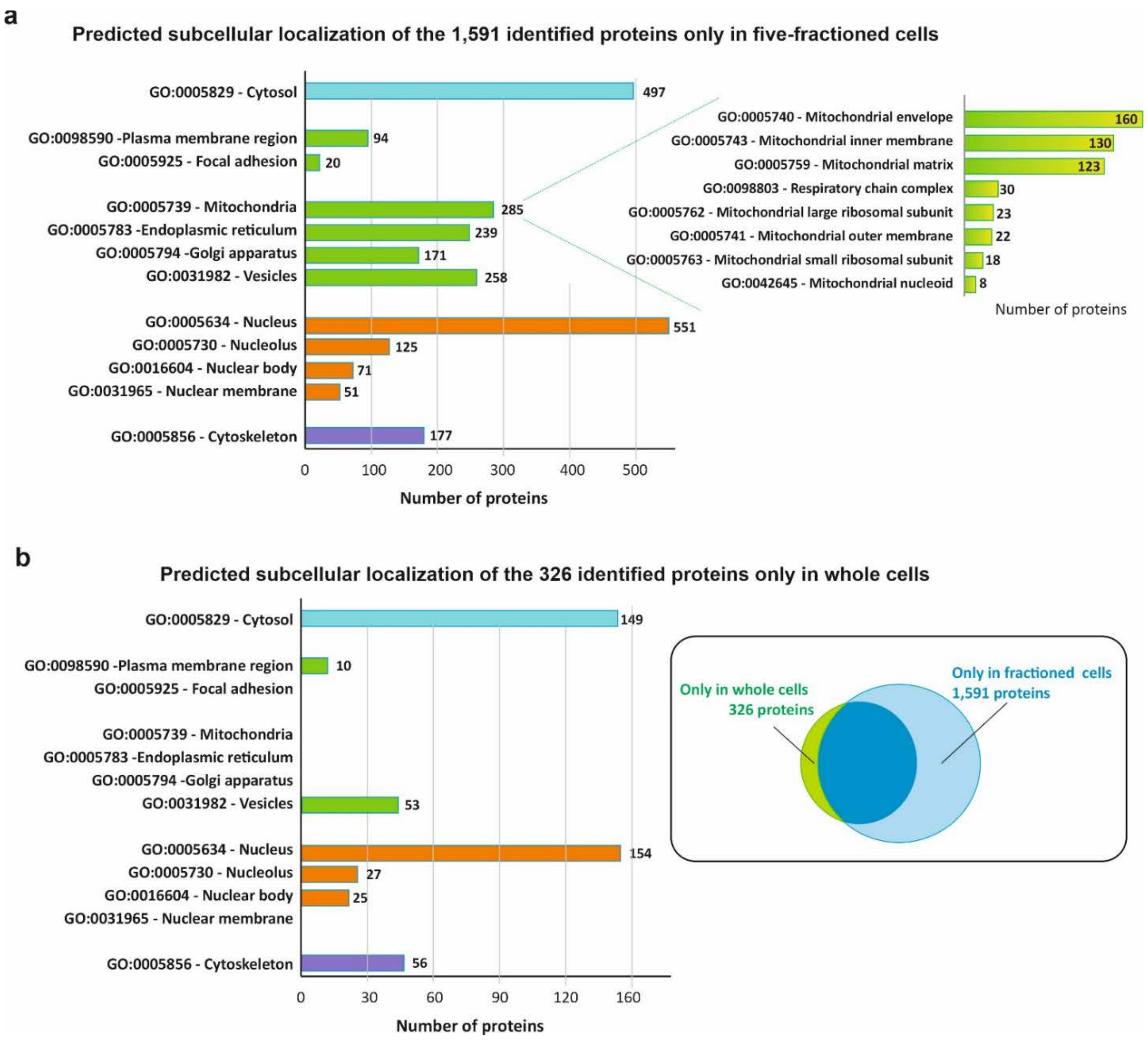

Figure S.1.4 Gene ontology cellular compartment enrichment on proteins specific to whole cells and fractioned cells. a. To qualify the 5-fraction contribution, we classified the proteins of the T0 sample that were specific to fractioned cells (1,591) –non-identified in whole cells- according to their predicted subcellular localization; these proteins were mostly cytosolic (497) and/or nuclear (551), but with an important number of mitochondrial (285), ER (239) and Golgi (171) proteins. b. The mitochondrial proteins belong to the inner or outer membranes and the matrix, and they are components of the ETC complex or the ribosomic subunits. c. Proteins specific to whole cells (326) –non-identified in the fractioned cells- were mostly cytosolic (149) and/or nuclear (154) with a small number of cytoskeletal (56) and vesicular (53) proteins. Gene ontology cellular compartment enrichment was done with STRING bioinformatic tool v.11.[8–10]

C. OcSILAC final experiment in two subcellular fractions

Gel imaging to evaluate free thiol blockage efficiency

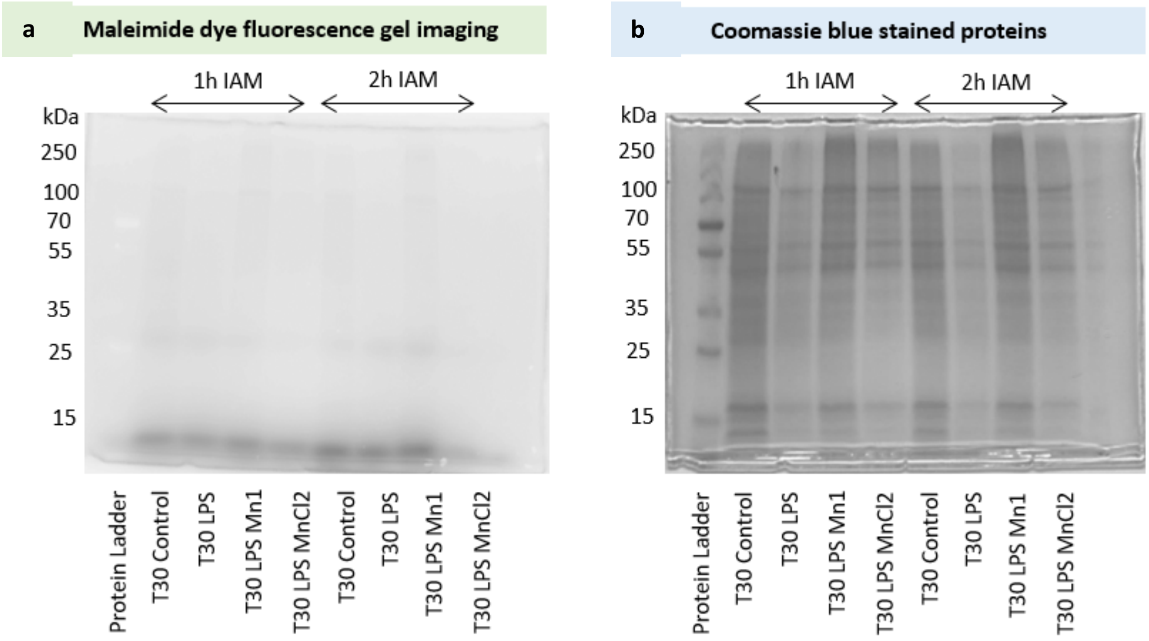

Figure S.1.5 Set-up of the OcSILAC conditions and workflow in whole cells. HT29-MD2 light cells were incubated for 30 min with cell media, LPS, LPS **Mn1**, LPS $MnCl_2$ in the described conditions. The blockage efficiency of free thiol groups (originally reduced cysteines) with iodoacetamide (IAM) was evaluated by quantifying the fluorescence after incubation with the maleimide functionalized fluorophore that strongly reacts with free thiols. Proteins were extracted and alkylated by IAM (200 mM) in denaturing buffer at 37 ºC for 1-2 h. The maleimide functionalized fluorophore DyLight 448 was added for 1 h at 37 °C. SDS-page gel electrophoresis was performed in Tris-Glycine-SDS (1x) running buffer for 1 h at 25 mA (same amount of sample loaded in each well). **a** Fluorescence imaging in blue light (460 nm). DyLight 448 dye excitation max at 493 nm and emission max at 518 nm. **b** Colorimetric imaging after 2 h Coomassie blue staining and five washes with Milli-Q water. An absence of fluorescence in the gel (a) indicates that all the free cysteines were efficiently blocked by IAM and could not react further with the maleimide moiety. Coomassie blue staining (gel b) of the total proteins present in the gel confirmed that the absence of fluorescence was due to efficient IAM alkylation and not to a lack of proteins loaded on the gel.

In the combined datasets of all conditions, 83 % (3,327) of the 4,004 identified proteins were quantified, as expected,[2,3,11] The dynamic range of the quantified proteins in the T0 sample at was distributed over six magnitude orders (Fig. S.1.6a) suggesting a better coverage of the proteome compared to the dynamic range detected with whole cell protein extracts (four magnitude orders, see Fig. S.1.2a). Despite the observation that some proteins were specific to each fraction (0.30-0.47 Pearson correlation comparing the two fractions at 6 h LPS), a 0.85 Pearson correlation was observed between the biological triplicates of cytosolic and PMO fractions (scatter plots, Fig. S.1.7).

The quantification reproducibility of the experiments was evaluated in the three independent biological experiments for the T0 sample. A total of 3,068 proteins were identified, and 2,033 (66 %) proteins were quantified in the combined results from the cytosolic and plasma membrane & organelles fractions, with 45 % of quantified proteins in all three biological replicates and 75 % in at least two replicates (Fig. S.1.6b). Distribution of the quantified protein ratios (L/H) showed that over 90 % of the dynamic range was centered around one in three biological replicates, meaning that L=H as expected. The reproducibility between the three replicates was evaluated with a 0.6-0.8 Pearson coefficient between the biological T0 triplicates (scatter plot representations, Fig. S.1.6c).

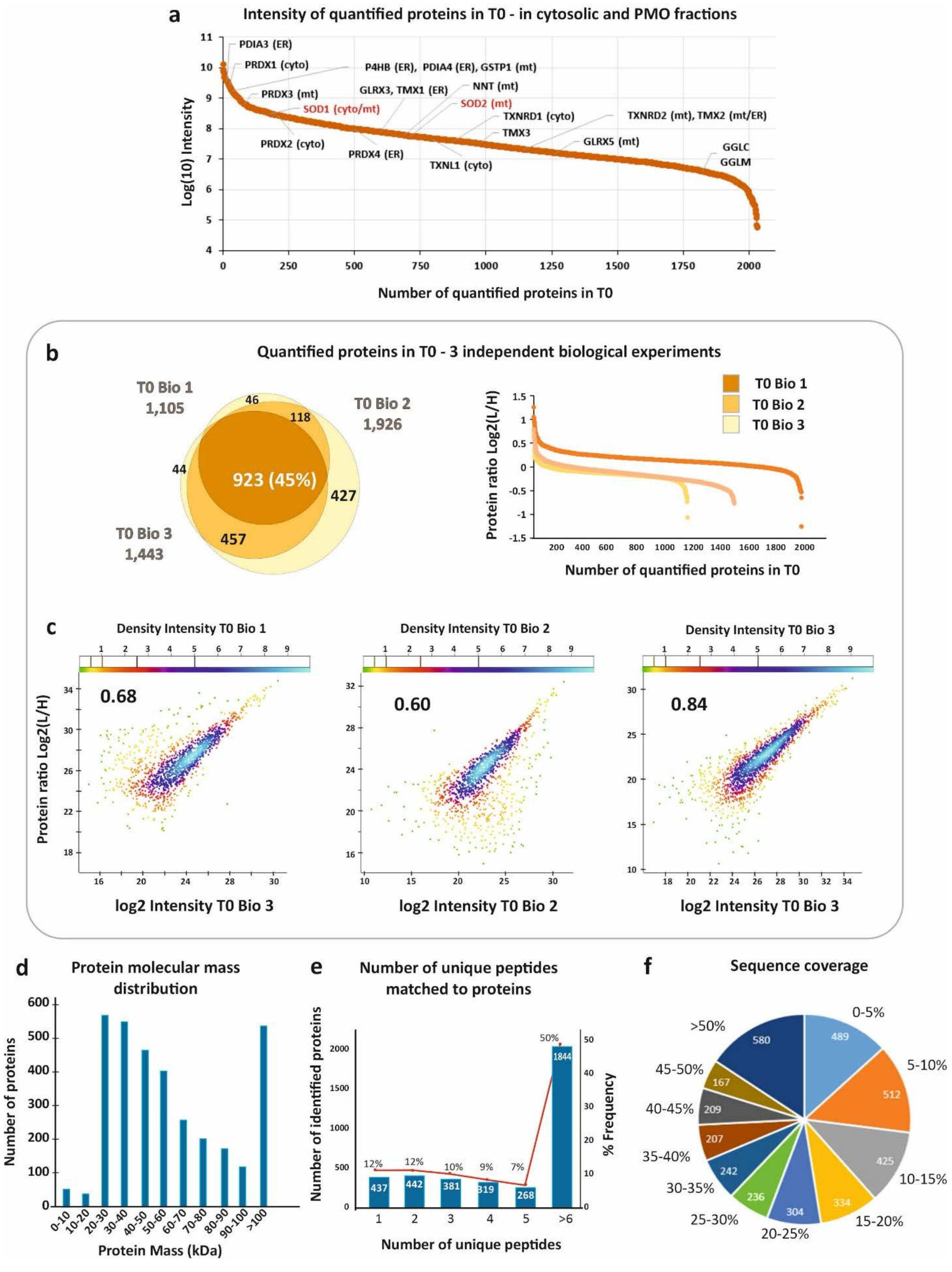

Figure S.1.6 Proteomics metadata a Dynamic range of quantified protein abundance of the two-fractioned HT29-MD2 cells proteome, in T0 samples - distribution of log10 intensities of quantified proteins. **b** Coverage of quantified proteins in three biological replicates (T0) in a Venn diagram, and the distribution of quantification ratios L/H of each triplicate (log 2 scale). **c** Density multi-scatter plot of the protein intensities for the three replicates plotted against each other (log2 scale). Pearson correlation is shown in the top left corner. Data correspond to three independent biological experiments analyzed in technical triplicates in LC-MS/MS. **d** Protein molecular mass (kDa) distribution: most of the identified proteins in T0 had molecular weights in the range of 20–60 kDa and >100 kDa. **e** Histogram displaying the

number of peptides matched to proteins. The x-axis illustrates the number of the identified peptides that are unique to a protein. The primary y-axis indicates the number of identified proteins (bars). The second y-axis represents the percent (lines). 76 % of proteins identified with more than 2 unique peptides. **f** Sequence coverage % of identified proteins.

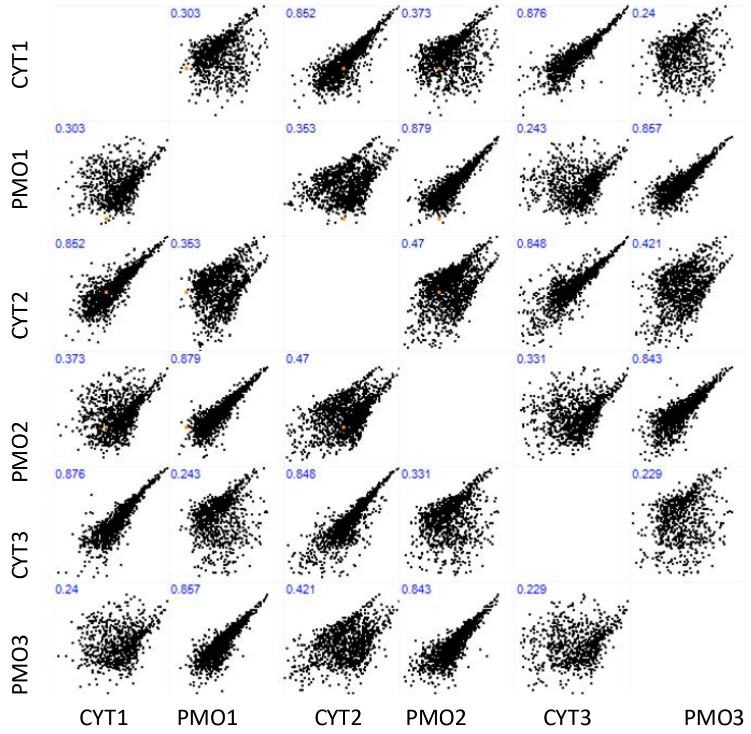

Figure S.1.7 Reproducibility and population differentiation by scatter plot representation. Intensities of proteins quantified in the PMO and cytosolic fraction plotted in log2. Proteins, represented by distinct points in the scatter plot, are compared between two biological replicates of HT29-MD2 cell samples after 6 hour LPS-stimulation, subcellular fractionation, OcSILAC, and LC-MS/MS analysis. The two fractions in the same or different biological replicate comprise two distinct populations, with proteins specific to each fraction. A positive correlation is observed between each fraction in biological duplicates, confirming the reproducibility of these proteomic experiments analyzed samples. The scatter plot was done in the Perseus software (version 1.6.10.43).

Bibliography SI1

1. Kirchner, M. & Selbach, M. In Vivo Quantitative Proteome Profiling: Planning and Evaluation of SILAC Experiments. in *Quantitative Methods in Proteomics* (ed. Marcus, K.) 175–199 (Humana Press, Totowa, NJ, 2012). doi:10.1007/978-1-61779-885-6_13.
2. Bantscheff, M., Schirle, M., Sweetman, G., Rick, J. M. & Kuster, B. Quantitative mass spectrometry in proteomics: a critical review. *Analytical and bioanalytical chemistry* (2007) doi:10.1007/S00216-007-1486-6.
3. Do, M. *et al.* Quantitative proteomic analysis of pancreatic cyst fluid proteins associated with malignancy in intraductal papillary mucinous neoplasms. *Clin Proteom* **15**, 17 (2018).
4. Nøhr, M. K. *et al.* SILAC-MS Based Characterization of LPS and Resveratrol Induced Changes in Adipocyte Proteomics – Resveratrol as Ameliorating Factor on LPS Induced Changes. *PLoS ONE* **11**, e0159747 (2016).

5. Itzhak, D. N. *et al.* SILAC-based quantitative proteomics using mass spectrometry quantifies endoplasmic reticulum stress in whole HeLa cells. *Dis. Model. Mech.* **12**, dmm040741 (2019).

6. Deng, J., Erdjument-Bromage, H. & Neubert, T. A. Quantitative Comparison of Proteomes Using SILAC. *Current Protocols in Protein Science* **95**, e74 (2019).

7. Xie, L.-Q. *et al.* Global in vivo terminal amino acid labeling for exploring differential expressed proteins induced by dialyzed serum cultivation. *Analyst* **139**, 4497–4504 (2014).

8. Szklarczyk, D. *et al.* STRING v11: protein–protein association networks with increased coverage, supporting functional discovery in genome-wide experimental datasets. *Nucleic Acids Research* **47**, D607–D613 (2019).

9. Szklarczyk, D. *et al.* The STRING database in 2017: quality-controlled protein–protein association networks, made broadly accessible. *Nucleic Acids Res* **45**, D362–D368 (2017).

10. Szklarczyk, D. *et al.* STRING v10: protein–protein interaction networks, integrated over the tree of life. *Nucleic Acids Research* **43**, D447–D452 (2015).

11. Liu, Z. *et al.* iTRAQ-based quantitative proteomic analysis of salt stress in Spica Prunellae. *Sci Rep* **9**, 9590 (2019).

Supplementary Information 2

Analyses of the Proteomic and Redoxomic Data

Data processing with volcano plot representation

Several of the quantified proteins have levels impacted by the incubations (LPS, LPS **Mn1**, etc.). To know which level variations are significant between a condition of interest and the basal cell state in various replicates, fold change (variation between the two conditions) and the variation within replicates of each condition were taken into account. In this study, Perseus statistics software (version 1.6.10.43) was used to determine the statistically significant level variation in every condition, and illustrated by volcano plots.

A permutation-based false discovery rate (FDR) threshold was set to 0.05 (5% error), which corresponds to a calculated experimental p-value (after 250 randomizations between the treated and basal conditions). The list of identified species was filtered according to this value. A modified t-test was used in the Perseus algorithm using the significance analysis of microarrays (SAM) approach, with a parameter that considers the fold-change. Therefore, rather than using a horizontal cutoff (considering statistical significance only) or a vertical cutoff (considering fold change only), the significant values are defined by a curve superimposed on the volcano plot. Every value above the volcano plot threshold curve is significant, with the most significant changes being the most far away from the threshold curve.

For all the figures, LPS, LPS **Mn1**, **LPS $MnCl_2$, and Mn1** effects were plotted in volcano plots giving the t-test p-value (statistical significance of the measurement in log10 scale y-axis; significant if p-value < 0.01, 1 % error) against the fold-change between two chosen conditions (in log2 scale x-axis). The further away from zero on the x-axis (fold change of a condition of interest vs. T0 in log2), the more significant the variation. The higher the value on the y-axis (statistical significance in –logP value), the more robust the data. The overexpression appears with positive log2 values and the under-expression with negative log2 values.

A. 6 hours

LPS, LPS Mn1, Mn1 alone and LPS $MnCl_2$ effects on the proteome level and Cys oxidation level, after 6-h incubation

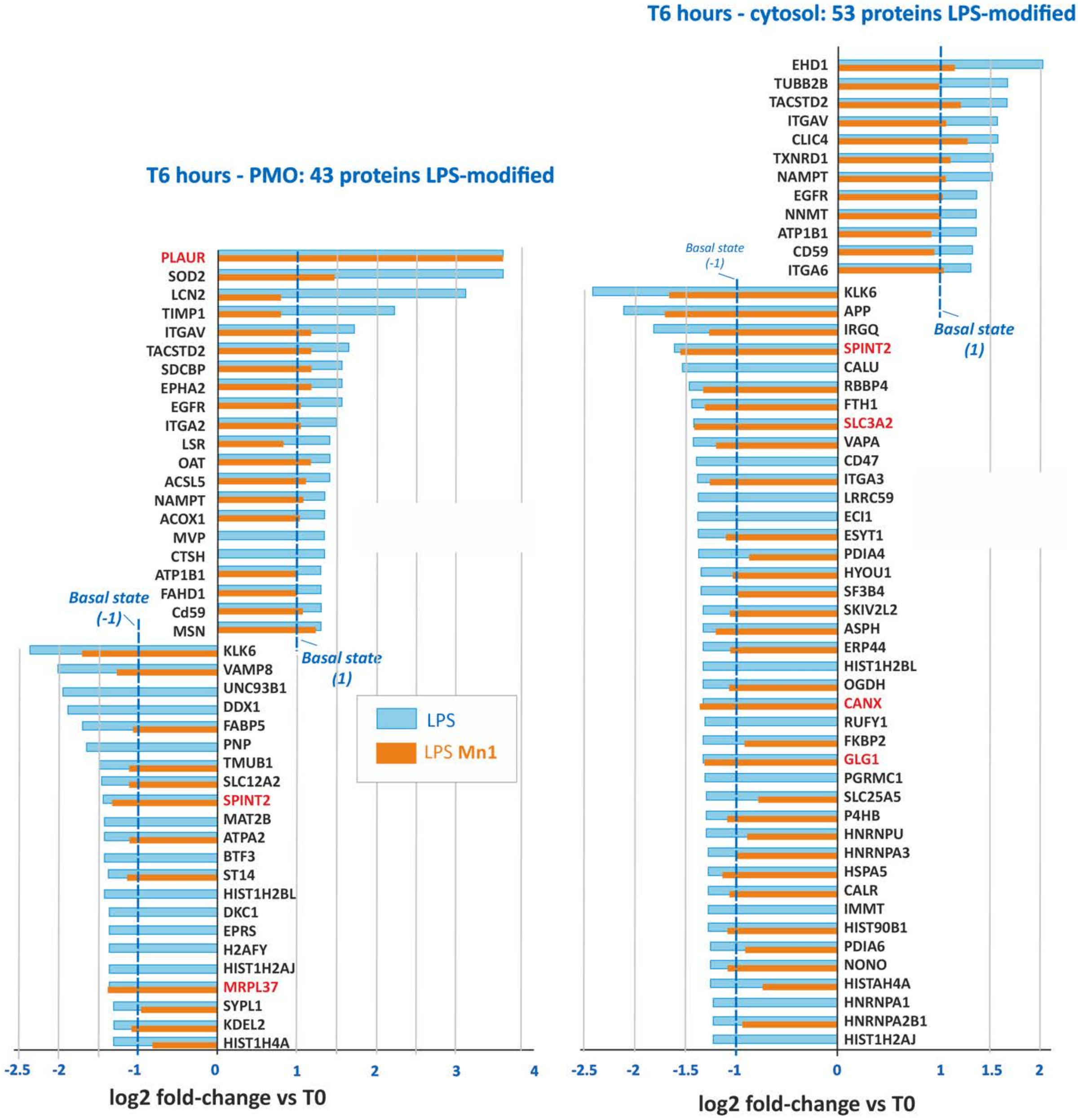

Figure S.2.1 Comparison of the effects of LPS and LPS Mn1 6-h incubation vs. T0 sample (non incubated or basal sample) on the protein levels. After 6 hours incubation, from the 43 PMO LPS up- or down-leveled proteins, only 31 were quantified in the LPS **Mn1** samples. In the presence of **Mn1,** 11 proteins were found at their basal level, 17 protein variations were attenuated by 10-70 %, and three proteins were not affected (PLAUR, MRPL37, SPINT2). Concerning the cytosolic fraction, from the 53 LPS up- or down-leveled proteins, 43 were quantified in LPS **Mn1**. In the presence of **Mn1**, 19 proteins were found at their basal level, 21 protein variations were attenuated by 10-90 %, and three proteins were not affected (GLG1, CANX, SLC3A2). The LPS condition is shown in blue and the LPS **Mn1** condition is shown in orange.

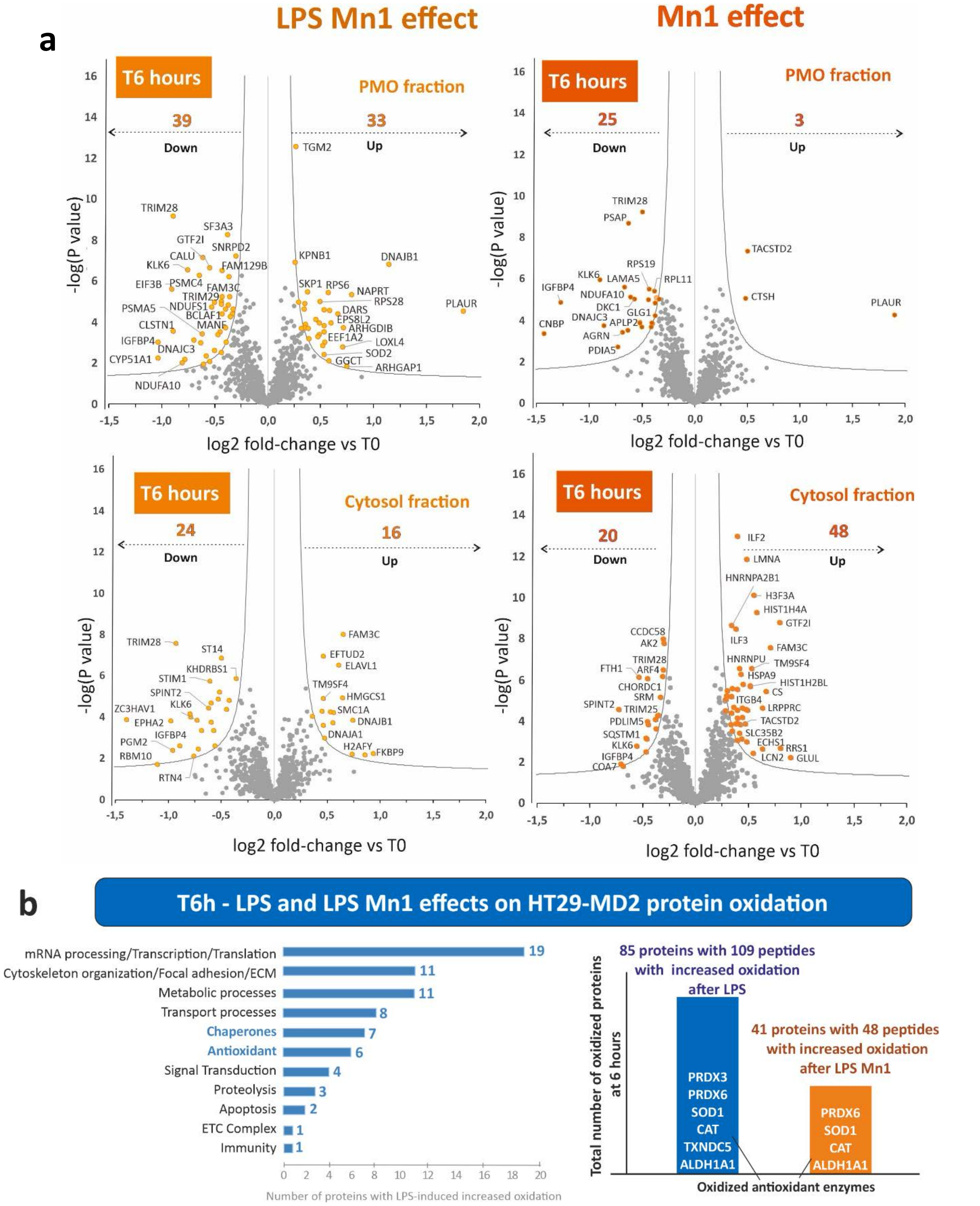

Figure S.2.2 Comparison of LPS Mn1 and Mn1, 6-h incubation. a Effects on protein levels of a 6-h incubation sample **with LPS Mn1, and Mn1 (**vs. T0 sample) in the PMO and cytosolic fractions of HT29-MD2 cells. LPS **Mn1,** and **Mn1** effects were plotted in volcano plots giving the t-test p-value (in log10 scale y-axis; significant if p-value < 0.01, 1 % error) against the fold-change between two chosen conditions (in log2 scale x-axis). The up- and down-leveled proteins in LPS **Mn1** are indicated with orange circles, and in **Mn1 alone they** are indicated with dark orange circles. The LPS **Mn1** condition is shown in orange and the **Mn1** condition is shown in dark orange. **b** LPS and LPS **Mn1** effects on protein oxidation status in both PMO and cytosolic fractions. Left: LPS-oxidized proteins reported according to their biological function, right: LPS-oxidized proteins are in the LPS **Mn1** sample. The LPS condition is shown in blue, and the LPS **Mn1** condition is shown in orange.

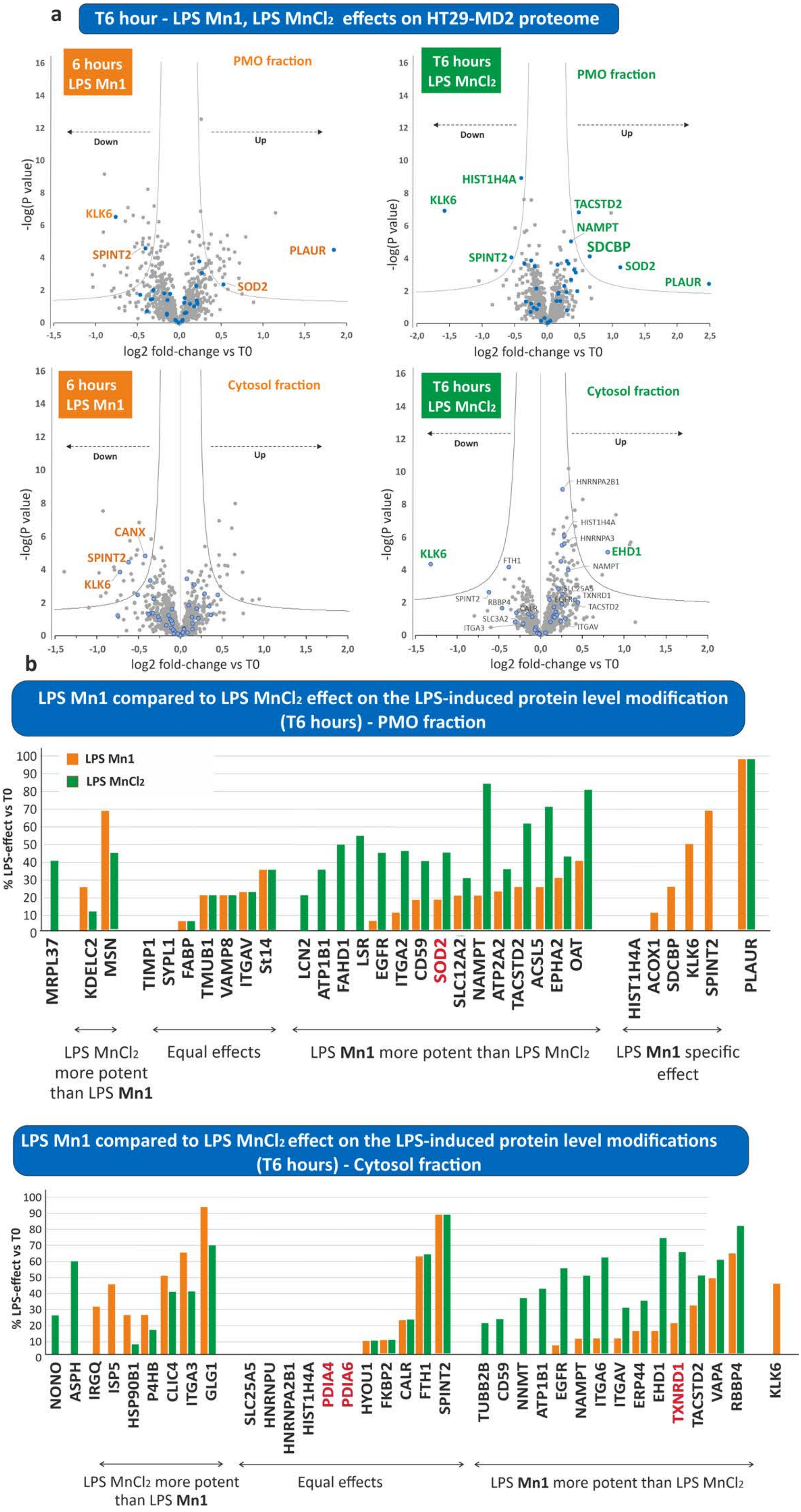
a
T6 hour - LPS Mn1, LPS MnCl2 effects on HT29-MD2 proteome
6 hours LPS Mn1
PMO fraction
Down
Up
KLK6
SPINT2
PLAUR
SOD2
-log(P value)
log2 fold-change vs T0
T6 hours LPS MnCl2
PMO fraction
HIST1H4A
KLK6
TACSTD2
NAMPT
SDCBP
SPINT2
SOD2
PLAUR
6 hours LPS Mn1
Cytosol fraction
CANX
SPINT2
KLK6
T6 hours LPS MnCl2
Cytosol fraction
HNRNPA2B1
HIST1H4A
HNRNPA3
EHD1
NAMPT
FTH1
KLK6
SLC25A5
TXNRD1
TACSTD2
SPINT2
RBBP4
EGFR
CALR
SLC3A2
ITGA3
ITGAV
b
LPS Mn1 compared to LPS MnCl2 effect on the LPS-induced protein level modification (T6 hours) - PMO fraction
LPS Mn1
LPS MnCl2
% LPS-effect vs T0
MRPL37
KDELC2
MSN
TIMP1
SYPL1
FABP
TMUB1
VAMP8
ITGAV
St14
LCN2
ATP1B1
FAHD1
LSR
EGFR
ITGA2
CD59
SOD2
SLC12A2
NAMPT
ATP2A2
TACSTD2
ACSL5
EPHA2
OAT
HIST1H4A
ACOX1
SDCBP
KLK6
SPINT2
PLAUR
LPS MnCl2 more potent than LPS Mn1
Equal effects
LPS Mn1 more potent than LPS MnCl2
LPS Mn1 specific effect
LPS Mn1 compared to LPS MnCl2 effect on the LPS-induced protein level modifications (T6 hours) - Cytosol fraction
% LPS-effect vs T0
NONO
ASPH
IRGQ
ISP5
HSP90B1
P4HB
CLIC4
ITGA3
GLG1
SLC25A5
HNRNPU
HNRNPA2B1
HIST1H4A
PDIA4
PDIA6
HYOU1
FKBP2
CALR
FTH1
SPINT2
TUBB2B
CD59
NNMT
ATP1B1
EGFR
NAMPT
ITGA6
ITGAV
ERP44
EHD1
TXNRD1
TACSTD2
VAPA
RBBP4
KLK6
LPS MnCl2 more potent than LPS Mn1
Equal effects
LPS Mn1 more potent than LPS MnCl2

Figure S.2.3 Comparison of LPS Mn1 and $MnCl_2$, 6-h icubation. Effects of LPS Mn1 and LPS $MnCl_2$ on LPS-induced protein levels after 6 h-incubation vs. T0 sample. a LPS **Mn1** and **LPS $MnCl_2$** effects were plotted in volcano plots giving the t-test p-value (log10 scale y-axis; significant if p-value < 0.01, 1 % error) against the fold-change between two chosen conditions (in log2 scale x-axis). The LPS up-leveled proteins have been underlined with blue circles. In LPS **Mn1** samples, most of the previously LPS up-leveled proteins were returned to their basal levels as illustrated by the displacement of the blue circles. (up- and down-leveled proteins after 6 h LPS or LPS Mn1 underlined with blue circles). **b** Comparison of the effects on LPS **Mn1** with those of LPS $MnCl_2$, in preventing/attenuating the LPS-induced protein level modification, in PMO and cytosolic fractions 0 % means that LPS $MnCl_2$ or LPS **Mn1** totally prevent the LPS-induced modification of the protein level, although, 100 % means that LPS $MnCl_2$ or LPS **Mn1** do not affect the LPS effect. For example, for the TIMP1, SYPL1, HIST1H4A (in the PMO fraction) and SLC25A5, HNRNPU, HNRNPA2B1, HIST1H4A, PDIA4, and PDIA6 (in the cytosolic fraction) 0 % means that LPS $MnCl_2$ and LPS **Mn1** totally prevent their LPS-induced modification. The LPS **Mn1** condition is shown in orange and the LPS $MnCl_2$ condition is shown in green.

Table S.2.1 Up- and down-leveled proteins after 6-h LPS-stimulation of HT29-MD2 cells vs.T0 (non-incubated or basal sample). The headline of the LPS condition is shown in blue. Down-leveled proteins are highlighted in yellow.

Protein	Protein name	Fold-change	Log2 difference	-Log (P value)
**T6 hours LPS-modified proteins, in PMO**				
SOD2	Superoxide dismutase [Mn], mitochondrial	3.6	1.8	7.5
PLAUR	Urokinase plasminogen activator surface receptor<br>*GPI-anchored PLAUR is the receptor for the extracellular serine protease urokinase-type plasminogen activator. Though PLAUR role in inflammatory processes is documented,[39] underlying mechanisms are not fully understood. PLAUR is a part of TLR4, and its downregulation resulted in diminished LPS-induced TLR4 signaling, less activation of NFκB, and decreased secretion of inflammatory mediators in myeloid and non-myeloid cells in vitro.[40]*	3.6	1.8	3.0
LCN2	Neutrophil gelatinase-associated lipocalin<br>*The siderophore is known as the first-line protection against microbial invasion.*	3.1	1.6	4.0
TIMP1	Metalloproteinase inhibitor 1<br>*It inhibits the metalloproteases hydrolyzing ECM components, leading to accumulation of the ECM (TIMPs>MMPs), causing tissue fibrosis.[41] Its expression was found to increase after LPS in vivo and in vitro; an increase that peaked at 12h, recovered at 24h, and caused limiting tissue damage.[42]*	2.2	1.1	2.3
ITGAV<br>ITGA2	Integrin alpha-V<br>Integrin alpha-2<br>*Structural and functional bridges between ECM and actin cytoskeleton, play an important role in cell adhesion and motility, but also modulating cell survival, proliferation, and differentiation.[43] LPS injection in a mice model of enterocolitis increased integrin expression in enterocytes, resulting in increased adhesion of enterocytes with ECM, blocking enterocyte migration to the injury sites to restore barrier integrity.[44] However, α_V integrins regulate other receptor pathways signaling, such as EGFR and TLRs, through less well-understood mechanisms.[45]*	1.7<br>1.5	0.8<br>0.6	5.3<br>14.9
TACSTD2	Tumor-associated calcium signal transducer 2<br>*Epithelial glycoprotein that regulates the stress fiber assembly. It is up-regulated by TNF-α, promoting colon cancer cell migration and invasion.[46]*	1.6	0.7	4.1
SDCBP	Syntenin-1	1.5	0.6	5.7
EPHA2	Ephrin type-A receptor 2<br>*Receptor tyrosine kinase binds membrane-bound ephrin-A family ligands residing on adjacent cells, leading to contact-dependent bidirectional signaling into neighboring cells. Activated by ephrin-A1, it regulates migration, integrin-mediated adhesion, proliferation, and differentiation of cells.*	1.5	0.6	4.1
EGFR	Epidermal growth factor receptor	1.5	0.6	9.1

	*It plays a crucial role in intestinal epithelial cell homeostasis. Its LPS activation leads to **maintaining the intestinal epithelial barrier** and protecting the epithelium from apoptosis.*[47] *It was found up-regulated by LPS promoting **intestinal epithelial cell growth and proliferation,***[48] *survival,*[47] *and restitution.*[49] *It is involved in the LPS-induction of COX-2 expression in enterocytes, a possible EGF mechanism in the inhibition of necrotizing enterocolitis.*[50] *Impaired EGFR signaling occurs and may be causal in multiple intestinal inflammatory conditions.*[51]			
OAT	Ornithine aminotransferase, mitochondrial<br>*Involved in cellular amino acid biosynthesis. It catalyzes the transformation of ornithine to P5C (1-pyrroline-5-carboxylate) and glutamate.*	1.4	0.5	5.6
ACSL5	Long-chain-fatty-acid--CoA ligase 5<br>*ACSL5 catalyzes the conversion of long-chain fatty acids to their active form acyl-CoA for both synthesis of cellular lipids, and degradation via beta-oxidation.*	1.4	0.5	5.0
	**Involved in lipid catabolism**			
LSR	Lipolysis-stimulated lipoprotein receptor<br>*LSR can also maintain a robust epithelial barrier, as its decrease causes epithelial barrier dysfunction, which is closely associated with physiological differentiation, cancer progression, and metastasis. LSR was broadly distributed to the lateral region.*[52]	1.4	0.5	3.1
ACOX1	Peroxisomal acyl-coenzyme A oxidase 1<br>*ACOX1 catalyzes the desaturation of acyl-CoA to 2-trans-enoyl-CoAs. The first enzyme of the fatty acid beta-oxidation pathway.*	1.3	0.4	2.8
NAMPT	Nicotinamide phosphoribosyltransferase<br>*NAMPT is the rate-limiting enzyme in the NAD^+ pathway converting nicotinamide to nicotinamide mononucleotide. Its secreted form acts as a cytokine.*	1.3	0.4	10.6
MVP	Major vault protein	1.3	0.4	3.0
CTSH	Pro-cathepsin H<br>*Lysosomal cysteine protease important in the overall degradation of lysosomal proteins.*	1.3	0.4	3.0
ATP1B1	Na/K-transporting ATPase subunit beta-1<br>*It is the β subunit of Na^+/K^+-ATPase, regulating the number of ATPases transported to the epithelial cells' basolateral membrane. It is involved in cell adhesion and the establishment of epithelial cell polarity. Its increasing number could compensate for the diminished catalytic activity of the Na/K-ATPase, that was observed during inflammation.*[53] *The expression of ATP1B1 was also increased in granulocytes and platelets in patients with inflammation.*[54]	1.3	0.4	6.5
FAHD1	Acylpyruvase FAHD1, mitochondrial<br>*Involved in the TCA cycle.*	1.3	0.4	3.1
CD59	CD59 glycoprotein<br>*This glycoprotein is involved in the ER to Golgi protein transport and its increased levels during chronic inflammation can be used as a biomarker for tissue damage.*[55] *CD59 can also act as an LPS co-receptor in different cell types.*[56]	1.3	0.4	4.1
MSN	Moesin<br>*It connects the actin cytoskeleton to the plasma membrane, participating in immunologic synapse formation and controlling cell shape, adhesion, locomotion, and signaling (MSN plays an important role in LPS-induced TNF-α production).*[57]	1.3	0.3	5.9
KLK6	Kallikrein-6<br>*Secreted serine protease with activity against amyloid precursor protein, myelin, gelatin, casein, and ECM proteins (fibronectin, laminin, vitronectin, collagen). Via degradation of ECM proteins, it contributes to the invasive phenotype of cancer cells (Caco-2).*[58] *It also activates protease-activated receptors triggering intracellular Ca^{2+} flux and promoting T and B lymphocyte survival.*[59]	0.4	-1.2	6.1
VAMP8	Vesicle-associated membrane protein 8<br>*It mediates MUC2 mucin exocytosis from intestinal cells maintaining innate intestinal homeostasis; its two-fold down-regulation provides fragility of the intestinal mucosa and dysfunctional barrier, both observed in infectious colitis.*[60]	0.5	-1.0	2.6

DDX1	ATP-dependent RNA helicase DDX1<br>***Specific for DNA inflammatory responses**, as its knockdown significantly decreased virus-induced IFN-β production and NF-κB activation.*[61,62]	0.5	-0.9	5.9
UNC93B1	Protein unc-93 homolog B1<br>*ER membrane protein that plays an important role in innate and adaptive immunity by regulating the trafficking of TLRs from the ER to endolysosomes. UNC93B1 deficiency resulted in the near-complete loss of TLR proteins in macrophages, showing that it is **critical also for maintaining TLR expression**.*[64]	0.5	-1.0	3.5
PNP	Purine nucleoside phosphorylase<br>*It is involved in the nicotinamide riboside catabolic process.*	0.6	-0.7	2.2
FABP5	Fatty acid-binding protein, epidermal<br>*Intracellular carrier for long-chain fatty acids, that selectively delivers to the nucleus, in addition to cytosolic transport. Its LPS down-regulation agrees with other studies revealing that the **LPS-induced loss of FABP5 was correlated with higher expression of anti-inflammatory/ regulatory factors**.*[65]	0.6	-0.7	5.0
SLC12A2	Solute carrier family 12 member 2<br>*NKCC1 is the major basolateral Cl^- entry pathway in intestinal epithelial cells. As Cl^--sensing signaling pathways control cell anti-inflammatory responses, SLC12A2-deficiency (leading to low Cl^- levels in IBD)*[66] *establishes pro-inflammatory and oxidative stress-associated gene programs.*[67] *The low intracellular Na^+ levels due to down-regulated NKCC1, correlate well with the impairment of Na^+/K^+-ATPase (exporting Na^+ to the extracellular space) in inflamed intestinal conditions.*[66,68] *It is also involved in cell adhesion, as in NKCC1 knockout mice, expression of claudin-2 (a constituent of tight junctions) was decreased.*[69] *NKCC1 expression was decreased by IFN-γ, in intestinal inflamed tissue vs. controls, and it was proposed as a potential biomarker of chronic colonic inflammation in UC.*[66]	0.7	-0.8	4.4
TMUB1	Transmembrane and ubiquitin-like domain-containing protein 1<br>*Transmembrane ubiquitin-like protein that shuttles between nucleus and cytoplasm during cell cycle progression, causing cell cycle arrest in the G0/G1 phase. Moreover, through direct binding to the E3 ubiquitin ligase TRAF6, it enhances NF-κB activation, increasing transcription of inflammatory mediators, through the reduction of IκBα stability.*[70]	0.7	-0.5	2.7
EPRS	Bifunctional glutamate/proline-tRNA ligase<br>*It is involved in the translation after INF-γ treatment.*[71]	0.7	-0.6	3.3
DKC1	H/ACA ribonucleoprotein complex subunit 4<br>*It is involved in rRNA processing.*	0.7	-0.4	4.3
HIST1H2BL	Histone H2B type 1-L	0.7	-0.5	5.3
ST14	Suppressor of tumorigenicity 14 protein<br>*The gene encodes a membrane-anchored serine protease (matriptase) that **strengthens the intestinal epithelial barrier** by promoting tight junction formation. Its intrinsic defect in the intestinal epithelial barrier was proposed to contribute to the inappropriate immune response to the commensal microbiota that underlies IBD.*[72]	0.7	-0.5	4.8
BTF3	Transcription factor BTF3<br>Also **found down-leveled after immune-stimulation** of THP1 cells.[73]	0.7	-0.5	3.1
ATP2A2	Sarcoplasmic/endoplasmic reticulum calcium ATPase 2<br>*It is a magnesium-dependent enzyme catalyzing ATP hydrolysis coupled with the translocation of Ca^{2+} from the cytosol to the ER lumen. Its decrease is consistent with the bibliography, as in IECs under inflammatory conditions the levels of cytoplasmic Ca^{2+} were high.*[74] *UV irradiation and proinflammatory cytokines (IL-6, IL-8, TNFα) decrease its expression via the IKK/IκB/NF-κB pathway and impair calcium uptake in the ER.*[75]	0.7	-0.5	3.7
MAT2B	Methionine adenosyltransferase 2 subunit beta<br>*An enzyme that catalyzes the formation of S-adenosylmethionine from methionine and ATP, shown to be **involved in chronic inflammation**.*[76]	0.7	-0.5	2.7
SPINT2	Kunitz-type protease inhibitor 2	0.7	-0.5	5.5
KDELC2	KDEL motif-containing protein 2	0.8	-0.5	4.7

SYPL1	Synaptophysin-like protein 1<br>*SYPL1 expression is positively correlated with antioxidant activity, as ROS were upregulated in cells with SYPL1 knockdown and vice versa.*[77]	0.8	-0.4	3.6
MRPL37	39S ribosomal protein L37, mitochondrial<br>*It is involved in the translation of mt-mRNA to the 13 mitochondrial ETC proteins.*	0.8	-0.4	2.9
HIST1H2AJ	Histone H2A type 1-J	0.7	-0.4	9.1
H2AFY	Core histone macro-H2A.1	0.7	-0.4	3.1
HIST1H4A	Histone H4	0.8	-0.4	7.5
**T6 hours LPS-modified proteins, in cytosol**				
EHD1	EH domain-containing protein 1<br>*It acts in the traffic of recycling endosomes, and the formation of the ciliary vesicles, and is associated with IGF-1 receptor endocytosis. Its overexpression in ovary cells repressed IGF-1-mediated signaling.*[78]	2.0	1.0	6.4
TUBB2B	Tubulin beta-2B chain	1.7	0.7	2.1
TACSTD2	Tumor-associated calcium signal transducer 2	1.7	0.7	4.1
ITGAV<br>ITGA6	Integrin alpha-V<br>Integrin alpha-6	1.6<br>1.3	0.6<br>0.4	2.3<br>8.7
CLIC4	Chloride intracellular channel protein 4	1.6	0.6	2.6
TXNRD1	Thioredoxin reductase 1, cytoplasmic	1.5	0.6	4.0
NAMPT<br><br><br><br>NNMT	**<u>Involved in NAD biosynthesis</u>**<br>Nicotinamide phosphoribosyltransferase<br>*NAMPT is the rate-limiting enzyme in the NAD^+ pathway converting nicotinamide to nicotinamide mononucleotide. Its secreted form acts as a cytokine.*<br>Nicotinamide N-methyltransferase<br>*NNMT catalyzes the N-methylation of nicotinamide and other pyridines using S-adenosyl methionine. NNMT and NAMPT were* ***up-regulated after microbial infection****.*[79] *The interplay between lipid metabolism and inflammation in metabolic active tissues is well documented.*[80,81] *The dramatic change in the NAD/NADH redox ratio is a typical result of the inflammatory response, known for its high energy consumption.*[82]	1.5<br><br><br><br>1.3	0.6<br><br><br><br>0.4	14.5<br><br><br><br>3.2
EGFR	Epidermal growth factor receptor<br>*It plays a crucial role in intestinal epithelial cell homeostasis. Its LPS activation leads to* ***maintaining the intestinal epithelial barrier*** *and protecting the epithelium from apoptosis.*[47] *It was found up-regulated by LPS promoting* ***intestinal epithelial cell growth and proliferation,***[48] *survival,*[47] *and restitution.*[49] *It is involved in the LPS-induction of COX-2 expression in enterocytes, a possible EGF mechanism in the inhibition of necrotizing enterocolitis.*[50] *Impaired EGFR signaling occurs and may be causal in multiple intestinal inflammatory conditions.*[51]	1.4	0.4	4.0
ATP1B1	Sodium/potassium-transporting ATPase subunit β	1.3	0.4	5.6
CD59	CD59 glycoprotein	1.3	0.4	6.5
KLK6	Kallikrein-6	0.4	-1.3	5.3
APP	Amyloid beta A4 protein	0.5	-1.1	1.6
IRGQ	Immunity-related GTPase family Q protein	0.6	-0.9	1.7
SPINT2	Kunitz-type protease inhibitor 2	0.6	-0.7	4.5
CALU	Calumenin	0.7	-0.6	2.5
RBBP4	Histone-binding protein RBBP4	0.7	-0.5	3.4
FTH1	Ferritin heavy chain<br>*Ferritin is the main intracellular and plasma protein that stores and releases iron in a controlled fashion. Its heavy chain (FTH1) has high ferroxidase activity converting Fe^{2+} to Fe^{3+} and plays a protective and anti-inflammatory role against iron-induced oxidative stress and cell death.*[83] *The light chain of ferritin, mainly associated with iron nucleation and stabilization of assembled ferritin, also had anti-inflammatory properties on LPS-induced inflammation in murine macrophages and was decreased after 2-6 h LPS.*[84] *After infection, the*	0.7	-0.5	3.8

	decreased FTH1 expression was correlated with increased expression of apoptosis-related genes.[83]			
SLC3A2	4F2 cell-surface antigen heavy chain<br>*The transmembrane glycoprotein CD98, encoded by the SLC3A2 gene, forms heteromeric amino acid transporters by interacting with different subunits. The precise substrate specificity depends on the other subunit in the heterodimer. Cancer cells overexpress CD to meet their increased nutritional demands, while down-regulation of CD98 triggers a dramatic reduction in the nucleotide pool, which leads to replicative stress in these cells.*[85] *Furthermore,* ***SLC3A2, located in the basolateral membrane of intestinal epithelial cells****, was regulated in inflammatory conditions, such as inflammatory bowel disease (IBD), but its pathogenic role remains unknown.*[86]	0.7	-0.5	2.8
VAPA	Vesicle-associated membrane protein-associated protein A<br>*VAPA plays a role in vesicle trafficking, and when recruited to the plasma membrane attenuates integrin beta-1 (ITGB1) activation.*[87]	0.7	-0.5	2.7
CD47<br>GLG1<br>ITGA3	Leukocyte surface antigen CD47<br>Golgi apparatus protein 1<br>Integrin alpha-3<br>*Cell-adhesion proteins in endothelial cells, mediate the binding and migration of neutrophils after LPS. GLG1 (or E-selectin ligand-1), is expressed in response to stimulation by inflammatory cytokines and/or bacterial LPS.* ***CD47 deficiency protected mice from LPS-induced lung injury***[88] *and ITGA3 deletion improved survival in septic mice.*[89]	0.7<br>0.8<br>0.7	-0.5<br>-0.4<br>-0.5	2.9<br>3.1<br>2.4
ECI1	Enoyl-CoA delta isomerase 1, mitochondrial	0.7	-0.5	4.3
ESYT1	Extended synaptotagmin-1	0.7	-0.5	5.9
PDIA4<br>P4HB<br>PDIA6	Protein disulfide-isomerase A4<br>Protein disulfide-isomerase<br>Protein disulfide-isomerase A6<br>*PDIs are key enzymes and chaperones localized in the endoplasmic reticulum (ER) catalyzing oxidative folding and preventing misfolding/aggregation of proteins.* ***PDIA4 inactivation restores a classical mitochondrial apoptosis pathway****, while the knockdown of PDIA6 favors a non-canonical cell death pathway sharing some necroptosis features.*[90]	0.7<br>0.8<br>0.8	-0.4<br>-0.4<br>-0.3	8.0<br>8.3<br>5.2
HYOU1	Hypoxia up-regulated protein 1<br>*Its negative mutations are associated with immunodeficiency.*[91]	0.7	-0.4	4.9
LRRC59	Leucine-rich repeat-containing protein 59<br>*ER membrane protein plays an important role in innate and adaptive immunity by regulating the trafficking of TLRs from the ER to endolysosomes, activating signaling cascades. Knockdown of LRRC59 reduced TLRs-mediated signaling.*[63]	0.7	0.5	2.9
SF3B4	Splicing factor 3B subunit 4<br>*It plays a key role in translation control, and its abundant expression results in abnormal collagen biosynthesis.*[92]	0.8	-0.4	4.5
SKIV2L2	Superkiller viralicidic activity 2-like 2	0.8	-0.4	3.3
ASPH	Aspartyl/asparaginyl beta-hydroxylase<br>*ASPH catalyzes posttranslational hydroxylation of β carbons of specific residues in certain EGF-like domains present in several proteins, including receptors and receptor ligands involved in cell growth and differentiation, and extracellular matrix molecules. It* ***is a key player in the malignant transformation*** *of solid tumors by enhancing cell proliferation, migration, and invasion.*[93]	0.8	-0.4	2.9
ERP44	Endoplasmic reticulum resident protein 44<br>*It mediates thiol-dependent retention in the early secretory pathway and is involved in the control of oxidative protein folding.*[94]	0.8	-0.4	6.4
HIST1H2BL	Histone H2B type 1-L	0.8	-0.4	5.2
OGDH	2-oxoglutarate dehydrogenase, mitochondrial<br>*Key enzyme in the TCA cycle. Its mRNA and protein levels* ***were decreased after LPS*** *in macrophages.*[95] *Our results agree with the traditional view that after LPS treatment, the efficiency of the TCA cycle is impaired, leading to the accumulation of metabolites (citric and succinic acid). Their accumulation increases ROS levels, which in turn inhibit the oxidative phosphorylation process and ATP formation, in*	0.8	-0.4	3.3

	favor of increased glycolysis, along with altered metabolism of fatty acids.[95,96]			
CANX<br>CALR	Calnexin<br>Calreticulin<br>*Multifunctional Ca^{2+}-binding proteins with a dual immunological pro- and anti-inflammatory role in different pathological conditions and animal models.*[97] ***In IECs they act as chaperons,*** *which retain in ER the newly synthesized MD2 and ensure its correct folding in acute inflammation in vivo. Interestingly, 1h-LPS increased MD2 binding to calnexin-calreticulin in IEC. However, in IBD this binding is altered, leading to MD2 proteolysis and impaired LPS recognition and hypo responsiveness.*[98]	0.8<br>0.8	-0.4<br>-0.3	3.4<br>9.7
RUFY1	RUN and FYVE domain-containing protein 1	0.8	-0.4	3.1
FKBP2	Peptidyl-prolyl cis-trans isomerase FKBP2<br>*It accelerates protein folding and plays fundamental roles in the regulation of signaling pathways involved in inflammation, adaptive immune responses, cancer, and developmental biology.*[99]	0.8	-0.4	3.3
PGRMC1	Membrane-associated progesterone receptor component 1<br>*PGRMC1 is an important novel mediator of progesterone (P4) function, attenuating the cytokine-induced MMP-9 activity, involved in extracellular matrix remodeling. Its mRNA expression was* ***decreased by H_2O_2*** *(100 µmol/L) treatment in amnion cells, which ultimately resulted in the downregulation of the PGRMC1 protein.*[100]	0.8	-0.4	3.5
SLC25A5	ADP/ATP translocase 2	0.8	-0.4	4.9
HNRNPU<br>HNRNPA3<br>HNRNPA1	Heterogeneous nuclear ribonucleoprotein U<br>Heterogeneous nuclear ribonucleoprotein A3<br>Heterogeneous nuclear ribonucleoprotein A1<br>*After TLR stimulation by LPS, they translocate from the nucleus to the cytoplasm, where they bind to mRNA of proinflammatory cytokines and positively regulate their expression by stabilizing mRNA.*[101]	0.8<br>0.8<br>0.8	-0.4<br>-0.4<br>-0.3	4.4<br>4.2<br>7.6
HSPA5	78 kDa glucose-regulated protein<br>*It plays a key role in protein folding and quality control* ***when down-regulated in the lung endothelium and protects against LPS-induced lung inflammatory responses.***[102]	0.8	-0.3	5.8
IMMT	MICOS complex subunit MIC60	0.8	-0.3	3.3
HSP90B1	Endoplasmin<br>*Molecular chaperone that functions in the processing and transport of secreted proteins. It is required for the proper folding of TLRs.*[103] *It has ATPase activity and participates in the unfolding of IL-1 to facilitate their translocation from the ER to Golgi and secretion.*[104]	0.8	-0.3	4.5
NONO	Non-POU domain-containing octamer-binding protein<br>*NONO is involved in transcriptional regulation.*	0.8	-0.3	5.3
HIST1H4A	Histone H4	0.8	-0.3	10.0
HIST1H2AJ	Histone H2A type 1-J	0.8	-0.3	8.5
HNRNPA2B1	Heterogeneous nuclear ribonucleoproteins A2/B1	0.8	-0.3	6.8
FKBP2	Peptidyl-prolyl cis-trans isomerase FKBP2	0.8	-0.4	3.3

Table S.2.2 Up- and down-leveled proteins after 6-h LPS **Mn1-, Mn1** alone, and LPS $MnCl_2$-stimulation of HT29-MD2 cells, vs. T0 (non-incubated or basal sample). The headlines of the LPS **Mn1** condition are in orange, **Mn1** in dark orange, and LPS $MnCl_2$ in green, as for the corresponding figure. Down-leveled proteins are highlighted in yellow.

Protein	Protein name	Fold-change	Log2 difference	-Log (P-value)
	**T6 hours LPS Mn1-modified proteins, in PMO**			
PLAUR	Urokinase plasminogen activator surface receptor	3.6	1.8	4.5
DNAJB1	DnaJ homolog subfamily B member 1	2.2	1.2	6.8
NAPRT	Nicotinate phosphoribosyltransferase	1.7	0.8	5.3
ARHGAP1	Rho GTPase-activating protein 1	1.7	0.7	1.8
ARHGDIB	Rho GDP-dissociation inhibitor 2	1.7	0.7	3.7

LOXL4	Lysyl oxidase homolog 4	1.6	0.7	2.8
DARS	Aspartate--tRNA ligase, cytoplasmic	1.6	0.7	4.4
EPS8L2	Epidermal growth factor receptor kinase substrate 8-like protein 2	1.5	0.6	4.0
SERPINB1	Leukocyte elastase inhibitor	1.5	0.6	4.5
GGCT	Gamma-glutamylcyclotransferase	1.5	0.6	2.1
RPS6	40S ribosomal protein S6	1.5	0.6	5.4
ARHGDIA	Rho GDP-dissociation inhibitor 1	1.5	0.5	3.0
EEF1A2	Elongation factor 1-alpha 2	1.5	0.5	3.5
CSTB	Cystatin-B	1.5	0.5	3.8
SORL1	Sortilin-related receptor	1.5	0.5	4.6
SOD2	Superoxide dismutase [Mn], mitochondrial	1.5	0.5	2.4
PDCD6IP	Programmed cell death 6-interacting protein	1.4	0.5	2.8
RPS28	40S ribosomal protein S28	1.4	0.5	5.0
CAPN1	Calpain-1 catalytic subunit	1.4	0.5	3.4
PDCD6	Programmed cell death protein 6	1.4	0.5	4.0
CYBA	Cytochrome b-245 light chain	1.4	0.5	3.3
HSPH1	Heat shock protein 105 kDa	1.4	0.5	4.1
SERPINB6	Serpin B6	1.3	0.4	3.2
OTUB1	Ubiquitin thioesterase OTUB1	1.3	0.4	3.7
SKP1	S-phase kinase-associated protein 1	1.3	0.4	5.5
PRDX2	Peroxiredoxin-2	1.3	0.4	3.9
CAP1	Adenylyl cyclase-associated protein 1	1.3	0.4	3.7
H3F3A	Histone H3.3	1.3	0.4	4.9
YWHAG	14-3-3 protein gamma	1.3	0.3	4.6
ESD	S-formylglutathione hydrolase	1.3	0.3	3.7
PARK7	Protein deglycase DJ-1	1.2	0.3	4.9
TGM2	Protein-glutamine gamma-glutamyltransferase 2	1.2	0.3	12.6
KPNB1	Importin subunit beta-1	1.2	0.3	6.9
IGFBP4	Insulin-like growth factor-binding protein 4	0.5	-1.0	3.0
CYP51A1	Lanosterol 14-alpha demethylase	0.5	-1.0	2.2
EIF3B	Eukaryotic translation initiation factor 3 subunit B	0.5	-0.9	5.6
TRIM28	Transcription intermediary factor 1-beta	0.5	-0.9	9.2
CLSTN1	Calsyntenin-1	0.5	-0.9	3.6
NDUFA10	NADH dehydrogenase [ubiquinone] 1 alpha subcomplex subunit 10, mitochondrial	0.6	-0.8	2.0
GRSF1	G-rich sequence factor 1	0.6	-0.8	2.2
KLK6	Kallikrein-6	0.6	-0.8	6.5
DNAJC3	DnaJ homolog subfamily C member 3	0.6	-0.7	3.1
PSMC4	26S protease regulatory subunit 6B	0.6	-0.6	6.3
GOLGB1	Golgin subfamily B member 1	0.6	-0.6	2.9
PSMA5	Proteasome subunit alpha type-5	0.7	-0.6	3.4
GTF2I	General transcription factor II-I	0.7	-0.6	7.1
ATP6AP1	V-type proton ATPase subunit S1	0.7	-0.6	1.9
HLA-C	HLA class I histocompatibility antigen	0.7	-0.6	2.3
CALU	Calumenin	0.7	-0.5	6.6
GOLIM4	Golgi integral membrane protein 4	0.7	-0.5	2.1
NDUFS1	NADH-ubiquinone oxidoreductase, mitochondrial	0.7	-0.5	4.7
TRIM29	Tripartite motif-containing protein 29	0.7	-0.5	4.9

AGRN	Agrin	0.7	-0.5	2.6
FKBP4	Peptidyl-prolyl cis-trans isomerase FKBP4	0.7	-0.5	3.4
PUF60	Poly(U)-binding-splicing factor PUF60	0.7	-0.4	3.5
SNRNP70	U1 small nuclear ribonucleoprotein 70 kDa	0.7	-0.4	5.0
PSAP	Prosaposin	0.7	-0.4	4.8
LNPEP	Leucyl-cystinyl aminopeptidase	0.7	-0.4	2.5
FAM129B	Niban-like protein 1	0.7	-0.4	6.5
BCLAF1	Bcl-2-associated transcription factor 1	0.7	-0.4	4.4
FAM3C	Protein FAM3C	0.7	-0.4	5.2
THRAP3	Thyroid hormone receptor-associated protein 3	0.8	-0.4	3.7
SPINT2	Kunitz-type protease inhibitor 2	0.8	-0.4	4.6
SNRPA	U1 small nuclear ribonucleoprotein A	0.8	-0.4	3.0
SF3A3	Splicing factor 3A subunit 3	0.8	-0.4	8.3
CDCP1	CUB domain-containing protein 1	0.8	-0.4	4.8
MATR3	Matrin-3	0.8	-0.4	6.2
KHDRBS1	KH domain-containing, RNA-binding, signal transduction-associated protein 1	0.8	-0.4	5.2
MANF	Mesencephalic astrocyte-derived neurotrophic factor	0.8	-0.4	4.3
SRSF6	Serine/arginine-rich splicing factor 6	0.8	-0.3	4.4
PLOD3	Procollagen-lysine, 2-oxoglutarate 5-dioxygenase 3	0.8	-0.3	4.6
SNRPD2	Small nuclear ribonucleoprotein Sm D2	0.8	-0.3	7.2
**T6 hours LPS Mn1-modified proteins, in cytosol**				
FKBP9	Peptidyl-prolyl cis-trans isomerase FKBP9	1.9	0.9	2.2
H2AFY	Core histone macro-H2A.1	1.8	0.9	2.2
DNAJB1	DnaJ homolog subfamily B member 1	1.7	0.7	3.8
GCNT3	Beta-1,3-galactosyl-O-glycosyl-glycoprotein beta-1,6-N-acetylglucosaminyltransferase 3	1.7	0.7	2.2
FAM3C	Protein FAM3C	1.6	0.7	8.0
HMGCS1	Hydroxymethylglutaryl-CoA synthase, cytoplasmic	1.6	0.6	4.9
ELAVL1	ELAV-like protein 1	1.5	0.6	6.5
SMC1A	Structural maintenance of chromosomes protein 1A	1.5	0.6	4.2
POFUT1	GDP-fucose protein O-fucosyltransferase 1	1.5	0.6	3.7
KDELC2	KDEL motif-containing protein 2	1.4	0.5	4.2
DNAJA1	DnaJ homolog subfamily A member 1	1.4	0.5	3.0
GALNT7	N-acetylgalactosaminyltransferase 7	1.4	0.5	3.6
EFTUD2	116 kDa U5 small nuclear ribonucleoprotein component	1.4	0.5	6.9
TM9SF4	Transmembrane 9 superfamily member 4	1.4	0.5	4.9
HNRNPC	Heterogeneous nuclear ribonucleoproteins C1/C2	1.4	0.5	4.3
EML2	Echinoderm microtubule-associated protein-like 2	1.3	0.4	4.0
ZC3HAV1	Zinc finger CCCH-type antiviral protein 1	0.4	-1.4	3.9
RBM10	RNA-binding protein 10	0.5	-1.1	1.7
EPHA2	Ephrin type-A receptor 2	0.5	-1.0	3.8
PGM2	Phosphoglucomutase-2	0.5	-1.0	2.4
TRIM28	Transcription intermediary factor 1-beta	0.5	-0.9	7.5
IGFBP4	Insulin-like growth factor-binding protein 4	0.5	-0.9	2.6
ARL1	ADP-ribosylation factor-like protein 1	0.6	-0.8	4.2
EPHX1	Epoxide hydrolase 1	0.6	-0.8	4.0
RTN4	Reticulon-4	0.6	-0.8	2.1

KLK6	Kallikrein-6	0.6	-0.7	3.8
RBM4	RNA-binding protein 4; RNA-binding protein 4B	0.6	-0.7	2.4
HNRNPH3	Heterogeneous nuclear ribonucleoprotein H3	0.6	-0.7	3.3
SPINT2	Kunitz-type protease inhibitor 2	0.7	-0.6	4.4
STIM1	Stromal interaction molecule 1	0.7	-0.6	5.7
CHCHD3	MICOS complex subunit MIC19	0.7	-0.6	4.7
APLP2	Amyloid-like protein 2	0.7	-0.6	3.8
TSPAN6	Tetraspanin-6	0.7	-0.6	3.3
CRIP2	Cysteine-rich protein 2	0.7	-0.6	2.6
CTSC	Dipeptidyl peptidase 1	0.7	-0.5	4.9
NOMO2	Nodal modulator 2	0.7	-0.5	5.2
ST14	Suppressor of tumorigenicity 14 protein	0.7	-0.5	6.8
U2AF1	Splicing factor U2AF 35 kDa subunit	0.7	-0.4	4.4
CANX	Calnexin	0.7	-0.4	4.8
KHDRBS1	KH domain-containing, RNA-binding, signal transduction-associated protein 1	0.8	-0.4	5.9
**T6 hours Mn1-modified proteins, in PMO**				
PLAUR	Urokinase plasminogen activator surface receptor	3.7	1.9	4.3
TACSTD2	Tumor-associated calcium signal transducer 2	1.4	0.5	7.3
CTSH	Pro-cathepsin H	1.4	0.5	5.1
CNBP	Cellular nucleic acid-binding protein	0.4	-1.4	3.4
IGFBP4	Insulin-like growth factor-binding protein 4	0.4	-1.3	4.9
KLK6	Kallikrein-6	0.5	-0.9	6.0
DNAJC3	DnaJ homolog subfamily C member 3	0.5	-0.9	3.8
PDIA5	Protein disulfide-isomerase A5	0.6	-0.7	2.7
LAMA5	Laminin subunit alpha-5	0.6	-0.7	5.6
APLP2	Amyloid-like protein 2	0.6	-0.6	3.5
AGRN	Agrin	0.6	-0.7	3.4
PSAP	Prosaposin	0.6	-0.6	8.7
NDUFA10	NADH dehydrogenase [ubiquinone] 1 alpha subcomplex subunit 10, mitochondrial	0.7	-0.6	5.1
DKC1	H/ACA ribonucleoprotein complex subunit 4	0.7	-0.6	5.0
TRIM28	Transcription intermediary factor 1-beta	0.7	-0.5	9.2
RPS19	40S ribosomal protein S19	0.7	-0.4	5.5
OCIAD2	OCIA domain-containing protein 2	0.7	-0.4	5.0
GLG1	Golgi apparatus protein 1	0.7	-0.5	3.9
SQSTM1	Sequestosome-1	0.7	-0.5	3.9
H2AFY	Core histone macro-H2A.1	0.7	-0.5	3.7
RPL11	60S ribosomal protein L11	0.8	-0.4	5.4
GALNT7	N-acetylgalactosaminyltransferase 7	0.8	-0.4	5.1
RANBP1	Ran-specific GTPase-activating protein	0.8	-0.3	5.0
LEPRE1	Prolyl 3-hydroxylase 1	0.8	-0.4	4.8
SORL1	Sortilin-related receptor	0.8	-0.4	4.2
CALB2	Calretinin	0.8	-0.4	3.9
ARF4	ADP-ribosylation factor 4	0.8	-0.4	3.8
CALU	Calumenin	0.8	-0.4	3.7
**T6 hours Mn1-modified proteins, in cytosol**				
GLUL	Glutamine synthetase	1.9	0.9	2.2
RRS1	Ribosome biogenesis regulatory protein homolog	1.7	0.8	2.6
GTF2I	General transcription factor II-I	1.7	0.8	8.8
FAM3C	Protein FAM3C	1.6	0.7	7.5
CS	Citrate synthase, mitochondrial	1.6	0.7	5.4
HIST1H2AJ	Histone H2A type 1-J	1.6	0.6	16.2
LRPPRC	Leucine-rich PPR motif-containing protein, mitochondrial	1.6	0.6	4.6
LMO7	LIM domain only protein 7	1.6	0.6	2.6
HIST1H4A	Histone H4	1.5	0.6	9.3
H3F3A	Histone H3.3	1.5	0.5	10.1
LCN2	Neutrophil gelatinase-associated lipocalin	1.5	0.5	2.4
TM9SF4	Transmembrane 9 superfamily member 4	1.5	0.5	6.5

HIST1H2BL	Histone H2B type 1-L	1.4	0.5	5.7
SAFB2	Scaffold attachment factor B2	1.4	0.5	5.7
ETFA	Electron transfer flavoprotein subunit alpha, mitochondrial	1.4	0.5	4.5
ECHS1	Enoyl-CoA hydratase, mitochondrial	1.4	0.5	3.0
LMNA	Lamin-A/C	1.4	0.5	11.8
EML2	Echinoderm microtubule-associated protein-like 2	1.4	0.5	4.5
TPP1	Tripeptidyl-peptidase 1	1.4	0.5	3.8
HSPA9	Stress-70 protein, mitochondrial	1.4	0.4	5.8
ECH1	Delta(3,5)-Delta(2,4)-dienoyl-CoA isomerase, mitochondrial	1.4	0.4	4.2
HNRNPC	Heterogeneous nuclear ribonucleoproteins C1/C2	1.4	0.4	4.6
SNRPE	Small nuclear ribonucleoprotein E	1.4	0.4	3.1
HNRNPUL2	Heterogeneous nuclear ribonucleoprotein U-like protein 2	1.3	0.4	6.3
TACSTD2	Tumor-associated calcium signal transducer 2	1.3	0.4	3.8
HNRNPU	Heterogeneous nuclear ribonucleoprotein U	1.3	0.4	6.5
SLC35B2	Adenosine 3-phospho 5-phosphosulfate transporter 1	1.3	0.4	3.4
HNRNPM	Heterogeneous nuclear ribonucleoprotein M	1.3	0.4	4.5
PPT1	Palmitoyl-protein thioesterase 1	1.3	0.4	3.0
ILF2	Interleukin enhancer-binding factor 2	1.3	0.4	13.0
RBMX	RNA-binding motif protein, X chromosome	1.3	0.4	5.5
PRPF19	Pre-mRNA-processing factor 19	1.3	0.4	3.9
SNRNP200	U5 small nuclear ribonucleoprotein 200 kDa helicase	1.3	0.4	4.1
ILF3	Interleukin enhancer-binding factor 3	1.3	0.4	8.5
RALY	RNA-binding protein Raly	1.3	0.4	3.9
ITGB4	Integrin beta-4	1.3	0.4	4.6
EIF4A3	Eukaryotic initiation factor 4A-III	1.3	0.3	5.6
HDGF	Hepatoma-derived growth factor	1.3	0.3	3.5
SF3A1	Splicing factor 3A subunit 1	1.3	0.3	4.4
HNRNPA2B1	Heterogeneous nuclear ribonucleoproteins A2/B1	1.3	0.3	8.6
HNRNPH2	Heterogeneous nuclear ribonucleoprotein H2	1.3	0.3	5.2
CTSB	Cathepsin B	1.3	0.3	5.2
APEX1	DNA-(apyrimidinic site) lyase	1.3	0.3	3.8
PRKDC	DNA-dependent protein kinase catalytic subunit	1.2	0.3	5.5
MDH2	Malate dehydrogenase, mitochondrial	1.2	0.3	5.2
PABPN1	Polyadenylate-binding protein 2	1.2	0.3	4.5
HMGB1	High mobility group protein B1	1.2	0.3	5.0
CNBP	Cellular nucleic acid-binding protein	0.2	-2.5	8.9
SPINT2	Kunitz-type protease inhibitor 2	0.6	-0.7	4.6
IGFBP4	Insulin-like growth factor-binding protein 4	0.6	-0.7	1.9
COA7	Cytochrome c oxidase assembly factor 7	0.6	-0.7	1.8
KLK6	Kallikrein-6	0.7	-0.6	2.8
FTH1	Ferritin heavy chain	0.7	-0.5	6.1
MBNL1	Muscleblind-like protein 1	0.7	-0.5	2.5
SQSTM1	Sequestosome-1	0.7	-0.5	3.2
BZW2	Basic leucine zipper and W2 domain-containing protein 2	0.7	-0.5	3.1
CHORDC1	Cysteine and histidine-rich domain-containing protein 1	0.7	-0.4	6.4
PDLIM5	PDZ and LIM domain protein 5	0.7	-0.4	4.0
EIF4A2	Eukaryotic initiation factor 4A-II	0.7	-0.4	3.8
CPOX	Oxygen-dependent coproporphyrinogen-III oxidase, mitochondrial	0.8	-0.4	4.1
HYOU1	Hypoxia up-regulated protein 1	0.8	-0.4	3.6
PABPC4	Polyadenylate-binding protein 4	0.8	-0.4	4.2
TRIM25	E3 ubiquitin/ISG15 ligase TRIM25	0.8	-0.3	4.3
SRM	Spermidine synthase	0.8	-0.3	5.1
ARF4	ADP-ribosylation factor 4	0.8	-0.3	6.2
TRIM28	Transcription intermediary factor 1-beta	0.8	-0.3	6.5
CCDC58	Coiled-coil domain-containing protein 58	0.8	-0.3	8.0
AK2	Adenylate kinase 2, mitochondrial	0.8	-0.3	7.8
**T6 hours LPS $MnCl_2$-modified proteins, in PMO**				
PLAUR	Urokinase plasminogen activator surface receptor	5.6	2.5	2.4
SOD2	Superoxide dismutase [Mn], mitochondrial	2.2	1.1	3.4
EHD1	EH domain-containing protein 1	2.0	1.0	6.8
SDCBP	Syntenin-1	1.6	0.7	4.1
TACSTD2	Tumor-associated calcium signal transducer 2	1.4	0.5	6.8

KLK6	Kallikrein-6	0.3	-1.6	6.9
IGFBP4	Insulin-like growth factor-binding protein 4	0.5	-1.1	2.6
TMEM165	Transmembrane protein 165	0.6	-0.8	3.3
SPINT2	Kunitz-type protease inhibitor 2	0.7	-0.6	4.0
PSAP	Prosaposin	0.7	-0.5	5.9
H3F3A	Histone H3.3	0.8	-0.4	7.6
HIST1H4A	Histone H4	0.8	-0.4	8.9
**T6 hours LPS $MnCl_2$-modified proteins, in cytosol**				
GLUL	Glutamine synthetase	2.1	1.1	5.7
SDCBP	Syntenin-1	2.0	1.4	5.5
CNPY2	Protein canopy homolog 2	1.9	0.9	7.4
EHD1	EH domain-containing protein 1	1.8	0.8	5.1
SAFB	Scaffold attachment factor B1	1.7	0.7	3.7
HNRNPC	Heterogeneous nuclear ribonucleoproteins C1/C2	1.4	0.5	8.3
ATP5B	ATP synthase subunit beta, mitochondrial	1.3	0.4	5.6
LMNA	Lamin-A/C	1.3	0.4	6.7
CAT	Catalase	1.3	0.4	7.7
HNRNPUL2	Heterogeneous nuclear ribonucleoprotein U-like protein 2	1.2	0.3	10.2
KLK6	Kallikrein-6	0.4	-1.3	4.3

Table S.2.3 Proteins oxidation level after 6-h LPS-stimulation of HT29-MD2 cells, in the PMO and cytosolic fraction, compared with the corresponding LPS **Mn1** and LPS $MnCl_2$ conditions. The oxidation status of a protein was measured by the increased *S-Ox peptides* (x/z)/T0 >1 and decreased *S-Red peptides* (y/z)/T0 <1. Cysteines are shown in bold red in the peptide sequence. Proteins in bold blue are discussed in the article. The PMO fractions are highlighted in blue.

**6-hour incubation**													
**Protein**	**Peptides with -Cys**	**Fold-Change S-Ox in PMO**			**Fold-Change S-Ox in Cyto**			**Fold-Change S-Red in PMO**			**Fold-Change S-Red in Cyto**		
		**LPS**	**LPS Mn1**	**LPS $MnCl_2$**	**LPS**	**LPS Mn1**	**LPS $MnCl_2$**	**LPS**	**LPS Mn1**	**LPS $MnCl_2$**	**LPS**	**LPS Mn1**	**LPS $MnCl_2$**
ABHD10	EAEHH**Cys229**LLHSPIPVN**Cys239**PIR							0.9					
ACTN4	**Cys352**QLEINFNTLQTK							0.7			0.6	0.7	
ACTG1	**Cys257**PEALFQPSFLGMES**Cys272**GIHETTFNSIMK							0.9	1.0	1.0			
ADPGK	VAGTQA**Cys415**ATETIDTSR	1.1	1.0										
ALDH1A1	LYSNAYLNDLAG**Cys126**IK				1.7	1.2							
ALDOA	YASI**Cys178**QQNGIVPIVEPEILPDGDHDLKR				1.1	1.0							
	YASI**Cys178**QQNGIVPIVEPEILPDGDHDLK				1.1	1.1							
	VNP**Cys73**IGGVILFHETLYQK										0.9	1.0	1.0
ANXA1	**Cys270**ATSKPAFFAEK										0.9	0.9	1.0
ANXA2	GLGTDEDSLIEII**Cys133**SR				1.1	1.0	0.9						
APMAP	TRDDEPV**Cys149**GRPLGIR	1.5	1.4	1.0									
ATP1B1	DDMIFED**Cys126**GDVPSEPK	1.3	0.9	1.6	1.2	1.2							
CAT	LVNANGEAVY**Cys232**K	1.2	1.1	1.2	1.1	1.3							
CAPZB	DETVSD**Cys206**SPHIANIGR										0.9	1.0	1.0
CCT2	SLHDAL**Cys395**VLAQTVK										0.9	0.8	
CCT6A	NAIDDG**Cys406**VVPGAGAVEVAMAEALIK										0.9	0.9	
CCT7	**Cys345**QVFEETQIGGER										0.9		

CD55	IPGEKDSVICys81LK	1.7	1.0	1.1									
	EIYCys225PAPPQIDNGIIQGER	1.1	1.0	1.0	1.1	1.0							
CD59	LRENELTYYCys88Cys89K	1.7	1.0	1.2									
	FEHCys70NFNDVTTR	1.5	1.0	1.1	1.4	1.1	1.1						
CFL1	AVLFCys39LSEDKK *Oxidant-induced apoptosis is mediated by oxidation of the actin-regulatory protein cofilin.*							0.7			0.9	1.0	1.0
CLIC1	EEFASTCys223PDDEEIELAYEQVAK							0.6	1.1	0.9	0.9	1.0	1.0
CLTC	LPVVIGGLLDVDCys824SEDVIK										0.9		
CNBP	Cys141GETGHVAINCys151SK										0.9		0.9
COPG1	ALCys129QITDSTMLQAIER										0.9	0.7	
CTSD	AIGAVPLIQGEYMIPCys329EK				1.1	0.8	1.1	0.7			0.8	0.8	
DDX39B	NCys165PHIVVGTPGR										0.8	1.0	0.9
DSTN	HECys135QANGPEDLNR							0.9		1.0	0.9	0.9	0.9
EIF6	ASFENNCys11EIGCys15FAK							0.8			0.9	0.8	
ERGIC1	IPLNNGAGCys115R	1.1	1.0	1.0									
FASN	AINCys1448ATSGVVGLVNCys1459LR										0.8	0.8	
FLNB	ALGALVDSCys178APGLCys183PDWESWDPQKPVDNAR										0.9	0.9	
FUBP1	Cys328QHAAEIITDLLR										0.9		1.1
GAPDH	VPTANVSVVDLTCys247R							0.9	1.0	1.0			
GNAI2	EIYTHFTCys326ATDTK							0.9	1.1	1.0			
GNB1	ACys25ADATLSQITNNIDPVGR							0.9	0.9	0.9			
	QTFTGHESDINAICys233FFPNGNAFATGSDDATCys250R							0.9	1.0	1.0			
	ELAGHTGYLSCys148Cys149R							0.9	0.9	0.9			
GNB2	ELPGHTGYLSCys148Cys149R							0.9	1.1	1.0			
GOT2	EYLPIGGLAEFCys106K							0.9	1.0				
GPD2	Cys285VINATGPFTDSVR							0.9		1.0			
GPI	MIPCys404DFLIPVQTQHPIR				1.2	1.1							
HNRNPA1	YHTVNGHNCys175EVR										0.8	1.0	1.2
HNRNPA3	YHTINGHNCys196EVK										0.9	1.0	1.3
HNRNPF	GLPWSCys22SVEDVQNFLSDCys34TIHDGAAGVHFIYTR										0.9		
HNRNPL	ASLNGADIYSGCys260Cys261TLK										0.9	0.9	1.0
HSP90AA1	HGLEVIYMIEPIDEYCys529VQQLK										0.9	1.0	
	VFIMDNCys350EELIPEYLNFIR										0.9	1.0	
	LVTSPCys589Cys590IVTSTYGWTANMER										0.9	1.1	1.0
HSP90AB1	GFEVVYMTEPIDEYCys521VQQLK										0.9	0.9	0.8

	VFIMDSCys366DELIPEYLNFIR										0.9	0.9	
	RGFEVVYMTEPIDEYCys521VQQLK										0.9	0.9	
HSPA8	GPAVGIDLGTTYSCys17VGVFQHGK										0.9	0.9	
	Cys574NEIINWLDK										0.9	1.0	0.9
HSPA9	MEEFKDQLPADECys608NK										0.8		
HSPD1	Cys228EFQDAYVLLSEK										0.8		
	AAVEEGIVLGGGCys442ALLR										0.9	0.9	1.3
HSPH1	SVLDAAQIVGLNCys167LR							0.9	1.4	0.7	0.9	1.2	0.9
	KPVTDCys140VISVPSFFTDAER										0.8	1.4	
	SQFEELCys310AELLQK										0.9	1.2	0.9
	FICys658EQDHQNFLR										0.9	1.3	
IPO7	GIDQCys757IPLFVEAALER										0.9	1.0	
ITGB1	DKLPQPVQPDPVSHCys691K	1.2	0.9	1.0	1.3	0.9							
LGALS3BP	STSSFPCys561PAGHFNGFR	1.1	1.1	1.1				0.8					
NPC2	AVVHGILMGVPVPFPIPEPDGCys93K				1.2	0.8							
NUTF2	NINDAWVCys114TNDMFR				1.1	1.0							
PCNA	DLSHIGDAVVISCys162AK										0.9	0.9	1.0
PKM	AEGSDVANAVLDGADCys358IMLSGETAK							0.9	0.9	0.9			
	NTGIICys49TIGPASR										0.8	0.9	0.9
PPP1CA	TFTDCys155FNCys158LPIAAIVDEK							0.9					
**PRDX3**	AFQYVETHGEVCys229PANWTPDSPTIKPSPAASK				1.3	1.0							
**PRDX6**	DFTPVCys47TTELGR				1.1	1.1							
RACK1	YTVQDESHSEWVSCys153VR										0.9	1.0	
	HLYTLDGGDIINALCys240FSPNR										0.9	0.9	
	TNHIGHTGYLNTVTVSPDGSLCys207ASGGK										0.9	0.9	0.9
RPL10A	FSVCys66VLGDQQHCys74DEAK										0.9	0.9	0.9
RPL14	Cys54MQLTDFILK										0.9	0.8	
RPL23	ISLGLPVGAVINCys28ADNTGAK										0.9	0.9	1.0
RPL30	TGVHHYSGNNIELGTACys85GK										0.9	0.9	
RPL4	RGPCys208IIYNEDNGIIK										0.8		
RPL5	VGLTNYAAAYCys100TGLLLAR										0.9	0.9	
RPLP0	AGAIAPCys80EVTVPAQNTGLGPEK							0.9	1.0				
RPS12	QAHLCys50VLASNCys56DEPMYVK										0.9		
RPS3A	NCys96LTNFHGMDLTR										0.9	0.9	
	ACys201QSIYPLHDVFVR										0.9		1.0
	LFCys139VGFTK										0.9		

RPS4X	FDTGNLCys181MVTGGANLGR							0.9	0.9		0.9		
RPS5	TIAECys172LADELINAAK							0.9	0.8	0.9			
SDHA	ACALSIEESCys475RPGDK							0.9	1.1	1.1			
SHMT2	YYGGAEVVDEIELLCys119QR										0.8		
SOD1	GLTEGLHGFHVHEFGDNTAGCys58TSAGPHFNPLSR				1.3	1.1							
	HVGDLGNVTADKDGVADVSIEDSVISLSGDHCys112IIGR										0.9	0.7	1.0
SLC25A5	KGTDIMYTGTLDCys257WR							0.9		1.0			
SSB	ICys18HQIEYYFGDFNLPR										0.9	0.8	
STIP1	ALSVGNIDDALQCys26YSEAIK										0.9	0.9	0.9
TGM2	Cys10DLELETNGR										0.9	1.1	1.1
TPI1	DCys87GATWVVLGHSER				1.1	1.2	0.9						
	VAHALAEGLGVIACys127IGEK										0.9	0.9	
	IAVAAQNCys67YK				1.1	1.0	1.0						
TUBA1B	SIQFVDWCys347PTGFK										0.9	1.0	1.0
	AVCys376MLSNTTAIAEAWAR										0.9	1.0	1.0
TXNDC5	VDCys247TQHYELCys254SGNQVR	1.1	0.9	1.0									
UBE2D3	IYHPNINSNGSICys85LDILR										0.9	1.0	
VAT1	Cys50LVLTGFGGYDK										0.9	1.0	0.9
VCP	QAAPCys241VLFFDELDSIAK										0.9	0.9	1.0
VDAC2	PMCys13IPPSYADLGK							0.8					
YWHAQ	YLAEVACys134GDDRK										0.9	0.9	0.9

Table S.2.4 Up-regulated proteins after 15-min and 6-h LPS incubation vs. T0 (see Supplementary Table S.2.1, and S.2.5), classified according to their biological effect. In bold blue italics are highlighted the most important impacted proteins. For 6-h LPS see Fig. 3a (PMO) and Fig. 3c (Cytosolic) in the main text. For 15-min LPS see Fig. 6a (PMO) and Fig. S.2.4a (Cytosolic).

Biological processes	Proteins up-and down-regulated after 15-min LPS incubation vs. T0 (PMO fraction)	Proteins up- and down-regulated after 6-h LPS incubation vs. T0 (PMO and Cytosolic fractions)
**Metabolic processes**	***NUDT5*** [1.6], GOT1 [1.5], MDH1 [1.4], NIT2 [1.4], ALDOA [1.3], CMPK1 [1.3], GPI [1.3], LDHB [1.3], NME2 [1.3], OAT [1.3], TKT [1.3]	***EHD1*** [2.0], ACSL5 [1.4), LSR [1.4], OAT [1.4], ACOX1 [1.3], FAHD1 [1.3], NNMT [1.3], NAMPT [1.3 PMO, 1.5 Cytosolic]<br><br>***FABP5*** [0.6], PNP [0.6], MAT2B [0.7], ECI1 [0.7], PGRMC1 [0.8], KDELC2 [0.8], OGDH [0.8]
**Protein degradation / Apoptosis**	***UBE2L3*** [1.7], PSMD2 [1.7], PSMB6 [1.6], PSMA5 [1.5], PSMB2 [1.5], PDCD6 [1.5], CSTB [1.5], PSME1 [1.4], SERPINB1 [1.4], SERPINB6 [1.3]	***PLAUR*** [3.6], CTSH [1.3]<br><br>***KLK6*** [0.4], TMUB1 [0.7], ST14 [0.7]
**Cytoskeleton / ECM organization**	***MTPN*** [1.9], ***MYO1B*** [1.8], ARPC4 [1.4], CNN2 [1.4]	***TIMP1*** [2.2], ***TUBB2B*** [1.7], ***ITGAV*** [1.7 PMO, 1.6 Cytosolic], ***TACSTD2*** [1.6 PMO, 1.7 Cytosolic], SDCBP [1.5], ITGA2 [1.5], EPHA2 [1.5], MSN [1.3], ITGA6 [1.3]<br><br>CD47 [0.7], GLG1 [0.8], ITGA3 [0.7]

**Transport**	CUTA [1.5], MVP [1.5], TFG [1.4], NUTF2 [1.3]<br><br>SLC35B2 [0.7]	***LCN2*** [3.1], CLIC4 [1.6], MVP [1.3], CD59 [1.3], ATP1B1 [1.3]<br><br>***APP*** [0.5], ***VAMP8*** [0.5], ***UNC93B1*** [0.5], LRRC59 [0.7], SLC12A2 [0.7], ATP2A2 [0.7], FTH1 [0.7], SLC3A2 [0.7], VAPA [0.7], ESYT1 [0.7], CALR [0.8], HYOU1 [0.7], SLC25A5 [0.8], RUFY1 [0.8], HSP90B1 [0.8], SYPL1 [0.8]
**Antioxidant defense**	***SOD1*** [1.7], ALDH1A1 [1.5], NQO1 [1.4]	***SOD2*** [3.6], ***TXNRD1*** [1.5]<br><br>PDIA4 [0.7], P4HB [0.8], PDIA6 [0.8]
**Immunity**	***MIF*** [1.7], ***LTA4H*** [1.7], ***APRT*** [1.7], TSPAN6 [1.5]	
**RNA processing / DNA Replication / Chromatin organization**	RPS21 [1.6], YBX1 [1.3]<br><br>PCNA [0.7], H2AFY [0.7]	***DDX1*** [0.5], EPRS [0.7], DKC1 [0.7], BTF3 [0.7], RBBP4 [0.7], H2AFY [0.7], MRPL37 [0.8], SF3B4 [0.8], HNRNPU [0.8], HNRNPA1 [0.8], HNRNPA3 [0.8], NONO [0.8], HIST1H2BL [0.7 PMO, 0.8 Cytosolic], HIST1H2AJ [0.7 PMO, 0.8 Cytosolic], HIST1H4A [0.8 PMO, Cytosolic], HNRNPA2B1[0.8]
**Signaling**		ASPH [0.8]
**Chaperone**	ST13 [1.6]	CANX [0.8], HSPA5 [0.8], FKBP2 [0.8], ERP44 [0.8]
**Unknown function**	***C11orf54*** [1.8]	***IRGQ*** [0.6], SKIV2L2 [0.8]
**Mitogenic action**		EGFR [1.5 PMO, 1.4 Cytosolic]
**ETC Complex**	COX6B1 [0.6]	

B. 15 min

B.1. LPS and LPS Mn1 effects on the proteome level, after 15-min incubation

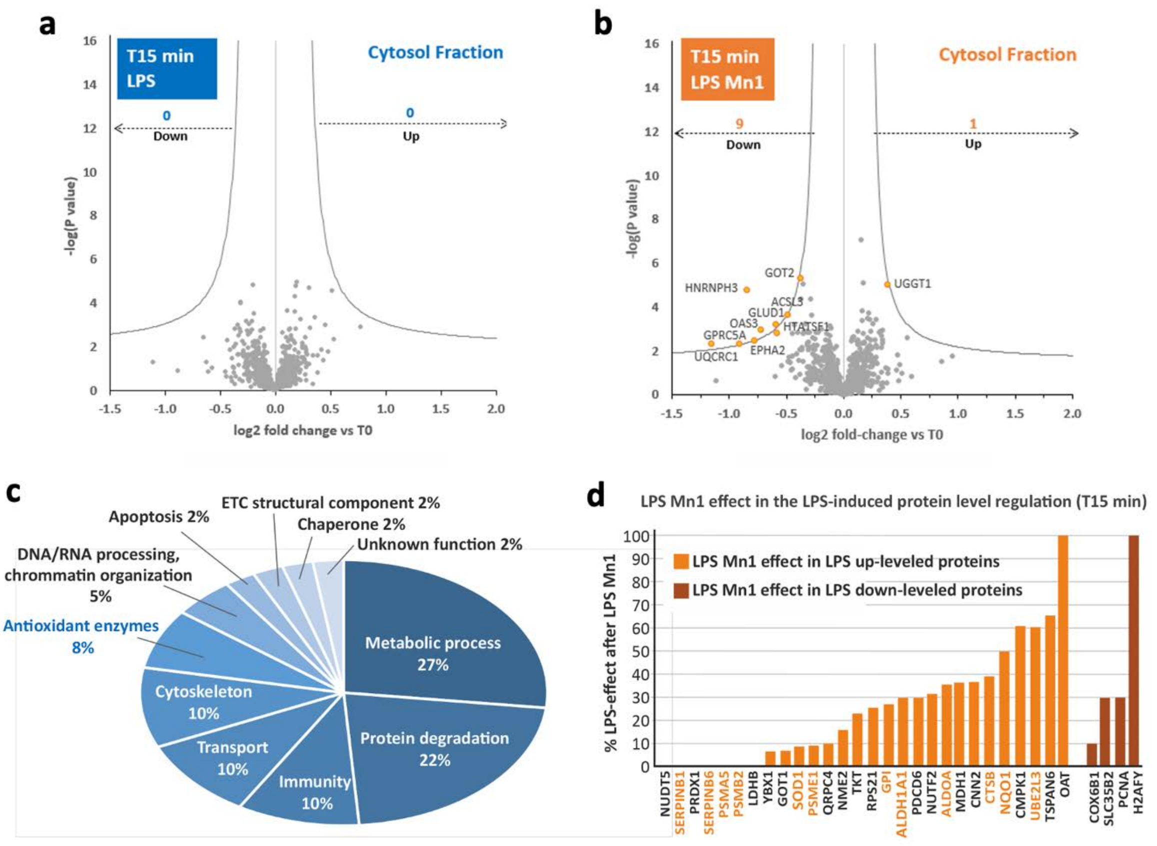

Figure S.2.4 Comparison of LPS and LPS Mn1, 15-min incubation

a and b. Effects on protein levels of 15-min incubation of LPS, and LPS Mn1 (vs. T0 sample**)** in cytosolic fractions of HT29-MD2 cells. LPS**,** and LPS **Mn1** effects were represented in volcano plots giving the t-test p-value (in log10 scale y-axis; significant if p-value < 0.01, 1 % error) against the fold-change between two chosen conditions (in log2 scale x-axis). The up- and down- leveled proteins in LPS **Mn1** are indicated with orange circles. The LPS condition is shown in blue and the LPS **Mn1** condition in orange.

c In a pie chart, the LPS up-leveled proteins are reported according to their biological function. See Tables S.2.4 and S.2.5 and Fig. 6 for complete detailed information.

d Histogram representing the effect of **Mn1** on the level of the proteins modulated by LPS, expressed as a percentage. A low percentage means that **Mn1** causes the protein level to return to basal, i.e. at T0 (e.g. for SOD1). A high percentage means that **Mn1** did not resolve the LPS-induced effect (e.g. for OAT). Proteins indicated in orange are those discussed in the text.

Table S.2.5 Up- and down-leveled proteins after 15-min LPS- and LPS **Mn1**-stimulation of HT29-MD2 cells, vs. T0 (non-incubated or basal sample). The headline of the LPS condition is shown in blue, the LPS **Mn1** condition is shown in orange (same color code for the corresponding figures). Down-leveled proteins are highlighted in yellow.

Protein	Protein name	Fold-change	Log2 difference	-Log (P value)
	**T15 min LPS-modified proteins, in PMO**			
MTPN	Myotrophin<br>*Cytoskeletal protein regulating actin filament reorganization. It* ***regulates NF-κB transcription factor activity*** *by promoting p65/p65 and p50/p50 homo-dimerization.*[1] *The homodimers induce the expression of iNOS (inducible NO synthase), which produces NO as a host defense mechanism against pathogens.*[2] *It was up-regulated by oxidative stress in T-lymphocytes.*[3]	1.9	0.9	2.4
C11orf54	Ester hydrolase C11orf54<br>*Unknown function*	1.8	0.9	6.4
MYO1B	Unconventional myosin-Ib<br>*Motor protein regulating the localization of actin-enriched membrane projections,*[4] ***maintains the enterocyte brush-border structure*** *by contributing to bacterial pathogens resistance.*[5]	1.8	0.8	2.3
APRT	Adenine phosphoribosyltransferase<br>*Increases cellular AMP, which in turn* ***activates the anti-inflammatory kinase*** *AMPK (AMP-activated protein kinase).*[6,7]	1.7	0.8	3.0
SOD1	Superoxide dismutase [Cu-Zn]<br>*Highly expressed in the cytoplasm, and other subcellular compartments (nucleus, lysosomes, intermembrane mitochondrial space), it is responsible for the detoxification of superoxide. Activation of AMPK inhibits oxidative stress by stimulating the protein expression of SOD1.*[8]	1.7	0.8	3.2
UBE2L3	Ubiquitin-conjugating enzyme E2 L3<br>*It promotes the* ***ubiquitination of NEMO****, leading to NF-κB activation.*[9] *Its inactivation had anti-inflammatory effects (suppressed MyD88 signaling) in LPS-stimulated macrophages.*[10] *Genetic variations of UBE2L3 were demonstrated in many autoimmune diseases like CD.*[11]	1.7	0.7	3.1
LTA4H	Leukotriene A-4 hydrolase<br>*Catalyzes the final and rate-limiting step in the biosynthesis of pro-inflammatory* ***leukotriene LTB4****, implicated in the* ***protection against invading microorganisms,*** *promoting IL-1β secretion.*[12] *It exerts an anti-inflammatory activity also extracellularly, acting as aminopeptidase to degrade the tripeptide Pro-Gly-Pro (PGP).*[13] *It is involved in multiple chronic diseases, and its overexpression is associated with colon inflammation.*[14]	1.7	0.7	3.0
MIF	Macrophage migration inhibitory factor<br>*Profoundly involved in the pathogenesis of acute and chronic inflammatory disorders (IBD). MIF-deficient mice were resistant in an induced model of colitis*[15] *indicating its potential therapeutic use for IBD patients.*[16] *The signaling pathway MIF–CD74 (MIF receptor)–PKB–ERK is* ***strongly activated during intestinal inflammation*** *and promotes intestinal epithelial cell proliferation, leading to mucosal healing. LPS stimulates MIF mRNA expression and protein levels (20 % increase).*[17]	1.7	0.7	2.5
PSMD2	26S proteasome non-ATPase regulatory subunit 2<br>*Involved in the* ***regulation of inflammatory response through NF-κB activation,*** *and may also interact directly with the TNF receptor, regulating the corresponding signaling pathway. PSMD2 was found up-regulated by LPS.*[17] *Its accumulation, associated with up-regulation of IL-1β, IL-6, and IL-18 mRNA expression, was detected in Crohn's disease.*[18]	1.7	0.7	5.0
PSMB6	Proteasome subunit beta type-6<br>*Its structure and catalytic properties are modified under the influence of the proinflammatory cytokine IFNγ, leading to the formation of the so-called immunoproteasome.*[18]	1.6	0.7	2.7
NUDT5	ADP-sugar pyrophosphatase<br>*Metabolizes ADPR, which is involved in chromatin remodeling and subsequent gene regulation. It is* ***activated during the inflammatory response.***[19]	1.6	0.7	2.9
ST13	Hsc70-interacting protein	1.6	0.7	5.6

RPS21	40S ribosomal protein S21	1.6	0.6	3.4
CUTA	Protein CutA<br>*Membrane protein -Its precise cellular function remains unclear.*	1.5	0.6	4.2
PDCD6	Programmed cell death protein 6<br>*It controls the balance between cell differentiation, proliferation, and apoptosis (1 h-PDCD6 treatment inhibited NF-κB DNA binding activity in human ovarian carcinoma cells[20]) and its alteration is found in various diseases. It was highly expressed in colorectal tumors[21] and the lamina propria of induced colitis mice model treated with Ancylostoma caninum[22]. It was down-regulated in the IECs of the same model and after LPS-induced liver failure.[23]*	1.5	0.6	4.9
ALDH1A1	Retinal dehydrogenase 1<br>*Involved in the irreversible oxidative metabolism of vitamin A retinal metabolite to form all-trans-retinoic acid.[24] It can metabolize a wide range of aldehydes and lipoxygenase-produced ROS, **attenuating oxidative stress.**[24] Its expression was increased in macrophages as part of the **early acute-phase inflammatory response**.[25] Induction of its expression after increased production of retinoic acid contributed to the inflammatory phenotype of Crohn's in IBD patients.[26] A wide range of human cancer stem cells,[27] as well as HT29 expressed high levels of ALDH1A1.[28]*	1.5	0.6	4.2
TSPAN6	Tetraspanin-6	1.5	0.6	2.3
MVP	Major vault protein<br>*MVP is a multi-subunit structure that is involved in nucleo-cytoplasmic transport.*	1.5	0.6	3.6
GOT1	Aspartate aminotransferase, cytoplasmic<br>*Involved in glutamine metabolism, is shown to exert **pro-inflammatory effects** by producing 2-hydroxyglutarate, which blocks the formation of immunosuppressive T-regulatory cells. Inhibition of glutamine metabolism suppressed inflammation in mouse models of acute lung injury.[29]*	1.5	0.6	3.2
CSTB	Cystatin-B<br>*Cystatin-B upon LPS stimulation translocates into mitochondria and protects mitochondrial integrity.[30]*	1.5	0.6	2.7
PSMA5<br>PSMB2<br>PSME1	Proteasome subunit alpha type-5<br>Proteasome subunit beta type-2<br>Proteasome activator complex subunit 1<br>*These proteasome subunits also constitute the **immunoproteasome** (UPS), involved in the regulation of inflammatory responses, as in the activation of NF-κB which further regulates the expression of pro-inflammatory cytokines such as TNF-α, IL-β, IL-8, adhesion molecules (ICAM-1, VCAM-1, P-selectin) and prostaglandins and nitric oxide.[31]*	1.5<br>1.5<br>1.4	0.6<br>0.5<br>0.5	3.1<br>5.0<br>3.3
MDH1	Malate dehydrogenase, cytoplasmic<br>*Enzyme of the TCA cycle, important in mitochondrial NADH supply for oxidative phosphorylation, is **overexpressed** in dendritic cells **after TLR stimulation**, perturbing TCA cycle metabolism and **enhancing mtROS production**.[32]*	1.4	0.5	4.0
NQO1	NAD(P)H dehydrogenase [quinone] 1<br>*It reduces quinines and quinones through NADH/NAD^+ transformation, **protecting cellular membranes against oxidative damage**. Its elevated levels were detected in the lungs as an adaptive cellular response to counteract oxidative stress and inflammation-related tissue injury.[33] The colons of NQO1-KO mice showed high levels of ROS and histone deacetylase (HDAC) activity, which affected transcriptional regulation.[34] NQO1 and SOD2 polymorphisms can influence the age of onset of UC.[35] Its expression was **1.4-fold increased by LPS via Nrf2 at 30 min in human monocytes**.[36] It induced IκB-ζ ubiquitination (indispensable for TLR-mediated expression of IL-6, IL-12), reached a maximum level within 1–2h, and rapidly declined in WT macrophages. It directly suppressed LPS-TLR4 signaling and subsequent cytokine production.*	1.4	0.5	4.8
NIT2	Omega-amidase NIT2	1.4	0.5	4.2
TFG	Protein TFG	1.4	0.5	2.7
CNN2	Calponin-2	1.4	0.5	4.2
ARPC4	Actin-related protein 2/3 complex subunit 4	1.4	0.5	3.3

	Major protein complex in actin polymerization enhances the rate of actin polymerization after Mycobacterium tuberculosis infection.[37]			
SERPINB1	Leukocyte elastase inhibitor	1.4	0.5	2.9
SERPINB6	Serpin B6	1.3	0.4	4.3
NUTF2	Nuclear transport factor 2<br>*NUTF2 regulates the efficiency of protein transport between nucleus and cytoplasm.*[38]	1.3	0.4	3.5
TKT	Transketolase<br>*Transketolase is the rate-controlling enzyme in the non-oxidative branch of the pentose phosphate pathway (PPP), connects PPP to glycolysis, feeding excess sugar phosphates into the main carbohydrate metabolic pathways and bridges the oxidative part of the PPP by NADPH production. After oxidation stress, Nrf2 increases transcription of TKT.*	1.3	0.4	4.0
 GPI<br>ALDOA<br>LDHB	Glycolysis<br>Glucose-6-phosphate isomerase<br>Fructose-bisphosphate aldolase A<br>L-lactate dehydrogenase B chain	 1.3<br>1.3<br>1.3	 0.4<br>0.4<br>0.4	 3.4<br>3.7<br>3.7
NME2	Nucleoside diphosphate kinase B	1.3	0.4	3.9
CMPK1	UMP-CMP kinase	1.3	0.4	4.1
OAT	Ornithine aminotransferase, mitochondrial	1.3	0.4	3.8
YBX1	Nuclease-sensitive element-binding protein 1	1.3	0.4	5.4
COX6B1	Cytochrome c oxidase subunit 6B1	0.6	-0.7	3.3
SLC35B2	Adenosine 3-phospho 5-phosphosulfate transporter 1	0.7	-0.5	4.2
PCNA	Proliferating cell nuclear antigen	0.7	-0.4	2.8
H2AFY	Core histone macro-H2A.1	0.7	-0.4	2.8
**T15 min LPS Mn1-up-leveled proteins, in PMO**				
RPS26	40S ribosomal protein S26	1.8	0.9	3.5
SAR1B	GTP-binding protein SAR1b	1.5	0.6	4.8
TARS	Threonine--tRNA ligase, cytoplasmic	1.4	0.4	5.6
SKP1	S-phase kinase-associated protein 1	1.3	0.4	6.1
**T15 min LPS Mn1-modified proteins, in Cytosol**				
UGGT1	UDP-glucose:glycoprotein glucosyltransferase 1	1.3	0.4	5.0
UQCRC1	Cytochrome b-c1 complex subunit 1, mitochondrial	0.4	-1.2	2.3
GPRC5A	Retinoic acid-induced protein 3	0.5	-0.9	2.3
HNRNPH3	Heterogeneous nuclear ribonucleoprotein H3	0.6	-0.8	4.7
EPHA2	Ephrin type-A receptor 2	0.6	-0.8	2.5
OAS3	2-5-oligoadenylate synthase 3	0.6	-0.7	2.9
GLUD1	Glutamate dehydrogenase 1, mitochondrial	0.7	-0.6	3.2
HTATSF1	HIV Tat-specific factor 1	0.7	-0.6	2.8
ACSL3	Long-chain-fatty-acid--CoA ligase 3	0.7	-0.5	3.6
GOT2	Aspartate aminotransferase, mitochondrial	0.8	-0.4	5.3

B.2. LPS and LPS Mn1 effects on the protein oxidation level, after 15-min incubation

Table S.2.6 Proteins with increasing oxidation level after 15-min LPS-stimulation of HT29-MD2 cells, in the PMO and cytosolic fraction, were compared to the corresponding LPS **Mn1** conditions. The oxidation status of a protein was measured by the increased *S-Ox peptides* (x/z)/T0 >1 and by the decreased *S-Red peptides* (y/z)/T0 <1. Cysteines are shown in bold red in the peptide sequence. Proteins in bold blue are discussed in the article. The PMO fraction is highlighted in blue.

15-min incubation									
**Protein**	**Peptides with -Cys**	**Fold-Change S-Ox in PMO**		**Fold-Change S-Ox in Cyto**		**Fold-Change S-Red in PMO**		**Fold-Change S-Red in Cyto**	
		**LPS**	**LPS Mn1**	**LPS**	**LPS Mn1**	**LPS**	**LPS Mn1**	**LPS**	**LPS Mn1**
ACTG1	**Cys257**PEALFQPSFLGMES**Cys272**GIHETTFNSIMK					0.9	1.0		
ACTN4	**Cys352**QLEINFNTLQTK							0.7	0.6
ADPGK	VAGTQA**Cys415**ATETIDTSR	1.1	1.1						
ALDOA	YASI**Cys178**QQNGIVPIVEPEILPDGDHDLKR			1.2	1.0				
	YASI**Cys178**QQNGIVPIVEPEILPDGDHDLK			1.1	1.0				
ANXA1	MYGISL**Cys324**QAILDETK							0.9	1.0
ANXA2	GLGTDEDSLIEII**Cys133**SR							0.9	1.0
	GDLENAFLNLVQ**Cys262**IQNKPLYFADR							0.9	1.0
APMAP	TRDDEPV**Cys149**GRPLGIR	2.9	1.3						
	DDEPV**Cys149**GRPLGIR	2.5	1.0						
BSG	SSEHINEGETAMLV**Cys242**K *Implications in immunoglobulin superfamily homophilic adhesion after oxidation*			1.2					
CCDC58	N**Cys74**IAQTSAVVK			1.1	1.0				
CCT2	SLHDAL**Cys395**VLAQTVK							0.9	0.8
CD46	GSVAIWSGKPPI**Cys157**EK	2.0	0.9						
CD59	LRENELTYY**Cys88Cys89**K			1.1					
CLIC1	EEFAST**Cys223**PDDEEIELAYEQVAK							0.9	1.0
CSRP1	GLESTTLADKDGEIY**Cys167**K *Cys167 oxidation of Csrp proteins may cause structural changes and defects.*							0.9	
CTSB	GLVSGGLYESHVG**Cys179**R			1.1	1.0				
DDX39B	N**Cys165**PHIVVGTPGR *RNA helicase which is involved in almost all the RNA processes such as mRNA capping, splicing, and poly-adenylating to be the mature mRNA and export.*					0.9	1.0		
DSTN	HE**Cys135**QANGPEDLNR					0.9	1.0		
ECHS1	EMQNLSFQD**Cys111**YSSK					0.9	1.0		
EEF1A1	SGDAAIVDMVPGKPM**Cys411**VESFSDYPPLGR					0.9	0.9		
EIF5A	YEDI**Cys73**PSTHNMDVPNIK							0.9	1.0
EPCAM	RTDKDTEIT**Cys135**SER	3.5	0.9						
EPRS1	EAP**Cys697**VLIYIPDGHTK			1.1	1.1				
FASN	AIN**Cys1448**ATSGVVGLVN**Cys1459**LR							0.9	0.9
FLNB	NGS**Cys1095**GVSYIAQEPGNYEVSIK							0.9	1.0
GALNT3	DLGPDTRPPE**Cys173**IEQK	1.3	0.9						

GDI2	TYDATTHFETT**Cys414**DDIK							0.9	0.9
GLUD1	**Cys327**IAVGESDGSIWNPDGIDPK					0.9	1.0		
GNB1	ELAGHTGYLS**Cys148Cys149**R					0.9	1.0		
GOT2	EYLPIGGLAEF**Cys106**K					0.9	0.9		
	NLDKEYLPIGGLAEF**Cys106**K					0.9	1.1		
GPI	MIP**Cys404**DFLIPVQTQHPIR			1.1	1.0				
HNRNPD	GF**Cys226**FITFKEEEPVKK					0.9			
HNRNPH1	GLPWS**Cys22**SADEVQR							0.9	0.9
HNRNPL	ASLNGADIYSG**Cys260Cys261**TLK					0.8			
HNRNPUL 1	AIVI**Cys532**PTDEDLKDR			1.1	1.2				
HSP90AB1	RGFEVVYMTEPIDEY**Cys521**VQQL K					0.8	1.1		
	LVSSP**Cys589Cys590**IVTSTYGWTA NMER<br>Cys590 –S-Nitrocysteine					0.8	1.0		
	GFEVVYMTEPIDEY**Cys521**VQQLK							0.9	0.9
HSPA1B	FEEL**Cys306**SDLFR							0.9	1.0
	AAAIGIDLGTTYS**Cys17**VGVFQHG K							0.9	1.0
HSPA8	GPAVGIDLGTTYS**Cys17**VGVFQHG K							0.9	0.9
	**Cys574**NEIINWLDK							0.9	0.9
HSPH1	KPVTD**Cys140**VISVPSFFTDAER							0.9	0.8
ITGA6	IEDDMDGGDWSF**Cys175**DGR	1.1	1.1						
ITGB1	DKLPQPVQPDPVSH**Cys691**K	1.8	1.0	1.4	1.0				
ITGB5	NG**Cys75**GGEIESPASSFHVLR	1.1	1.0						
KLK6	EKPGVYTNV**Cys231**R	2.5	1.4	1.2	0.9				
	DS**Cys193**QGDSGGPLV**Cys203**GDH LR	1.4	1.1	1.1	0.8				
LGALS3BP	STSSFP**Cys561**PAGHFNGFR							0.8	0.6
MAPRE1	NIELI**Cys228**QENEGENDPVLQR							0.8	1.0
NUTF2	NINDAWV**Cys114**TNDMFR			1.1	1.1				
PDXK	LVYV**Cys112**DPVLGDK			1.1					
PLEC	**Cys3008**ITDPQTGLCLLPLK							0.9	1.0
PPIA	IIPGFM**Cys62**QGGDFTR							0.9	1.1
PPP1CA	TFTD**Cys155**FN**Cys158**LPIAAIVDEK					0.9	1.0		
**PRDX1**	HGEV**Cys173**PAGWKPGSDTIKPDV QK	2.4	1.1	1.1	0.9				
	LN**Cys71**QVIGASVDSHF**Cys83**HLA WVNTPK							0.9	0.9
**PRDX2**	LVQAFQYTDEHGEV**Cys172**PAGW KPGSDTIKPNVDDSK			1.2	1.0				
**PRDX3**	AFQYVETHGEV**Cys229**PANWTPDS PTIKPSPAASK	1.6	0.9						
**PRDX4**	TREEE**Cys51**HFYAGGQVYPGEASR	2.7	1.1						
**PRDX6**	DINAYN**Cys91**EEPTEKLPFPIIDDR			1.1	1.0				
PSME1	KGEDEDKGPP**Cys101**GPVN**Cys106** NEK							0.9	0.9
RACK1	HLYTLDGGDIINAL**Cys240**FSPNR					0.9	0.8	0.9	0.9
RBBP7	HPAKPDPSGE**Cys166**NPDLR							0.9	1.1
RPL14	**Cys54**MQLTDFILK							0.9	0.8
RPL30	V**Cys92**TLAIIDPGDSDIIR					0.9			
	TGVHHYSGNNIELGTA**Cys85**GK							0.9	0.9
RPL5	VGLTNYAAAY**Cys100**TGLLLAR							0.9	0.9

RPS5	TIAE**Cys172**LADELINAAK					0.9	1.0	0.9	0.9
RPS3A	N**Cys96**LTNFHGMDLTR							0.9	0.9
SDHA	ACALSIEESCys475RPGDK					0.9	1.0		
SEC13	DVAWAPSIGLPTSTIASCys234SQD GR							0.9	1.0
SNRPD2	NNTQVLIN**Cys46**R			1.1	0.9				
**SOD1**	GLTEGLHGFHVHEFGDNTAG**Cys58** TSAGPHFNPLSR			1.2	1.2				
SPINT2	SEEA**Cys179**MLR	1.3	1.2						
STIM1	IDKPL**Cys56**HSEDEK	1.6	1.0						
TGM2	TVSYNGILGPE**Cys524**GTK							0.9	1.1
	VVSGMVN**Cys230**NDDQGVLLGR **S230-S370** *The disulfide bond formation inactivates the calcium-dependent acyltransferase activity*							0.9	1.0
	YRD**Cys554**LTESNLIK							0.9	1.0
TM9SF4	TQLPYEYYSLPF**Cys64**QPSK			1.1	1.1				
TPI1	D**Cys87**GATWVVLGHSER	1.3	1.3						
	IAVAAQN**Cys67**YK	1.2	1.1						
	VAHALAEGLGVIA**Cys127**IGEKLDE R							0.9	1.0
	IIYGGSVTGAT**Cys218**K							0.9	1.0
TUBA1B	AV**Cys376**MLSNTTAIAEAWAR					0.9	1.1		
**TXNDC5**	VD**Cys247**TQHYEL**Cys254**SGNQVR	1.1	0.9						
UQCRC1	AVELLGDIVQN**Cys154**SLEDSQIEK					0.9	1.0		
	NALVSHLDGTTPV**Cys410**EDIGR					0.9	1.0		
VCP	QAAP**Cys241**VLFFDELDSIAK							0.9	1.0
YWHAQ	YLAEVA**Cys134**GDDRK							0.9	0.9
YWHAZ	DI**Cys17**NDVLSLLEK					0.9	1.0	0.9	0.9

C. 30 minutes

C.1. LPS and LPS Mn1 effects on the proteome level, after 30-min incubation

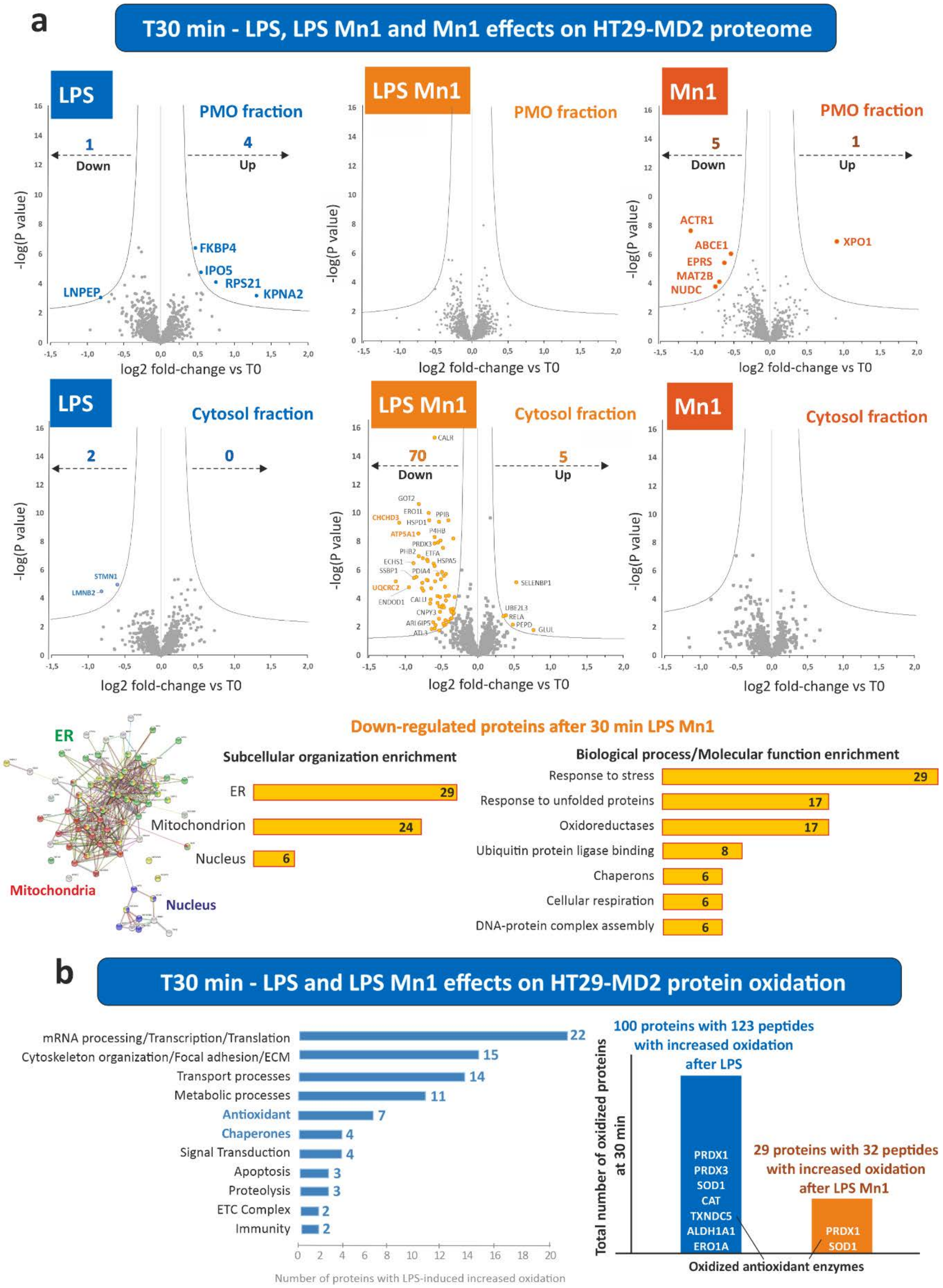

Figure S.2.5 Comparison of LPS, LPS Mn1 and Mn1 (30-min incubation). Effects of 30-min incubation of LPS, LPS Mn1, and Mn1 alone, on the proteome and Cys oxidation level (vs. T0 sample). a LPS, LPS **Mn1,** and **Mn1** effects were plotted in volcano plots giving the t-test p-value (in log10 scale y-axis; significant if p-value < 0.01, 1 % error) against the fold-change between two chosen conditions (in log2 scale x-axis). The level-modified proteins are indicated with blue circles (LPS), orange circles (LPS **Mn1**), and dark orange (**Mn1**). In a histogram, the 70 LPS **Mn1** down-leveled proteins were classified according to their subcellular location and biological function. **b** LPS and LPS

Mn1 effects on protein oxidation status in both PMO and cytosolic fractions. Left: LPS-oxidized proteins reported according to their biological function, right: LPS-oxidized proteins in the LPS **Mn1** sample. The LPS condition is shown in blue, and the LPS **Mn1** condition is shown in orange.

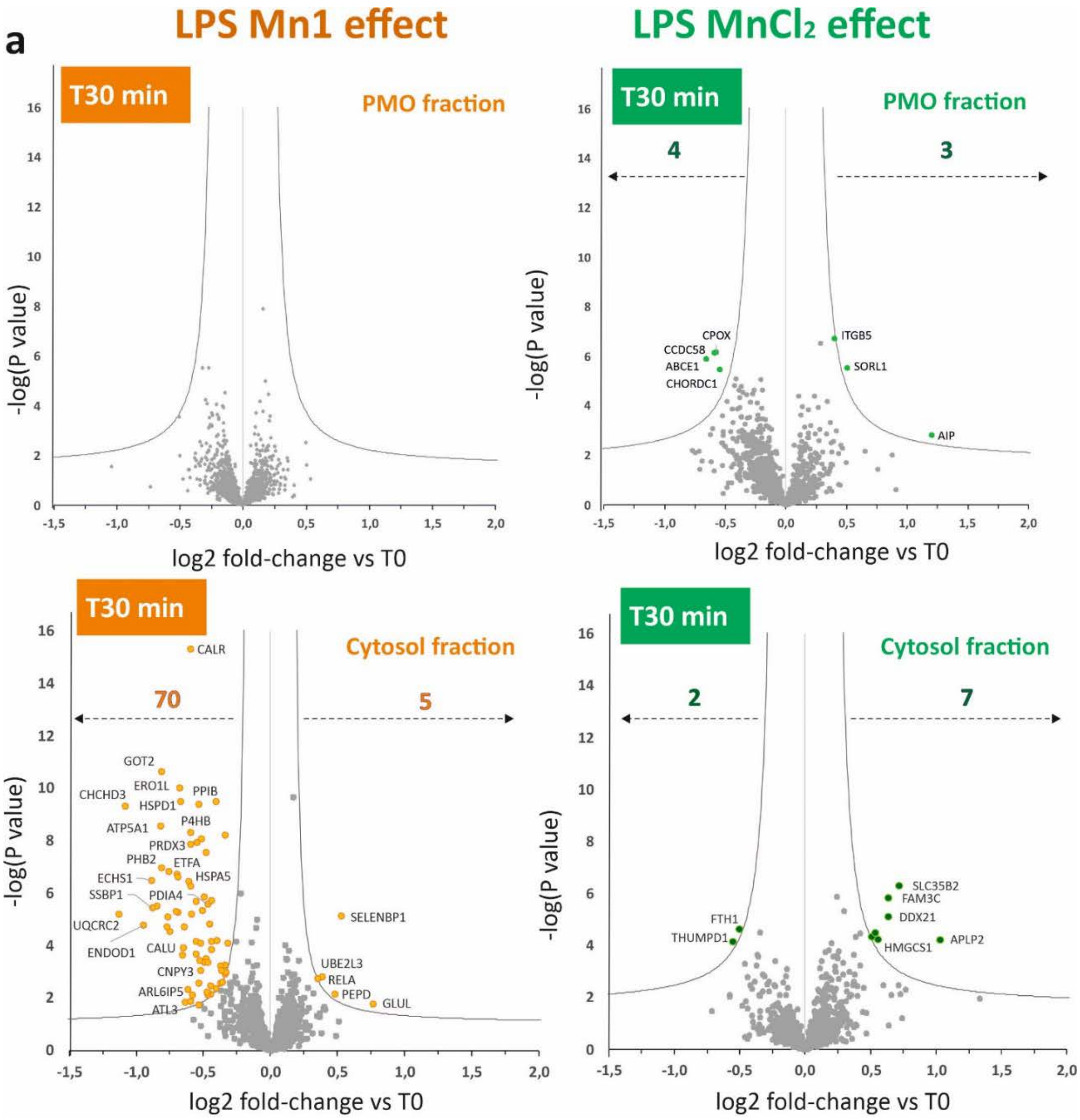

Figure S.2.6 Comparison of LPS Mn1 and LPS MnCl₂, 30-min incubation, proteins levels (vs. T0 sample) in the PMO and cytosolic fractions of HT29-MD2 cells. LPS **Mn1,** and **LPS $MnCl_2$** effects were plotted in volcano plots giving the t-test p-value (in log10 scale y-axis; significant if p-value < 0.01, 1 % error) against the fold-change between two chosen conditions (in log2 scale x-axis). The up- and down- leveled proteins in LPS **Mn1** are indicated with orange circles, and in **LPS $MnCl_2$ they** are indicated with green circles. The LPS **Mn1** condition is shown in orange, and the LPS $MnCl_2$ condition is shown in green.

Table S.2.7 Up- and down-leveled proteins after 30-min LPS, LPS **Mn1**, LPS $MnCl_2$, and **Mn1** alone stimulation of HT29-MD2 cells, vs. T0 (non-incubated or basal sample). The headline of the LPS condition is shown in blue, the LPS **Mn1** condition is shown in orange, **Mn1** condition is shown in dark orange, LPS $MnCl_2$ condition is shown in green. Down-leveled proteins are highlighted in yellow.

Protein	Protein name	Fold-change	Log2 difference	-Log (P value)
**T30 min LPS-modified proteins, in PMO**				
KPNA2	Importin subunit alpha-1	2.5	1.3	3.2
RPS21	40S ribosomal protein S21	1.7	0.8	4.1
IPO5	Importin-5	1.5	0.5	4.8
FKBP4	Peptidyl-prolyl cis-trans isomerase FKBP4	1.4	0.5	6.4
LNPEP	Leucyl-cystinyl aminopeptidase	0.6	-0.8	3.1
**T30 min LPS Mn1- down-leveled proteins, in cytosol**				
UQCRC2	Cytochrome b-c1 complex subunit 2, mitochondrial	0.5	-1.1	5.2
CHCHD3	MICOS complex subunit MIC19	0.5	-1.1	9.3
ENDOD1	Endonuclease domain-containing 1 protein	0.5	-0.9	4.8
ECHS1	Enoyl-CoA hydratase, mitochondrial	0.5	-0.9	6.5
SSBP1	Single-stranded DNA-binding protein, mitochondrial	0.5	-0.9	5.4
CS	Citrate synthase, mitochondrial	0.6	-0.8	5.5
ATP5A1	ATP synthase subunit alpha, mitochondrial	0.6	-0.8	8.5
PHB2	Prohibitin-2	0.6	-0.8	7.0
GOT2	Aspartate aminotransferase, mitochondrial	0.6	-0.8	10.6
C1QBP	Complement component 1 Q -binding protein, mitochondrial	0.6	-0.8	4.7
STIM1	Stromal interaction molecule 1	0.6	-0.8	5.1
EPHX1	Epoxide hydrolase 1	0.6	-0.8	6.8
LMAN1	Protein ERGIC-53	0.6	-0.8	4.5
ESYT1	Extended synaptotagmin-1	0.6	-0.7	5.3
ETFA	Electron transfer flavoprotein subunit alpha, mitochondrial	0.6	-0.7	6.7
HSPA9	Stress-70 protein, mitochondrial	0.6	-0.7	6.6
FKBP2	Peptidyl-prolyl cis-trans isomerase FKBP2	0.6	-0.7	5.3
ERO1L	ERO1-like protein alpha	0.6	-0.7	10.0
HSPD1	60 kDa heat shock protein, mitochondrial	0.6	-0.7	9.5
HSD17B10	3-hydroxyacyl-CoA dehydrogenase type-2	0.6	-0.7	3.6
CALU	Calumenin	0.6	-0.7	3.9
SERPINH1	Serpin H1	0.6	-0.6	4.7
TFRC	Transferrin receptor protein 1	0.6	-0.6	1.8
ARL6IP5	PRA1 family protein 3	0.7	-0.6	2.3
HSPA5	78 kDa glucose-regulated protein	0.7	-0.6	6.4
HSPE1	10 kDa heat shock protein, mitochondrial	0.7	-0.6	6.3
PRDX3	Thioredoxin-dependent peroxide reductase, mitochondrial	0.7	-0.6	7.8
ATL3	Atlastin-3	0.7	-0.6	1.9
P4HB	Protein disulfide-isomerase	0.7	-0.6	8.3
CALR	Calreticulin	0.7	-0.6	15.3
CANX	Calnexin	0.7	-0.6	5.2
MT-CO2	Cytochrome c oxidase subunit 2	0.7	-0.6	2.1
ERP44	Endoplasmic reticulum resident protein 44	0.7	-0.6	5.7
TUFM	Elongation factor Tu, mitochondrial	0.7	-0.6	3.7
PLOD3	Procollagen-lysine,2-oxoglutarate 5-dioxygenase 3	0.7	-0.6	4.2
PDIA3	Protein disulfide-isomerase A3	0.7	-0.5	7.9
ETFB	Electron transfer flavoprotein subunit beta	0.7	-0.5	2.6
PPIB	Peptidyl-prolyl cis-trans isomerase B	0.7	-0.5	9.4
SLC3A2	4F2 cell-surface antigen heavy chain	0.7	-0.5	1.7
LMNB1	Lamin-B1	0.7	-0.5	3.4
CNPY3	Protein canopy homolog 3	0.7	-0.5	3.0
SLC25A5	ADP/ATP translocase 2	0.7	-0.5	4.1
MDH2	Malate dehydrogenase, mitochondrial	0.7	-0.5	8.1
ADPGK	ADP-dependent glucokinase	0.7	-0.5	5.3
H2AFY	Core histone macro-H2A.1	0.7	-0.5	3.3
GANAB	Neutral alpha-glucosidase AB	0.7	-0.5	5.9
TXNDC5	Thioredoxin domain-containing protein 5	0.7	-0.5	7.6
SPINT2	Kunitz-type protease inhibitor 2	0.7	-0.5	3.5
KLK6	Kallikrein-6	0.7	-0.5	2.2
RCN1	Reticulocalbin-1	0.7	-0.5	2.1

PDIA4	Protein disulfide-isomerase A4	0.7	-0.5	5.6
MCM6	DNA replication licensing factor MCM6	0.7	-0.5	3.4
PDIA6	Protein disulfide-isomerase A6	0.7	-0.5	4.8
HAT1	Histone acetyltransferase type B catalytic subunit	0.7	-0.5	2.1
BAG2	BAG family molecular chaperone regulator 2	0.7	-0.4	2.5
MANF	Mesencephalic astrocyte-derived neurotrophic factor	0.7	-0.4	4.2
RPN1	Dolichyl-diphosphooligosaccharide--protein glycosyltransferase	0.7	-0.4	3.8
HIST1H2BL	Histone H2B type 1-L	0.7	-0.4	5.7
HIST1H2AJ	Histone H2A type 1-J	0.8	-0.4	9.5
CEACAM1	Carcinoembryonic antigen-related cell adhesion molecule 1	0.8	-0.4	2.3
HSP90B1	Endoplasmin	0.8	-0.4	4.2
M6PR	Cation-dependent mannose-6-phosphate receptor	0.8	-0.4	3.2
NPM3	Nucleoplasmin-3	0.8	-0.4	2.5
CTSC	Dipeptidyl peptidase 1	0.8	-0.4	3.0
TMPO	Thymopoietin	0.8	-0.4	2.6
HIST1H1C	Histone H1.2	0.8	-0.4	3.2
ATP5B	ATP synthase subunit beta, mitochondrial	0.8	-0.4	2.9
HIST1H4A	Histone H4	0.8	-0.4	8.2
DDX5	Probable ATP-dependent RNA helicase	0.8	-0.4	3.0
PABPC4	Polyadenylate-binding protein	0.8	-0.4	4.1
**T30 min Mn1-modified proteins, in PMO**				
XPO1	Exportin-1	1.9	0.9	7.0
ACTR2	Actin-related protein 2	0.5	-1.1	7.7
EPRS	Bifunctional glutamate/proline - tRNA ligase	0.6	-0.8	5.5
NUDC	Nuclear migration protein NudC	0.6	-0.7	3.9
MAT2B	Methionine adenosyltransferase 2 subunit beta	0.6	-0.7	4.1
ABCE1	ATP-binding cassette sub-family E member 1	0.7	-0.5	6.1
**T30 min LPS $MnCl_2$-modified proteins, in PMO**				
AIP	AH receptor-interacting protein	2.3	1.2	2.8
SORL1	Sortilin-related receptor	1.4	0.5	5.5
ITGB5	Integrin beta-5	1.3	0.4	6.7
ABCE1	ATP-binding cassette sub-family E member 1	0.6	-0.7	5.9
CCDC58	Coiled-coil domain-containing protein 58	0.7	-0.6	6.1
CPOX	Oxygen-dependent coproporphyrinogen-III oxidase, mitochondrial	0.7	-0.6	6.2
CHORDC1	Cysteine and histidine-rich domain-containing protein 1	0.7	-0.5	5.5
**T30 min LPS $MnCl_2$-modified proteins, in cytosol**				
APLP2	Amyloid-like protein 2	2.0	1.0	4.2
SLC35B2	Adenosine 3-phospho 5-phosphosulfate transporter 1	1.7	0.7	6.3
DDX21	Nucleolar RNA helicase 2	1.6	0.6	5.1
FAM3C	Protein FAM3C	1.6	0.6	5.8
HMGCS1	Hydroxymethylglutaryl-CoA synthase, cytoplasmic	1.5	0.6	4.2
ST14	Suppressor of tumorigenicity 14 protein	1.4	0.5	4.4
RAB2A	Ras-related protein Rab-2A	1.4	0.5	4.3
THUMPD1	THUMP domain-containing protein 1	0.7	-0.6	4.1
FTH1	Ferritin heavy chain	0.7	-0.5	4.6

C.2. LPS and LPS Mn1 effects on the proteome level, after 30-min incubation

Table S.2.8 Proteins with increasing oxidation level after 30-min LPS stimulation of HT29-MD2 cells, in the PMO and cytosolic fractions, were compared to the corresponding LPS **Mn1** and LPS $MnCl_2$ conditions. The oxidation status of a protein was measured by the increased *S-Ox peptides* (x/z)/T0 >1 and decreased *S-Red peptides* (y/z)/T0 <1. Cysteins are shown in bold red in the peptide sequence. The PMO fractions are highlighted in blue.

30-min incubation													
Protein	Peptides with -Cys	Fod-Change S-Ox in PMO			Fold-Change S-Ox in Cyto			Fold-Change S-Red in PMO			Fold-Change S-Red in Cyto		
		LPS	LPS Mn1	LPS $MnCl_2$	LPS	LPS Mn1	LPS $MnCl_2$	LPS	LPS Mn1	LPS $MnCl_2$	LPS	LPS Mn1	LPS $MnCl_2$
ACTN4	**Cys352**QLEINFNTLQTK							0.7	0.6				
	A**Cys794**LISLGYDVEND RQGEAEFNR							0.9	1.0				
ALDH1A1	KFPVFNPATEEEL**Cys50** QVEEGDKEDVDK										0.6		
	**Cys421**DDSVGYFVEP**Cys432**IVESK							0.9	1.0	1.2			
ANXA1	**Cys270**ATSKPAFFAEK							0.9					
ANXA2	GDLENAFLNLVQ**Cys262** IQNKPLYFADR							0.9					
APMAP	TRDDEPV**Cys149**GRPLGIR	1.1	1.1	1.2									
	DDEPV**Cys149**GRPLGIR	1.1	0.9										
BSG	SSEHINEGETAMLV**Cys242**K *BSG oxidation is implicated in immunoglobulin superfamily homophilic adhesion.*	1.1		1.0									
CAP1	INSITVDN**Cys375**K	1.1		1.0	1.1	1.1	1.0						
CAPNS1	YSDESGNMDFDNFIS**Cys232**LVR										0.8	1.0	0.9
CAPZB	DETVSD**Cys206**SPHIANIGR							0.9	0.9	0.8	0.9	1.0	1.0
CAT	LVNANGEAVY**Cys232**K				1.1	1.0	1.2						
CD46	GSVAIWSGKPPI**Cys157**EK	1.1	1.0	0.9									
CD55	IPGEKDSVI**Cys81**LK	2.2		0.8									
	EIY**Cys225**PAPPQIDNGII QGER	1.2	0.9	0.9	1.1	0.9							
CD59	LRENELTYY**Cys88Cys89**K	1.4	1.1	1.2									
	FEH**Cys70**NFNDVTTR	1.1	0.9	1.1	1.1	1.0	1.0						
CFL1	AVLF**Cys39**LSEDKK										0.9	0.9	1.0
CLIC1	EEFAST**Cys223**PDDEEIE LAYEQVAK										0.9	1.0	0.9
CLTC	LPVVIGGLLDVD**Cys824** SEDVIK							0.9			0.9		
	YESLEL**Cys436**RPVLQQGR										0.9	0.9	
CNBP	**Cys141**GETGHVAIN**Cys151**SK							0.9					
	GFQFVSSSLPDI**Cys55**YR							0.9					
COPG1	AL**Cys129**QITDSTMLQAIER							0.8					
CTSD	AIGAVPLIQGEYMIP**Cys329**EK							0.8	0.9	0.8	0.7		1.0
DAZAP1	IFVGGIPHN**Cys124**GETELR							0.9					
DDX39B	N**Cys165**PHIVVGTPGR							0.9	1.0	0.9			

	RNA helicase which is involved in almost all the RNA processes such as mRNA capping, splicing, and poly-adenylating to be the mature mRNA and export.												
DPYSL2	FQLTD**Cys284**QIYEVLSVIR							0.9					
DSTN	HE**Cys135**QANGPEDLNR										0.9	0.9	0.9
ECHS1	I**Cys213**PVETLVEEAIQ**Cys225**AEK										0.9		
	EMQNLSFQD**Cys111**YSSK										0.9		
EPCAM	RTDKDTEIT**Cys135**SER	1.1		0.8									
ERO1A	YSEEANNLIEE**Cys131**EQAER	1.1		1.0									
ETFB	HSMNPF**Cys42**EIAVEEAVR							0.8	1.1	0.9	0.8		
	EVIAVS**Cys66**GPAQ**Cys71**QETIR										0.9		
FASN	F**Cys1118**FTPHTEEG**Cys1127**LSER							0.9					
FUBP1	**Cys328**QHAAEIITDLLR							0.9	1.0				
GALNT7	SEVLHQVFISN**Cys640**DSSK	1.1		0.8									
GDI2	TYDATTHFETT**Cys414**DDIK										0.9	1.0	0.9
GLUD1	**Cys327**IAVGESDGSIWNPDGIDPK							0.9	1.0	0.9			
GNAI2	EIYTHFT**Cys326**ATDTK										0.8		
GNB1	A**Cys25**ADATLSQITNNIDPVGR							0.9	1.2				
GNB2	ELPGHTGYLS**Cys148Cys149**R										0.9		
GOT2	VGAFTMV**Cys295**K	1.1	1.0	0.9									
GPD2	**Cys285**VINATGPFTDSVR							0.9	1.1				
HNRNPA3	YHTINGHN**Cys196**EVK							0.9	1.0	1.0			
HNRNPD	GF**Cys226**FITFKEEEPVKK							0.9	1.0				
HNRNPF	GLPWS**Cys22**SVEDVQNFLSD**Cys34**TIHDGAAGVHFIYTR							0.9					
HNRNPH1	YGDGGSTFQSTTGH**Cys290**VHMR							0.9					
	DLNY**Cys266**FSGMSDHR							0.9		0.9			
HNRNPL	ASLNGADIYSG**Cys260Cys261**TLK										0.9	0.8	1.0
HNRNPUL1	AIVI**Cys532**PTDEDLKDR	1.1		1.1	1.1	1.1	1.1						
HSD17B4	SNIH**Cys189**NTIAPNAGSR							0.9	1.0	1.0			
HSP90AB1	GFEVVYMTEPIDEY**Cys521**VQQLK							0.9		0.7	0.9	0.9	0.9
	VFIMDS**Cys366**DELIPEYLNFIR							0.9	1.0		0.9	0.9	1.0
HSPA1B	**Cys574**QEVISWLDANTLAEK							0.9	1.1		0.9	1.1	1.1
	ARFEEL**Cys306**SDLFR							0.9	1.0	1.0	0.9	1.0	1.4
	FEEL**Cys306**SDLFR							0.9	1.0	0.8	0.9	0.9	
	AAAIGIDLGTTYS**Cys17**VGVFQHGK										0.9	1.0	
HSPA8	GPAVGIDLGTTYS**Cys17**VGVFQHGK							0.9					

	**Cys574**NEIINWLDK							0.9	1.0	1.0			
HSPA9	MEEFKDQLPADE**Cys608**NK							0.9	0.8	0.8			
HSPH1	SVLDAAQIVGLN**Cys167**LR							0.8	0.8	0.9	0.9	0.9	1.0
	KPVTD**Cys140**VISVPSFFTDAER							0.9		0.9			
IPO7	GIDQ**Cys757**IPLFVEAALER										0.9	1.0	1.1
ITGA6	IEDDMDGGDWSF**Cys175**DGR	1.2											
ITGB1	DKLPQPVQPDPVSH**Cys691**K	1.1	0.9	1.0									
ITGB5	NG**Cys75**GGEIESPASSFHVLR	1.1	1.1	1.2									
LMAN1	NNPAIVIIGNNGQIHYDHQNDGASQALAS**Cys190**QR	1.1		1.0									
LMAN2	N**Cys239**IDITGVR	1.1		1.0									
LUC7L2	SHLLN**Cys43Cys44**PHDVLSGTR							0.9					
NONO	**Cys218**SEGSFLLTTFPR							0.9	1.0				
NUTF2	NINDAWV**Cys114**TNDMFR	1.1		1.0	1.1	1.2	1.0						
PCNA	DLSHIGDAVVIS**Cys162**AK							0.9		1.2			
PKM	NTGII**Cys49**TIGPASR							0.9	0.9		0.9	0.9	0.9
PPIB	DKPLKDVIIAD**Cys202**GK	1.3		0.9	1.1	0.6	1.0						
PPP1CA	TFTD**Cys155**FN**Cys158**LPIAAIVDEK							0.9					
PRDX1	LN**Cys71**QVIGASVDSHF**Cys83**HLAWVNTPK							0.9		0.7	0.9	1.0	0.9
	HGEV**Cys173**PAGWKPGSDTIKPDVQK										0.8	1.1	1.2
PRDX3	AFQYVETHGEV**Cys229**PANWTPDSPTIKPSPAASK	1.1		0.9									
RACK1	HLYTLDGGDIINAL**Cys240**FSPNR										0.9	0.9	1.0
RBBP7	HPAKPDPSGE**Cys166**NPDLR							0.9					
RPL10A	FSV**Cys66**VLGDQQH**Cys74**DEAK							0.9			0.9	0.9	0.9
RPL14	**Cys54**MQLTDFILK										0.8	0.8	0.9
RPL23	ISLGLPVGAVIN**Cys28**ADNTGAK							0.9	1.1	1.0			
RPL5	VGLTNYAAAY**Cys100**TGLLLAR										0.9	0.9	0.9
RPLP0	AGAIAP**Cys80**EVTVPAQNTGLGPEK							0.9	0.9	0.9			
RPS5	TIAE**Cys172**LADELINAAK							0.9	1.0	0.9			
RPS11	NMSVHLSP**Cys116**FR							0.8					
RPS12	LVEAL**Cys69**AEHQINLIK							0.9	0.9	0.8			
SDCBP	FGDQVLQINGEN**Cys166**AGWSSDK							0.9	0.9	1.1			
	NGLLTEHNI**Cys239**EINGQNVIGLK							0.9	0.9	1.2	0.8		
SDHA	HVNGQDQIVPGLYA**Cys430**GEAA**Cys435**ASVHGANR							0.9					
SEC13	DVAWAPSIGLPTSTIAS**Cys234**SQDGR										0.9		

SLC25A5	KGTDIMYTGTLD**Cys257**WR							0.9	1.0				
SND1	ET**Cys549**LITFLLAGIE**Cys560**PR							0.9					
SNRPD3	LIEAEDNMN**Cys41**QMSNITVTYR	1.1		1.0	1.1	1.0	1.0						
SOD1	GLTEGLHGFHVHEFGDNTAG**Cys58**TSAGPHFNPLSR	1.1			1.1	1.1	1.2						
	HVGDLGNVTADKDGVADVSIEDSVISLSGDH**Cys112**IIGR							0.9	1.1				
SPINT2	SEEA**Cys179**MLR	1.1	0.9	1.2									
SSB	I**Cys18**HQIEYYFGDFNLPR							0.8					
STIM1	IDKPL**Cys56**HSEDEK	1.7		0.7									
TAGLN2	DGTVLCys63ELINALYPEGQAPVK										0.9	1.1	1.0
TGM2	**Cys10**DLELETNGR										0.8	1.1	0.9
	VVSGMVN**Cys230**NDDQGVLLGR **S230-S370** *The disulfide bond formation inactivates the calcium-dependent acyltransferase activity*										0.9	1.1	0.9
TM9SF4	TQLPYEYYSLPF**Cys64**QPSK	1.1		1.5									
TMED5	E**Cys47**FYQPMPLK	1.2		1.0									
TOMM40	TPGAATASASGAAEDGACGCLPNPGTFEE**Cys86**HRK							0.9					
TPI1	IAVAAQN**Cys67**YK	1.1	1.0	0.9									
	D**Cys87**GATWVVLGHSER	1.1	1.1	0.8									
	VPADTEVV**Cys42**APPTAYIDFAR										0.9	1.0	1.0
TRIM29	S**Cys187**LVCQASF**Cys195**ELHLKPHLEGAAFR							0.9					
TUBA1C	AV**Cys246**MLSNTTAVAEAWAR										0.9	1.0	1.0
TXNDC5	VD**Cys247**TQHYEL**Cys254**SGNQVR	1.3	1.0	1.1									
UBE2D3	IYHPNINSNGSI**Cys85**LDILR							0.9					
UQCRC1	AVELLGDIVQN**Cys154**SLEDSQIEK							0.8	0.9	0.9	0.8		
	NALVSHLDGTTPV**Cys410**EDIGR							0.9	0.9	0.9	0.9		
VAT1	**Cys50**LVLTGFGGYDK										0.7	1.0	
VCP	QAAP**Cys241**VLFFDELDSIAK							0.9	0.9	0.8	0.9	0.9	1.0
	MTNGFSGADLTEI**Cys360**QR										0.9		1.0
YWHAQ	YLAEVA**Cys134**GDDRK							0.9	0.9	0.9	0.9	1.0	1.0
YWHAZ	DI**Cys17**NDVLSLLEK										0.9	1.0	0.9

D. 1 h

D.1. LPS and LPS Mn1 effects on the proteome level, after 1-h incubation

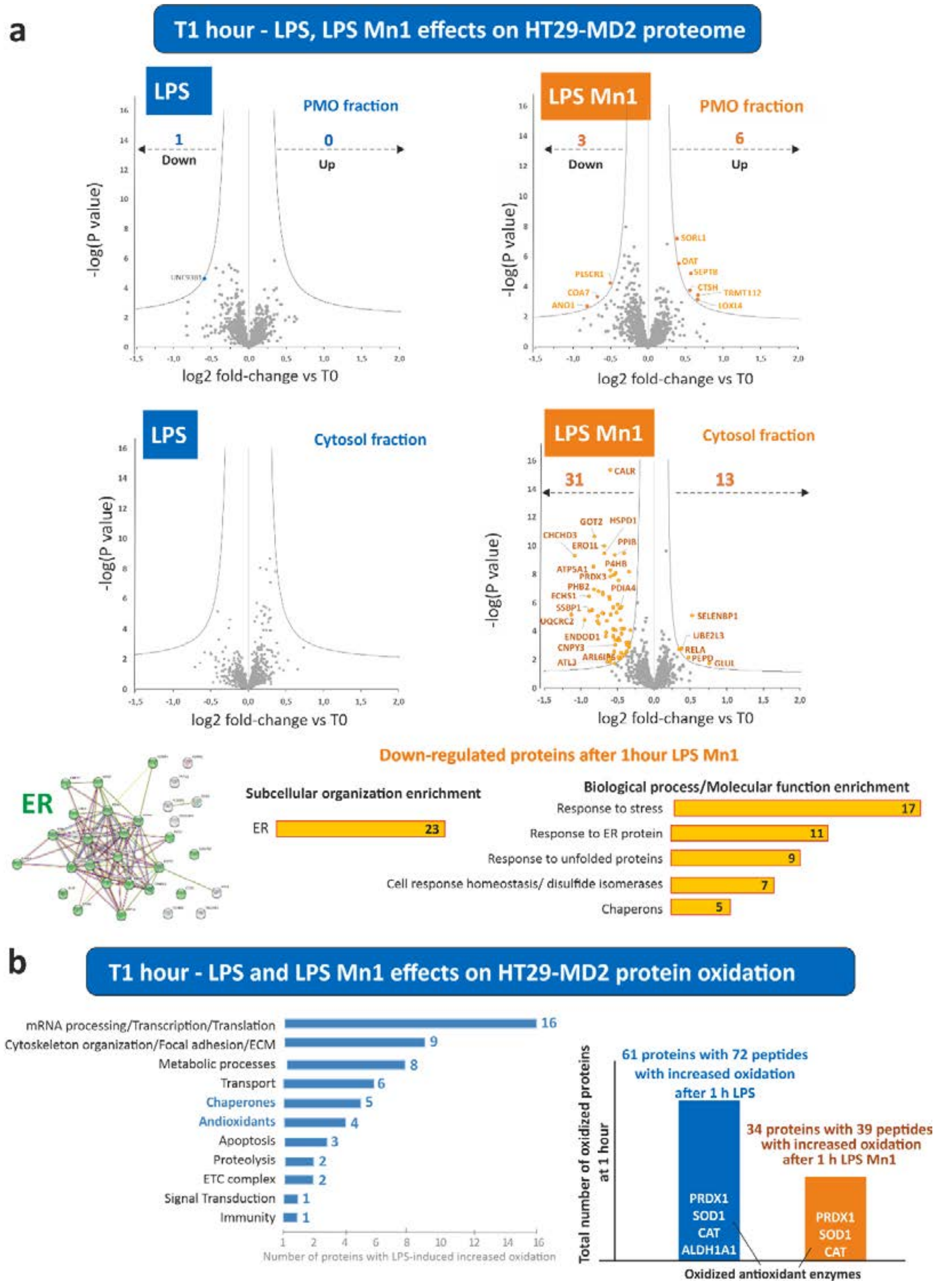

Figure S.2.7 Comparison of LPS and LPS Mn1, 1 h on protein levels and oxidation status (vs. T0 sample). a LPS and LPS **Mn1** effects were plotted in volcano plots giving the t-test p-value (in log10 scale y-axis; significant if p-value < 0.01, 1 % error) against the fold-change between two chosen conditions (in log2 scale x-axis. The LPS **Mn1** up-leveled proteins are indicated with orange circles, and the down-leveled are indicated with orange circles. In a histogramme the LPS-Mn1 down-leveled proteins are classified according to their subcellular location and biological function **b** LPS and LPS **Mn1** effects on protein oxidation status in both PMO and cytosolic fractions. Left: LPS-oxidized proteins reported according to their biological function, right: LPS-oxidized proteins in the LPS **Mn1** sample. The LPS condition is shown in blue, and the LPS **Mn1** condition is shown in orange.

Table S.2.9 Up- and down-leveled proteins after 1 hour of LPS and LPS **Mn1** stimulation of HT29-MD2 cells, vs. T0 (non-incubated or basal sample). The headline of the LPS condition is shown in blue and the **Mn1** condition is shown in orange. Down-leveled proteins are highlighted in yellow.

Protein	Protein name	Fold-change	Log2 difference	-Log (P value)
**T1 hour LPS-modified protein, in PMO**				
UNC93B1	Protein unc-93 homolog B1	0.7	-0.6	4.6
**T1 hour LPS Mn1-modified proteins, in PMO**				
TRMT112	Multifunctional methyltransferase subunit TRM112-like protein	1.6	0.7	3.4
LOXL4	Lysyl oxidase homolog 4	1.6	0.7	3.2
SEPT8	Septin-8	1.5	0.6	4.9
CTSH	Pro-cathepsin H	1.5	0.6	3.8
OAT	Ornithine aminotransferase, mitochondrial	1.3	0.4	5.5
SORL1	Sortilin-related receptor	1.3	0.4	7.2
ANO1	Anoctamin-1	0.6	-0.8	2.7
COA7	Cytochrome c oxidase assembly factor 7	0.6	-0.7	3.3
PLSCR1	Phospholipid scramblase 1	0.7	-0.5	4.2
**T1 hour LPS Mn1-modified proteins, in cytosol**				
GLUL	Glutamine synthetase	2.1	1.1	2.1
HSD17B4	Peroxisomal multifunctional enzyme type 2	1.6	0.7	3.4
BCAS1	Breast carcinoma-amplified sequence 1	1.6	0.7	1.7
NOSIP	Nitric oxide synthase-interacting protein	1.6	0.6	2.8
ACAA2	3-ketoacyl-CoA thiolase, mitochondrial	1.5	0.6	1.8
H2AFY	Core histone macro-H2A.1	1.5	0.6	2.6
HMGCS1	Hydroxymethylglutaryl-CoA synthase, cytoplasmic	1.5	0.5	2.3
FAM3C	Protein FAM3C	1.4	0.5	3.0
UBE2L3	Ubiquitin-conjugating enzyme E2 L3	1.4	0.5	3.4
GTF2I	General transcription factor II-I	1.3	0.4	7.4
TM9SF4	Transmembrane 9 superfamily member 4	1.3	0.4	5.0
SORL1	Sortilin-related receptor	1.3	0.4	4.3
HNRNPUL2	Heterogeneous nuclear ribonucleoprotein U-like protein 2	1.2	0.3	7.3
OAS3	2-5-oligoadenylate synthase 3	0.6	-0.7	2.5
KLK6	Kallikrein-6	0.6	-0.7	3.2
FKBP9	Peptidyl-prolyl cis-trans isomerase FKBP9	0.6	-0.7	2.1
IGFBP4	Insulin-like growth factor-binding protein 4	0.6	-0.7	3.1
PLSCR1	Phospholipid scramblase 1	0.6	-0.6	4.1
CALU	Calumenin	0.7	-0.6	5.7
NOMO2	Nodal modulator 2	0.7	-0.6	4.5
HYOU1	Hypoxia up-regulated protein 1	0.7	-0.6	5.2
TBL1XR1	F-box-like/WD repeat-containing protein TBL1XR1	0.7	-0.6	2.3
RCN1	Reticulocalbin-1	0.7	-0.5	2.8
MANF	Mesencephalic astrocyte-derived neurotrophic factor	0.7	-0.5	3.2
CANX	Calnexin	0.7	-0.5	2.7
ADRM1	Proteasomal ubiquitin receptor ADRM1	0.7	-0.4	3.1
PDIA6	Protein disulfide-isomerase A6	0.7	-0.4	7.6
P4HB	Protein disulfide-isomerase	0.7	-0.4	9.9
CEACAM1	Carcinoembryonic antigen-related cell adhesion molecule 1	0.7	-0.4	2.5
MYDGF	Myeloid-derived growth factor	0.7	-0.4	3.1
ERP29	Endoplasmic reticulum resident protein 29	0.7	-0.4	4.4
HSPA5	78 kDa glucose-regulated protein	0.7	-0.4	6.4
CTSC	Dipeptidyl peptidase 1	0.8	-0.4	3.8
CALR	Calreticulin	0.8	-0.4	11.1
PVRL2	Nectin-2	0.8	-0.4	4.8
FTH1	Ferritin heavy chain	0.8	-0.4	6.5
CNPY2	Protein canopy homolog 2	0.8	-0.4	4.0

PDIA4	Protein disulfide-isomerase A4	0.8	-0.4	6.3
PDIA3	Protein disulfide-isomerase A3	0.8	-0.4	4.9
STIM1	Stromal interaction molecule 1	0.8	-0.4	4.0
TXNDC5	Thioredoxin domain-containing protein 5	0.8	-0.4	6.9
ERP44	Endoplasmic reticulum resident protein 44	0.8	-0.4	5.3
ERO1L	ERO1-like protein alpha	0.8	-0.3	4.2
PPIB	Peptidyl-prolyl cis-trans isomerase B	0.8	-0.3	6.1

D.2. LPS and LPS Mn1 effects on the protein oxidation level, after 1-h incubation

Table S.2.10 Proteins with increasing oxidation level after 1-hour LPS stimulation of HT29-MD2 cells, in the PMO and cytosolic fractions, were compared to the corresponding LPS **Mn1** conditions. The oxidation status of a protein was measured by the increased *S-Ox peptides* (x/z)/T0 >1 and decreased *S-Red peptides* (y/z)/T0 <1. Cysteines are shown in bold red and proteins in bold blue are discussed in the article. The PMO fractions are highlighted in blue.

1-hour incubation									
**Protein**	**Peptides with -Cys**	**Fold-Change S-Ox in PMO**		**Fold-Change S-Ox in Cyto**		**Fold-Change S-Red in PMO**		**Fold-Change S-Red in Cyto**	
		**LPS**	**LPS Mn1**	**LPS**	**LPS Mn1**	**LPS**	**LPS Mn1**	**LPS**	**LPS Mn1**
ACTN4	**Cys352**QLEINFNTLQTK							0.6	0.7
ACTR2	VVV**Cys11**DNGTGFVK							0.9	1.0
ALDH1A1	LYSNAYLNDLAG**Cys126**IK			1.1	1.0				
ALDOA	VNP**Cys73**IGGVILFHETLYQK							0.9	1.0
ANXA1	**Cys270**ATSKPAFFAEK							0.9	1.0
ANXA2	GDLENAFLNLVQ**Cys262**IQNKPLYFA DR							0.9	
APMAP	TRDDEPV**Cys149**GRPLGIR			1.2	0.9				
**CAT**	LVNANGEAVY**Cys232**K	1.1	1.1						
CCT2	SLHDAL**Cys395**VLAQTVK							0.9	0.9
CD55	EIY**Cys225**PAPPQIDNGIIQGER	1.2	0.9						
CD59	LRENELTYY**Cys88Cys89**K			1.1	1.0				
	FEH**Cys70**NFNDVTTR			1.1	0.9				
CFL1	AVLF**Cys39**LSEDKK							0.9	1.0
CLIC1	EEFAST**Cys223**PDDEEIELAYEQVAK					0.9	1.0		
CSRP1	GLESTTLADKDGEIY**Cys167**K *Cys167 oxidation of Csrp proteins may cause structural changes and defects.*							0.9	1.0
CTSD	EG**Cys290**EAIVDTGTSLMVGPVDEV R			1.1	1.1				
	AIGAVPLIQGEYMIP**Cys329**EK							0.9	0.8
DSTN	HE**Cys135**QANGPEDLNR							0.9	0.9
ECHS1	I**Cys213**PVETLVEEAIQ**Cys225**AEK					0.9	1.0		
EEF1A1	SGDAAIVDMVPGKPM**Cys411**VESFS DYPPLGR			1.2		0.8	1.0	0.8	0.9
EEF2	**Cys369**ELLYEGPPDDEAAMGIK							0.9	0.9
EIF5A	YEDI**Cys73**PSTHNMDVPNIK							0.9	1.0
ETFB	HSMNPF**Cys42**EIAVEEAVR					0.8	0.9		
FASN	AIN**Cys1448**ATSGVVGLVN**Cys1459**L R							0.9	0.9
GDI2	TYDATTHFETT**Cys414**DDIK							0.9	0.9
GOT2	EYLPIGGLAEF**Cys106**K					0.9	0.9		

GPD2	**Cys285**VINATGPFTDSVR					0.9			
HNRNPA3	YHTINGHN**Cys196**EVK					0.9	0.9		
HNRNPK	LFQE**Cys184Cys185**PHSTDR					0.9	1.0		
HSP90AA1	VFIMDN**Cys350**EELIPEYLNFIR							0.9	0.9
	LVTSP**Cys589Cys590**IVTSTYGWTANMER							0.9	1.0
HSP90AB1	GFEVVYMTEPIDEY**Cys521**VQQLK							0.9	
	VFIMDS**Cys366**DELIPEYLNFIR							0.9	0.9
	RGFEVVYMTEPIDEY**Cys521**VQQLK							0.9	1.0
	LVSSP**Cys589Cys590**IVTSTYGWTANMER<br>Cys590 –S-Nitrocysteine							0.9	0.9
HSPA1B	FEEL**Cys306**SDLFR					0.9	0.9		
HSPA9	MEEFKDQLPADE**Cys608**NK					0.9	0.9		
HSPH1	ELNNT**Cys796**EPVVTQPKPK							0.9	1.0
	SVLDAAQIVGLN**Cys167**LR							0.9	1.0
IPO7	GIDQ**Cys757**IPLFVEAALER							0.9	0.9
LGALS3BP	STSSFP**Cys561**PAGHFNGFR							0.7	0.7
NONO	**Cys218**SEGSFLLTTFPR					0.8			
PGK1	F**Cys50**LDNGAK							0.9	1.1
PKM	NTGII**Cys49**TIGPASR							0.9	0.9
**PRDX1**	LN**Cys71**QVIGASVDSHF**Cys83**HLAWVNTPK							0.8	0.9
	HGEV**Cys173**PAGWKPGSDTIKPDVQK							0.9	1.0
PSME1	KGEDEDKGPP**Cys101**GPVN**Cys106**NEK							0.9	0.9
RACK1	TNHIGHTGYLNTVTVSPDGSL**Cys207**ASGGK							0.9	0.9
	HLYTLDGGDIINAL**Cys240**FSPNR							0.9	0.9
RANBP1	AWVWNTHADFADE**Cys42**PKPELLAIR							0.9	0.9
RPL14	**Cys54**MQLTDFILK							0.9	0.8
RPL23	ISLGLPVGAVIN**Cys28**ADNTGAK							0.9	0.9
RPL30	V**Cys92**TLAIIDPGDSDIIR					0.9	1.0		
RPL5	VGLTNYAAAY**Cys100**TGLLLAR							0.9	0.9
RPLP0	AGAIAP**Cys80**EVTVPAQNTGLGPEK					0.9	0.9		
RPS3A	N**Cys96**LTNFHGMDLTR							0.9	0.9
RPS4X	FDTGNL**Cys181**MVTGGANLGR							0.9	
RPS5	TIAE**Cys172**LADELINAAK							0.9	
RPS12	LVEAL**Cys69**AEHQINLIK							0.9	0.9
RRBP1	EL**Cys1022**HTQSSHASLR					0.9			
SDCBP	FGDQVLQINGEN**Cys166**AGWSSDK					0.9	0.9		
**SOD1**	GLTEGLHGFHVHEFGDNTAG**Cys59**TSAGPHFNPLSR			1.1	1.2				
	HVGDLGNVTADKDGVADVSIEDSVISLSGDH**Cys112**IIGR							0.8	0.9
STIP1	ALSVGNIDDALQ**Cys26**YSEAIK							0.9	0.9
TGM2	YRD**Cys554**LTESNLIK							0.9	1.0
TPI1	D**Cys87**GATWVVLGHSER	1.1	1.1						
TUBA1C	AV**Cys246**MLSNTTAVAEAWAR							0.9	1.0
UQCRC1	AVELLGDIVQN**Cys154**SLEDSQIEK					0.9	0.9		
VDAC2	PM**Cys13**IPPSYADLGK					0.9			
YWHAQ	YLAEVA**Cys134**GDDRK							0.9	1.0

YWHAZ	DICys17NDVLSLLEK					0.9	1.0	0.9	1.0
	YDDMAACys25MK							0.9	1.0

Table S.2.11 Proteins with increasing oxidation level after 15-min to 6-h LPS-stimulation of HT29-MD2 cells, in the PMO and cytosolic fractions. The oxidation status of a protein was measured by the increased *S-Ox peptides* (x/z)/T0 >1 and decreased *S-Red peptides* (y/z)/T0 <1. These ratios represent the fold change versus the T0 sample and are indicated between brackets below (detailed information is in the corresponding tables *per* time, S.2.6 (15 min), S.2.8 (30 min), S.2.10 (1 h), S.2.3. (6 h)).

Biological effects	Protein - [fold-changes] – Cys oxidized after LPS incubation			
	15 min LPS	30 min LPS	1 hour LPS	6 hours LPS
Antioxidant defense		CAT [1.1] Cys232	CAT [1.1] Cys232	CAT [1.2] Cys232<br>[1.1] Cys232
	PRDX1 [2.4] Cys173<br>[1.1] Cys173<br>[0.9] Cys71, Cys83	PRDX1 [0.9] Cys71, Cys83<br>[0.8] Cys173	PRDX1 [0.8] Cys71/Cys83<br>[0.9] Cys173	
	PRDX2 [1.2] Cys172			
	PRDX3 [1.6] Cys229	PRDX3 [1.1] Cys229		PRDX3 [1.3] Cys229
	PRDX4 [2.7] Cys51			
	PRDX6 [1.1] Cys91			PRDX6 [1.1] Cys47
	SOD1 [1.2] Cys58	SOD1 [1.1] Cys58<br>[0.9] Cys112	SOD1 [1.1] Cys58<br>[0.8] Cys112	SOD1 [1.3] Cys58<br>[0.9] Cys112
	TXNDC5 [1.1] Cys247, Cys254	TXNDC5<br>[1.3] Cys247, Cys254		TXNDC5<br>[1.1] Cys247, Cys254
		ALDH1A1 [0.6] Cys50<br>[0.9] Cys421, Cys432	ALDH1A1 [1.1] Cys126	ALDH1A1 [1.7] Cys126
		ERO1A [1.1] Cys131		
Apoptosis	PPIA [0.9] Cys62			
	TGM2 [0.9] Cys230	TGM2 [0.9] Cys230<br>[0.8] Cys10	TGM2 [0.9] Cys554	TGM2 [0.9] Cys10
	YWHAQ [0.9] Cys134	YWHAQ [0.9] Cys134	YWHAQ [0.9] Cys134	YWHAQ [0.9] Cys134
	YWHAZ [0.9] Cys17	YWHAZ [0.9] Cys17	YWHAZ [0.9] Cys17<br>[0.9] Cys25	
Chaperones	CCT2 [0.9] Cys395		CCT2 [0.9] Cys395	CCT2 [0.9] Cys395
	HSP90AB1 [0.8] Cys521<br>[0.9] Cys521<br>[0.8] Cys589-590	HSP90AB1 [0.9] Cys521<br>[0.9] Cys366	HSP90AB1 [0.9] Cys521<br>[0.9] Cys366<br>[0.9] Cys589-590	HSP90AB1 [0.9] Cys521<br>[0.9] Cys366
			HSP90AA1 [0.9] Cys350<br>[0.9] Cys589-590	HSP90AA1 [0.9] Cys529<br>[0.9] Cys350<br>[0.9] Cys589-590
	HSPH1 [0.9] Cys140	HSPH1 [0.9] Cys140<br>[0.8] Cys167<br>[0.9] Cys167	HSPH1 [0.9] Cys167<br>[0.9] Cys796	HSPH1 [0.9] Cys167<br>[0.8] Cys140<br>[0.9] Cys310<br>[0.9] Cys658
		mt HSPA9 [0.9] Cys608	mt HSPA9 [0.9] Cys608	mt HSPA9 [0.8] Cys608
				mt HSPD1 [0.8] Cys228<br>[0.9] Cy442
		PPIB [1.3] Cys202<br>[1.1] Cys202		
				STIP1 [0.9] Cys26
Proteolysis	CTSB [1.1] Cys179			
		CTSD [0.7] Cys329<br>[0.8] Cys329	CTSD [0.9] Cys329<br>[1.1] Cys290	CTSD [0.7] Cys329<br>[0.8] Cys329<br>[1.1] Cys329
	PSME1 [0.9] Cys101, Cys106		PSME1 [0.9] Cys101, Cys106	
	VCP [0.9] Cys241	VCP [0.9] Cys241<br>[0.9] Cys360		VCP [0.9] Cys241
		UBE2D3 [0.9] Cys85		UBE2D3 [0.9] Cys85
Cytoskeleton	ACTG1			ACTG1

	[0.9] Cys257, Cys272			[0.9] Cys257, Cys272
	ACTN4 [0.7] Cys352	ACTN4 [0.7] Cys352<br>[0.9] Cys794	ACTN4 [0.6] Cys352	ACTN4 [0.7] Cys352<br>[0.6] Cys352
			ACTR2 [0.9] Cys11	
	ANXA1 [0.9] Cys324	ANXA1 [0.9] Cys270	ANXA1 [0.9] Cys270	ANXA1 [0.9] Cys270
	ANXA2 [0.9] Cys163<br>[0.9] Cys262	ANXA2 [0.9] Cys262	ANXA2 [0.9] Cys262	ANXA2 [1.1] Cys133
	BSG [1.2] Cys242	BSG [1.1] Cys242		
		CAP1 [1.1] Cys375		
	CAPZB [0.9] Cys206	CAPZB [0.9] Cys206		CAPZB [0.9] Cys206
		CAPNS1 [0.8] Cys232		
		CFL1 [0.9] Cys39	CFL1 [0.9] Cys39	CFL1 [0.7] Cys39<br>[0.9] Cys39
	CSRP1 [0.9] Cys167		CSRP1 [0.9] Cys167	
	DSTN [0.9] Cys135	DSTN [0.9] Cys135	DSTN [0.9] Cys135	DSTN [0.9] Cys135
	EPCAM [3.5] Cys135	EPCAM [1.1] Cys135		
	FLNB [0.9] Cys1095			FLNB<br>[0.9] Cys178, Cys183
	ITGA6 [1.1] Cys175	ITGA6 [1.2] Cys175		
	ITGB1 [1.8] Cys691<br>[1.4] Cys691	ITGB1 [1.1] Cys691		ITGB1 [1.2] Cys691<br>[1.3] Cys691
	ITGB5 [1.1] Cys75	ITGB5 [1.1] Cys75		
		SDCBP [0.9] Cys166<br>[0.8] Cys239	SDCBP [0.9] Cys166	
	LGALS3BP [0.8] Cys561		LGALS3BP [0.7] Cys561	LGALS3BP [1.1] Cys561<br>[0.8] Cys561
	SPINT2 [1.3] Cys179	SPINT2 [1.1] Cys179		
	TUBA1B [0.9] Cys376			TUBA1B [0.9] Cys376<br>[0.9] Cys347
	MAPRE1 [0.8] Cys228			
	PLEC [0.9] Cys3008			
	KLK6 [2.5] Cys231<br>[1.4] Cys193, Cys203			
**ETC complex**		ETFB [0.8] Cys42<br>[0.9] Cys66, Cys71	ETFB [0.8] Cys42	
				SDHA [0.9] Cys475
	UQCRC1 [0.9] Cy154<br>[0.9] Cys410	UQCRC1 [0.8] Cy154<br>[0.9] Cys410	UQCRC1 [0.9] Cys154	
**Immunity**	CD46 [2.0] Cys157	CD46 [1.1] Cys157		
		CD55 [2.2] Cys81<br>[1.2] Cys225	CD55 [1.2] Cys225	CD55 [1.7] Cys81<br>[1.1] Cys225
**Metabolism**	APMAP [2.9] Cys149	APMAP [1.1] Cys149	APMAP [1.2] Cys149	APMAP [1.5] Cys149
	ECHS1 [0.9] Cys111	ECHS1 [0.9] Cys111<br>[0.9] Cys213, Cys225		
	FASN<br>[0.9] Cys1448, Cys1459	FASN<br>[0.9] Cys1118, Cys1127	FASN<br>[0.9] Cys1448, Cys1459	FASN<br>[0.8] Cys1448, Cys1459
	ALDOA [1.2] Cys178		ALDOA [0.9] Cys73	ALDOA [0.9] Cys73<br>[1.1] Cys178
	GALNT3 [1.3] Cys173	GALNT7 [1.1] Cys640		
	GLUD1 [0.9] Cys327			
				GPI [1.2] Cys404
			PGK1 [0.9] Cys50	
		DAZAP1 [0.9] Cys124		
	GOT2 [0.9] Cys106	GOT2 [1.1] Cys295	GOT2 [0.9] Cys106	GOT2 [0.9] Cys106
		GPD2 [0.9] Cys285	GPD2 [0.9] Cys285	
	ADPGK [1.1] Cys415			ADPGK [1.1] Cys415
				NPC2 [1.2] Cys93
		PKM [0.9] Cys49	PKM [0.9] Cys49	PKM [0.8] Cys49

				[0.9] Cys359
	TPI1 [1.3] Cys87<br>[1.2] Cys67<br>[0.9] Cys127<br>[0.9] Cys218	TPI1 [1.1] Cys87<br>[1.1] Cys67	TPI1 [1.1] Cys87	TPI1 [0.9] Cys127
				SHMT2 [0.8] Cys119
	PDXK [1.1] Cys112			
		HSD17B4 [0.9] Cys189		
	PPP1CA<br>[0.9] Cys155, Cys158	PPP1CA<br>[0.9] Cys155, Cys158		PPP1CA<br>[0.9] Cys155, Cys158
**mRNA processing/ Transcription/ Translation**	HSPA8 [0.9] Cys17<br>[0.9] Cys574]	HSPA8 [0.9] Cys17<br>[0.9] Cys574	HSPA8 [0.9] Cys574	HSPA8 [0.9] Cys17<br>[0.9] Cys574
	HNRNPUL1 [1.1] Cys532	HNRNPUL1 [1.1] Cys532		
	HNRNPD [0.9] Cys226	HNRNPD [0.9] Cys226		
				HNRNPA1 [0.8] Cys175
		HNRNPA3 [0.9] Cys196	HNRNPA3 [0.9] Cys196	HNRNPA3 [0.9] Cys196
	HNRNPH1 [0.9] Cys22	HNRNPH1 [0.9] Cys290		
	HNRNPL<br>[0.8] Cys260-261	HNRNPL<br>[0.9] Cys260-261		HNRNPL<br>[0.9] Cys260-261
		HNRNPF [0.9] Cys22, Cys34		HNRNPF<br>[0.9] Cys22, Cys34
			HNRNPK<br>[0.9] Cys184-185	
	DDX39B [0.9] Cys165			DDX39B [0.8] Cys165
		LUC7L2 [0.9] Cys43-44		
		NONO [0.9] Cys218	NONO [0.8] Cys218	
	EEF1A1 [0.9] Cys411		EEF1A1 [1.2] Cys411<br>[0.8] Cys411	
			EEF2 [0.9] Cys369	
			EIF5A [0.9] Cys73	
				EIF6 [0.8] Cys11, Cys15
				RPL4 [0.8] Cys208
	RPL5 [0.9] Cys100	RPL5 [0.9] Cys100	RPL5 [0.9] Cys100	RPL5 [0.9] Cys100
		RPL10A<br>[0.9] Cys66, Cys74		RPL10A<br>[0.9] Cys66, Cys74
			RPS12 [0.9] Cys69	
	RPL14 [0.9] Cys54	RPL14 [0.8] Cys54	RPL14 [0.9] Cys54	RPL14 [0.9] Cys54
		RPL23 [0.9] Cys28	RPL23 [0.9] Cys28	
	RPL30 [0.9] Cys92<br>[0.9] Cys85		RPL30 [0.9] Cys92	RPL30 [0.9] Cys85
		RPLP0 [0.9] Cys80	RPLP0 [0.9] Cys80	
	RPS3A [0.9] Cys96		RPS3A [0.9] Cys96	RPS3A [0.9] Cys96<br>[0.9] Cys139<br>[0.9] Cys201
	RPS5 [0.9] Cys172]		RPS5 [0.9] Cys172	RPS5 [0.9] Cys172
		RPS11 [0.9] Cys116		
			RPS4X [0.9] Cys181	RPS4X [0.9] Cys181
	SNRPD2 [1.1] Cys46	SNRPD3 [1.1] Cys41		
		SND1 [0.9] Cys549, Cys560		
		FUBP1 [0.9] Cys328		FUBP1 [0.9] Cys328
		SSB [0.8] Cys18		SSB [0.9] Cys18
		CNBP [0.9] Cys141, Cys151		CNBP<br>[0.9] Cys141, Cys151
**Chromatin organization**	RBBP7 [0.9] Cys166	RBBP7 [0.9] Cys166		
**DNA replication**		PCNA [0.9] Cys162		PCNA [0.9] Cys162
**Signal transduction**		GNAI2 [0.8] Cys326		GNAI2 [0.9] Cys326
	GNB1 [0.9] Cys148, Cys149	GNB1 [0.9] Cys25		GNB1 [0.9] Cys25<br>[0.9] Cys233, Cys250

		GNB2 [0.9] Cys148, Cys149		GNB2 [0.9] Cys148, Cys149
	RACK1 [0.9] Cys240	RACK1 [0.9] Cys240	RACK1 [0.9] Cys207 [0.9] Cys240	RACK1 [0.9] Cys153 [0.9] Cys240
**Transport**	CD59 [1.1] Cys88-89	CD59 [1.4] Cys88-89 [1.1] Cys70	CD59 [1.1] Cys88-89 [1.1] Cys70	CD59 [1.7] Cys88-89 [1.5] Cys70
	CLIC1 [0.9] Cys223	CLIC1 [0.9] Cys223	CLIC1 [0.9] Cys223	CLIC1 [0.6] Cys223 [0.9] Cys223
		CLTC [0.9] Cys436 [0.9] Cys824		
		COPG1 [0.8] Cys129		COPG1 [0.9] Cys129
	GDI2 [0.9] Cys414	GDI2 [0.9] Cys414	GDI2 [0.9] Cys414	
	HSPA1B [0.9] Cys17 [0.9] Cys306	HSPA1B [0.9] Cys574 [0.9] Cys306 [0.9]Cys17	HSPA1B [0.9] Cys306	
			IPO7 [0.9] Cys757	IPO7 [0.9] Cys757
	TM9SF4 [1.1] Cys64	TM9SF4 [1.1] Cys64		
		LMAN1 [1.1] Cys190		
		LMAN2 [1.1] Cys239		
	NUTF2 [1.1] Cys114	NUTF2 [1.1] Cys114		NUTF2 [1.1] Cys114
				ATP1B1 [1.3] Cys126
	SEC13 [0.9] Cys234	SEC13 [0.9] Cys234		
	STIM1 [1.6] Cys56	STIM1 [1.7] Cys56		
		TMED5 [1.2] Cys47		
		VAT1 [0.7] Cys50		VAT1 [0.9] Cys50
			RANBP1 [0.9] Cys42	
				VDAC2 [0.8] Cys13
**Unknown**	CCDC58 [1.1] Cys74			

Bibliography SI2

1. Knuefermann, P. *et al.* Myotrophin/V-1, a Protein Up-regulated in the Failing Human Heart and in Postnatal Cerebellum, Converts NFκB p50-p65 Heterodimers to p50-p50 and p65-p65 Homodimers. *J. Biol. Chem.* **277**, 23888–23897 (2002).
2. Simon, P. S. *et al.* The NF-κB p65 and p50 homodimer cooperate with IRF8 to activate iNOS transcription. *BMC Cancer* **15**, 770 (2015).
3. Misdaq, M., Ziegler, S., von Ahsen, N., Oellerich, M. & Asif, A. R. Thiopurines Induce Oxidative Stress in T-Lymphocytes: A Proteomic Approach. *Mediators of Inflammation* **2015**, 1–14 (2015).
4. Komaba, S. & Coluccio, L. M. Localization of Myosin 1b to Actin Protrusions Requires Phosphoinositide Binding. *J. Biol. Chem.* **285**, 27686–27693 (2010).
5. Hegan, P. S., Mermall, V., Tilney, L. G. & Mooseker, M. S. Roles for Drosophila melanogaster Myosin IB in Maintenance of Enterocyte Brush-Border Structure and Resistance to the Bacterial Pathogen Pseudomonas entomophila. *MBoC* **18**, 4625–4636 (2007).
6. Cheng, Y.-F. *et al.* Activation of AMP-Activated Protein Kinase by Adenine Alleviates TNF-Alpha-Induced Inflammation in Human Umbilical Vein Endothelial Cells. *PLoS ONE* **10**, e0142283 (2015).
7. Wu, T.-T. *et al.* The anti-inflammatory function of adenine occurs through AMPK activation and its downstream transcriptional regulation in THP-1 cells. *Bioscience, Biotechnology, and Biochemistry* **83**, 2220–2229 (2019).
8. Wang, G. *et al.* Activation of AMPK attenuates LPS-induced acute lung injury by upregulation of PGC1α and SOD1. *Experimental and Therapeutic Medicine* **12**, 1551–1555 (2016).
9. Lewis, M. J. *et al.* UBE2L3 Polymorphism Amplifies NF-κB Activation and Promotes Plasma Cell Development, Linking Linear Ubiquitination to Multiple Autoimmune Diseases. *The American Journal of Human Genetics* **96**, 221–234 (2015).
10. Strickson, S. *et al.* The anti-inflammatory drug BAY 11-7082 suppresses the MyD88-dependent signalling network by targeting the ubiquitin system. *Biochemical Journal* **451**, 427–437 (2013).
11. Eldridge, M. J. G., Sanchez-Garrido, J., Hoben, G. F., Goddard, P. J. & Shenoy, A. R. The Atypical Ubiquitin E2 Conjugase UBE2L3 Is an Indirect Caspase-1 Target and Controls IL-1β Secretion by Inflammasomes. *Cell Reports* **18**, 1285–1297 (2017).
12. Rahabi, M. *et al.* Divergent Roles for Macrophage C-type Lectin Receptors, Dectin-1 and Mannose Receptors, in the Intestinal Inflammatory Response. *Cell Reports* **30**, 4386-4398.e5 (2020).
13. Low, C. M. *et al.* The development of novel LTA4H modulators to selectively target LTB4 generation. *Sci Rep* **7**, 44449 (2017).
14. Matricon, J., Barnich, N. & Ardid, D. Immunopathogenesis of inflammatory bowel disease. *Self/Nonself* **1**, 299–309 (2010).
15. Farr, L. *et al.* CD74 Signaling Links Inflammation to Intestinal Epithelial Cell Regeneration and Promotes Mucosal Healing. *Cellular and Molecular Gastroenterology and Hepatology* **10**, 101–112 (2020).
16. Nishihira, J. & Mitsuyama, K. Overview of the Role of Macrophage Migration Inhibitory Factor (MIF) in Inflammatory Bowel Disease. *CPD* **15**, 2104–2109 (2009).
17. Kasama, T. *et al.* Macrophage Migration Inhibitory Factor: A Multifunctional Cytokine in Rheumatic Diseases. *Arthritis* **2010**, 1–10 (2010).
18. Ghannam, K. *et al.* Upregulation of Immunoproteasome Subunits in Myositis Indicates Active Inflammation with Involvement of Antigen Presenting Cells, CD8 T-Cells and IFNγ. *PLoS ONE* **9**, e104048 (2014).
19. Wang, J.-J. *et al.* The high expression of MTH1 and NUDT5 predict a poor survival and are associated with malignancy of esophageal squamous cell carcinoma. *PeerJ* **8**, e9195 (2020).
20. Park, S. H. *et al.* PDCD6 additively cooperates with anti-cancer drugs through activation of NF-κB pathways. *Cellular Signalling* **24**, 726–733 (2012).
21. Wang, X. *et al.* PDCD6 cooperates with C-Raf to facilitate colorectal cancer progression via Raf/MEK/ERK activation. *J Exp Clin Cancer Res* **39**, 147 (2020).
22. Sotillo, J. *et al.* Changes in protein expression after treatment with Ancylostoma caninum excretory/secretory products in a mouse model of colitis. *Sci Rep* **7**, 41883 (2017).
23. Kuroda, N. *et al.* Apoptotic Response through a High Mobility Box 1 Protein-Dependent Mechanism in LPS/GalN-Induced Mouse Liver Failure and Glycyrrhizin-Mediated Inhibition. *PLoS ONE* **9**, e92884 (2014).
24. Vassalli, G. Aldehyde Dehydrogenases: Not Just Markers, but Functional Regulators of Stem Cells. *Stem Cells International* **2019**, 1–15 (2019).
25. Ito, K., Zolfaghari, R., Hao, L. & Ross, A. Inflammation rapidly modulates the expression of ALDH1A1 (RALDH1) and vimentin in the liver and hepatic macrophages of rats in vivo. *Nutr Metab (Lond)* **11**, 54 (2014).
26. Magnusson, M. K. *et al.* Macrophage and dendritic cell subsets in IBD: ALDH+ cells are reduced in colon tissue of patients with ulcerative colitis regardless of inflammation. *Mucosal Immunol* **9**, 171–182 (2016).

27. Yasgar, A. *et al.* A High-Content Assay Enables the Automated Screening and Identification of Small Molecules with Specific ALDH1A1-Inhibitory Activity. *PLoS ONE* **12**, e0170937 (2017).
28. Xu, S.-L. *et al.* Distinct patterns of ALDH1A1 expression predict metastasis and poor outcome of colorectal carcinoma. *Int J Clin Exp Pathol* **7**, 2976–2986 (2014).
29. Vigeland, C. L. *et al.* Inhibition of glutamine metabolism accelerates resolution of acute lung injury. *Physiol Rep* **7**, e14019 (2019).
30. Maher, K. *et al.* A Role for Stefin B (Cystatin B) in Inflammation and Endotoxemia. *Journal of Biological Chemistry* **289**, 31736–31750 (2014).
31. Arlt, A. *et al.* Increased proteasome subunit protein expression and proteasome activity in colon cancer relate to an enhanced activation of nuclear factor E2-related factor 2 (Nrf2). *Oncogene* **28**, 3983–3996 (2009).
32. Mogilenko, D. A. *et al.* Metabolic and Innate Immune Cues Merge into a Specific Inflammatory Response via the UPR. *Cell* **178**, 263 (2019).
33. Mondal, N. K., Saha, H., Mukherjee, B., Tyagi, N. & Ray, M. R. Inflammation, oxidative stress, and higher expression levels of Nrf2 and NQO1 proteins in the airways of women chronically exposed to biomass fuel smoke. *Mol Cell Biochem* **447**, 63–76 (2018).
34. Nam, S. T. *et al.* Role of NADH: quinone oxidoreductase-1 in the tight junctions of colonic epithelial cells. *BMB Reports* **47**, 494–499 (2014).
35. Kosaka, T. *et al.* Involvement of NAD(P)H:Quinone Oxidoreductase 1 and Superoxide Dismutase Polymorphisms in Ulcerative Colitis. *DNA and Cell Biology* **28**, 625–631 (2009).
36. Kimura, A. *et al.* NQO1 inhibits the TLR-dependent production of selective cytokines by promoting IκB-ζ degradation. *Journal of Experimental Medicine* **215**, 2197–2209 (2018).
37. Ghosh, A. *et al.* Expression of the ARPC4 Subunit of Human Arp2/3 Severely Affects Mycobacterium tuberculosis Growth and Suppresses Immunogenic Response in Murine Macrophages. *PLoS ONE* **8**, e69949 (2013).
38. Tachibana, T., Hieda, M., Sekimoto, T. & Yoneda, Y. Exogenously injected nuclear import factor p10/NTF2 inhibits signal-mediated nuclear import and export of proteins in living cells. *FEBS Letters* **397**, 177–182 (1996).
39. Desmedt, S., Desmedt, V., Delanghe, J. R., Speeckaert, R. & Speeckaert, M. M. The intriguing role of soluble urokinase receptor in inflammatory diseases. *Critical Reviews in Clinical Laboratory Sciences* **54**, 117–133 (2017).
40. Kiyan, Y. *et al.* TLR4 Response to LPS Is Reinforced by Urokinase Receptor. *Front. Immunol.* **11**, 573550 (2020).
41. Nagase, H., Visse, R. & Murphy, G. Structure and function of matrix metalloproteinases and TIMPs. *Cardiovascular Research* **69**, 562–573 (2006).
42. Chen, G., Ge, D., Zhu, B., Shi, H. & Ma, Q. Upregulation of matrix metalloproteinase 9 (MMP9)/tissue inhibitor of metalloproteinase 1 (TIMP1) and MMP2/TIMP2 ratios may be involved in lipopolysaccharide-induced acute lung injury. *J Int Med Res* **48**, 030006052091959 (2020).
43. Moreno-Layseca, P., Icha, J., Hamidi, H. & Ivaska, J. Integrin trafficking in cells and tissues. *Nat Cell Biol* **21**, 122–132 (2019).
44. Qureshi, F. G. *et al.* Increased expression and function of integrins in enterocytes by endotoxin impairs epithelial restitution. *Gastroenterology* **128**, 1012–1022 (2005).
45. Eliceiri, B. P. Integrin and Growth Factor Receptor Crosstalk. *Circulation Research* **89**, 1104–1110 (2001).
46. Zhao, P. & Zhang, Z. TNF-α promotes colon cancer cell migration and invasion by upregulating TROP-2. *Oncol Lett* (2018) doi:10.3892/ol.2018.7735.
47. Yamaoka, T. *et al.* Transactivation of EGF receptor and ErbB2 protects intestinal epithelial cells from TNF-induced apoptosis. *Proceedings of the National Academy of Sciences* **105**, 11772–11777 (2008).
48. Feng, J. & Besner, G. E. Heparin-binding epidermal growth factor–like growth factor promotes enterocyte migration and proliferation in neonatal rats with necrotizing enterocolitis. *Journal of Pediatric Surgery* **42**, 214–220 (2007).
49. Chen, J. *et al.* Expression and Function of the Epidermal Growth Factor Receptor in Physiology and Disease. *Physiological Reviews* **96**, 1025–1069 (2016).
50. McElroy, S. J. *et al.* Transactivation of EGFR by LPS Induces COX-2 Expression in Enterocytes. *PLoS ONE* **7**, e38373 (2012).
51. Oikonomou, K. A. *et al.* Downregulation of serum epidermal growth factor in patients with inflammatory bowel disease. Is there a link with mucosal damage? *Growth Factors* **28**, 461–466 (2010).
52. Kohno, T., Konno, T. & Kojima, T. Role of Tricellular Tight Junction Protein Lipolysis-Stimulated Lipoprotein Receptor (LSR) in Cancer Cells. *IJMS* **20**, 3555 (2019).
53. Magro, F., Fraga, S., Ribeiro, T. & Soares-da-Silva, P. Intestinal Na+/K+-ATPase activity and molecular events downstream of interferon-γ receptor stimulation. *British Journal of Pharmacology* **142**, 1281–1292 (2004).
54. Gangaraju, R. *et al.* Thrombotic, inflammatory, and HIF-regulated genes and thrombosis risk in polycythemia vera and essential thrombocythemia. *Blood Advances* **4**, 1115–1130 (2020).
55. Budding, K. *et al.* Soluble CD59 is a Novel Biomarker for the Prediction of Obstructive Chronic Lung Allograft Dysfunction After Lung Transplantation. *Sci Rep* **6**, 26274 (2016).

56. Kimberley, F. C., Sivasankar, B. & Paul Morgan, B. Alternative roles for CD59. *Molecular Immunology* **44**, 73–81 (2007).
57. Iontcheva, I., Amar, S., Zawawi, K. H., Kantarci, A. & Van Dyke, T. E. Role for Moesin in Lipopolysaccharide-Stimulated Signal Transduction. *IAI* **72**, 2312–2320 (2004).
58. Chen, H. *et al.* Kallikrein 6 protease advances colon tumorigenesis *via* induction of the high mobility group A2 protein. *Oncotarget* **10**, 6062–6078 (2019).
59. Scarisbrick, I. A. *et al.* Functional Role of Kallikrein 6 in Regulating Immune Cell Survival. *PLoS ONE* **6**, e18376 (2011).
60. Cornick, S., Kumar, M., Moreau, F., Gaisano, H. & Chadee, K. VAMP8-mediated MUC2 mucin exocytosis from colonic goblet cells maintains innate intestinal homeostasis. *Nat Commun* **10**, 4306 (2019).
61. Zhou, Y. *et al.* Cellular RNA Helicase DDX1 Is Involved in Transmissible Gastroenteritis Virus nsp14-Induced Interferon-Beta Production. *Front. Immunol.* **8**, 940 (2017).
62. Hassan, M. A. *et al.* Transcriptional and Linkage Analyses Identify Loci that Mediate the Differential Macrophage Response to Inflammatory Stimuli and Infection. *PLoS Genet* **11**, e1005619 (2015).
63. Tatematsu, M. *et al.* LRRC59 Regulates Trafficking of Nucleic Acid–Sensing TLRs from the Endoplasmic Reticulum via Association with UNC93B1. *J.I.* **195**, 4933–4942 (2015).
64. Pelka, K. *et al.* The Chaperone UNC93B1 Regulates Toll-like Receptor Stability Independently of Endosomal TLR Transport. *Immunity* **48**, 911-922.e7 (2018).
65. Moore, S. M., Holt, V. V., Malpass, L. R., Hines, I. N. & Wheeler, M. D. Fatty acid-binding protein 5 limits the anti-inflammatory response in murine macrophages. *Molecular Immunology* **67**, 265–275 (2015).
66. Bertelsen, L. S., Eckmann, L. & Barrett, K. E. Prolonged interferon-γ exposure decreases ion transport, NKCC1, and Na+/K+-ATPase expression in human intestinal xenografts in vivo. *American Journal of Physiology-Gastrointestinal and Liver Physiology* **286**, G157–G165 (2004).
67. Perry, J. S. A. *et al.* Interpreting an apoptotic corpse as anti-inflammatory involves a chloride sensing pathway. *Nat Cell Biol* **21**, 1532–1543 (2019).
68. Ghishan, F. K. & Kiela, P. R. Epithelial Transport in Inflammatory Bowel Diseases: *Inflammatory Bowel Diseases* 1 (2014) doi:10.1097/MIB.0000000000000029.
69. Koumangoye, R., Bastarache, L. & Delpire, E. NKCC1: Newly Found as a Human Disease-Causing Ion Transporter. *Function* **2**, zqaa028 (2020).
70. Bellet, M. M. *et al.* HOPS/Tmub1 involvement in the NF-kB-mediated inflammatory response through the modulation of TRAF6. *Cell Death Dis* **11**, 865 (2020).
71. Mukhopadhyay, R., Jia, J., Arif, A., Ray, P. S. & Fox, P. L. The GAIT system: a gatekeeper of inflammatory gene expression. *Trends in Biochemical Sciences* **34**, 324–331 (2009).
72. Kosa, P., Szabo, R., Molinolo, A. A. & Bugge, T. H. Suppression of Tumorigenicity-14, encoding matriptase, is a critical suppressor of colitis and colitis-associated colon carcinogenesis. *Oncogene* **31**, 3679–3695 (2012).
73. Thakur, D. *et al.* Human Beta Casein Fragment (54–59) Modulates M. bovis BCG Survival and Basic Transcription Factor 3 (BTF3) Expression in THP-1 Cell Line. *PLoS ONE* **7**, e45905 (2012).
74. Hu, D.-L. *et al.* Staphylococcal enterotoxin A modulates intracellular Ca2+ signal pathway in human intestinal epithelial cells. *FEBS Letters* **579**, 4407–4412 (2005).
75. Tsai, C.-T. *et al.* TNF- down-regulates sarcoplasmic reticulum Ca2+ ATPase expression and leads to left ventricular diastolic dysfunction through binding of NF- B to promoter response element. *Cardiovascular Research* **105**, 318–329 (2015).
76. Li, T. W. H. *et al.* Effects of S -adenosylmethionine and methylthioadenosine on inflammation-induced colon cancer in mice. *Carcinogenesis* **33**, 427–435 (2012).
77. Song, Y. *et al.* SYPL1 Inhibits Apoptosis in Pancreatic Ductal Adenocarcinoma via Suppression of ROS-Induced ERK Activation. *Front. Oncol.* **10**, 1482 (2020).
78. Rotem-Yehudar, R., Galperin, E. & Horowitz, M. Association of Insulin-like Growth Factor 1 Receptor with EHD1 and SNAP29. *Journal of Biological Chemistry* **276**, 33054–33060 (2001).
79. Audrito, V., Messana, V. G. & Deaglio, S. NAMPT and NAPRT: Two Metabolic Enzymes With Key Roles in Inflammation. *Front. Oncol.* **10**, 358 (2020).
80. van Diepen, J. A., Berbée, J. F. P., Havekes, L. M. & Rensen, P. C. N. Interactions between inflammation and lipid metabolism: Relevance for efficacy of anti-inflammatory drugs in the treatment of atherosclerosis. *Atherosclerosis* **228**, 306–315 (2013).
81. Batista-Gonzalez, A., Vidal, R., Criollo, A. & Carreño, L. J. New Insights on the Role of Lipid Metabolism in the Metabolic Reprogramming of Macrophages. *Front. Immunol.* **10**, 2993 (2020).
82. Zhang, J. *et al.* Switch of NAD Salvage to de novo Biosynthesis Sustains SIRT1-RelB-Dependent Inflammatory Tolerance. *Front. Immunol.* **10**, 2358 (2019).
83. Chen, Y. *et al.* Ferritin heavy polypeptide 1 mediates apoptosis-related gene expression of duck (*Anas platyrhynchos domesticus*). *Can. J. Anim. Sci.* **96**, 619–625 (2016).
84. Fan, Y. *et al.* The effect of anti-inflammatory properties of ferritin light chain on lipopolysaccharide-induced inflammatory response in murine macrophages. *Biochimica et Biophysica Acta (BBA) - Molecular Cell Research* **1843**, 2775–2783 (2014).

85. Cano-Crespo, S. *et al.* CD98hc (SLC3A2) sustains amino acid and nucleotide availability for cell cycle progression. *Sci Rep* **9**, 14065 (2019).
86. Nguyen, H. T. T. *et al.* CD98 expression modulates intestinal homeostasis, inflammation, and colitis-associated cancer in mice. *J. Clin. Invest.* **121**, 1733–1747 (2011).
87. Weber-Boyvat, M. *et al.* OSBP-related protein 3 (ORP3) coupling with VAMP-associated protein A regulates R-Ras activity. *Experimental Cell Research* **331**, 278–291 (2015).
88. Su, X., Johansen, M., Looney, M. R., Brown, E. J. & Matthay, M. A. CD47 Deficiency Protects Mice from Lipopolysaccharide-Induced Acute Lung Injury and *Escherichia coli* Pneumonia. *J Immunol* **180**, 6947–6953 (2008).
89. Lerman, Y. V. *et al.* Sepsis lethality via exacerbated tissue infiltration and TLR-induced cytokine production by neutrophils is integrin α3β1-dependent. *Blood* **124**, 3515–3523 (2014).
90. Tufo, G. *et al.* The protein disulfide isomerases PDIA4 and PDIA6 mediate resistance to cisplatin-induced cell death in lung adenocarcinoma. *Cell Death Differ* **21**, 685–695 (2014).
91. Haapaniemi, E. M. *et al.* Combined immunodeficiency and hypoglycemia associated with mutations in hypoxia upregulated 1. *Journal of Allergy and Clinical Immunology* **139**, 1391-1393.e11 (2017).
92. Ueno, T. *et al.* Component of splicing factor SF3b plays a key role in translational control of polyribosomes on the endoplasmic reticulum. *Proc Natl Acad Sci USA* **116**, 9340–9349 (2019).
93. Kanwal, M., Smahel, M., Olsen, M., Smahelova, J. & Tachezy, R. Aspartate β-hydroxylase as a target for cancer therapy. *J Exp Clin Cancer Res* **39**, 163 (2020).
94. Anelli, T. ERp44, a novel endoplasmic reticulum folding assistant of the thioredoxin family. *The EMBO Journal* **21**, 835–844 (2002).
95. Ji, D. *et al.* Effects of inflammatory and anti-inflammatory environments on the macrophage mitochondrial function. *Sci Rep* **10**, 20324 (2020).
96. Thapa, B. & Lee, K. Metabolic influence on macrophage polarization and pathogenesis. *BMB Rep.* **52**, 360–372 (2019).
97. Jiang, Z., Chen, Z., Hu, L., Qiu, L. & Zhu, L. Calreticulin Blockade Attenuates Murine Acute Lung Injury by Inducing Polarization of M2 Subtype Macrophages. *Front. Immunol.* **11**, 11 (2020).
98. Cario, E. *et al.* Trypsin-Sensitive Modulation of Intestinal Epithelial MD-2 as Mechanism of Lipopolysaccharide Tolerance. *J Immunol* **176**, 4258–4266 (2006).
99. Annett, S., Moore, G. & Robson, T. FK506 binding proteins and inflammation related signalling pathways; basic biology, current status and future prospects for pharmacological intervention. *Pharmacology & Therapeutics* **215**, 107623 (2020).
100. Meng, Y., Murtha, A. P. & Feng, L. Progesterone, Inflammatory Cytokine (TNF-α), and Oxidative Stress (H_2O_2) Regulate Progesterone Receptor Membrane Component 1 Expression in Fetal Membrane Cells. *Reprod Sci* **23**, 1168–1178 (2016).
101. Zhao, W. *et al.* Nuclear to Cytoplasmic Translocation of Heterogeneous Nuclear Ribonucleoprotein U Enhances TLR-Induced Proinflammatory Cytokine Production by Stabilizing mRNAs in Macrophages. *J.I.* **188**, 3179–3187 (2012).
102. Leonard, A. *et al.* Selective Inactivation of Intracellular BiP/GRP78 Attenuates Endothelial Inflammation and Permeability in Acute Lung Injury. *Sci Rep* **9**, 2096 (2019).
103. Graustein, A. D. *et al.* Toll-like receptor chaperone HSP90B1 and the immune response to Mycobacteria. *PLoS ONE* **13**, e0208940 (2018).
104. Zhang, M. *et al.* A Translocation Pathway for Vesicle-Mediated Unconventional Protein Secretion. *Cell* **181**, 637-652.e15 (2020).

Supplementary Information 3

Western Blots and Gels

Supplementary Table S.3.1 Selected antibodies for proteomic data validation by Western blot quantitation

Protein	M.W kDa	Antibody Type	Target Species	Reference	Dilution	µg loaded
SOD2	25	Polyclonal Anti-Rabbit	Bovine, Human, Mouse, Rat	ThermoFisher, 200 µg, #PA1-125	1:1000	20 µg
LCN2	22	Polyclonal Anti-goat	Human, Mouse, Rat	R&D systems, 100 µL, #AF1757	1:1000	20 µg
SDCBP	32	Monoclonal Anti-mouse	Human	Abnova, 100 µL, H00006386-M01	1:10000	5 µg
ITGAV	130	Polyclonal Anti-Goat	Human	ThermoFisher, 100 µg #PA5-47096	1:10000	5 µg
TXNRD1	55	Polyclonal Anti-Rabbit	Human, Mouse, Rat	Proteintech, 150 µL, #11117-1-AP	1:10000	5 µg
Nrf2	100	Polyclonal Anti-rabbit	Human, Mouse, Rat	Proteintech, 150 µl #16396-1-AP	1:1250	5 µg
KLK6	27	Polyclonal Anti-rabbit	Human	ThermoFisher, 100 ug, #PA5-79569	1:2000	20 µg
β-Actin	45	Polyclonal Anti-rabbit	Human	Cell Signaling Technology, 100 µL, #8457	1:1000	10-20 µg
Anti-Rabbit 2ndary	-	Polyclonal Donkey, Peroxidase AffiniPure IgG (H+L)	Rabbit	Jackson Immuno Research, 500 µL, #AB_10015282	1:10000	-
Anti-Mouse 2ndary	-	Polyclonal Donkey, Peroxidase AffiniPure IgG (H+L)	Mouse	Jackson Immuno Research, 500 µL, #AB_2340770	1:10000	-

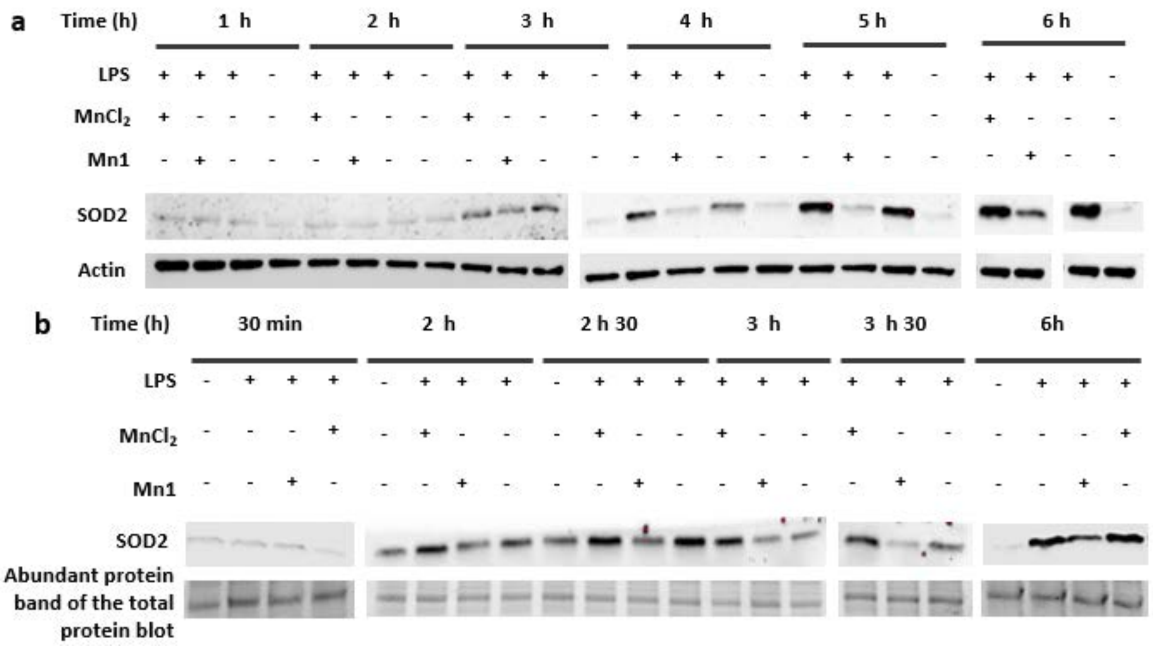

Supplementary Figure S.3.1 Kinetic study of SOD2 expression in LPS-activated HT29-MD2 cells. LPS (0.1 µg/mL)-stimulated HT29-MD2 cells were incubated, with or without **Mn1** or $MnCl_2$ (100 µM) for 30 min to 6 h. The SOD2 expression was measured by Western blot and normalized **a.** using actin as a loading control or **b.** by total protein amount using stain-free gels. For clarity, an abundant protein present in the total protein blot is shown below the SOD2 blot. To minimize errors, precast commercial acrylamide gels were used, and to exploit a kinetic experiment, care was taken to ensure that gel migrations, transfers to nitrocellulose membrane, incubation time with antibodies and revelation with ECL were carried out under the same conditions, in parallel and on the same day. Full blots for SOD2 expression are shown in Fig S.3.1A and B, and in Fig S.3.2 (bottom right panel).

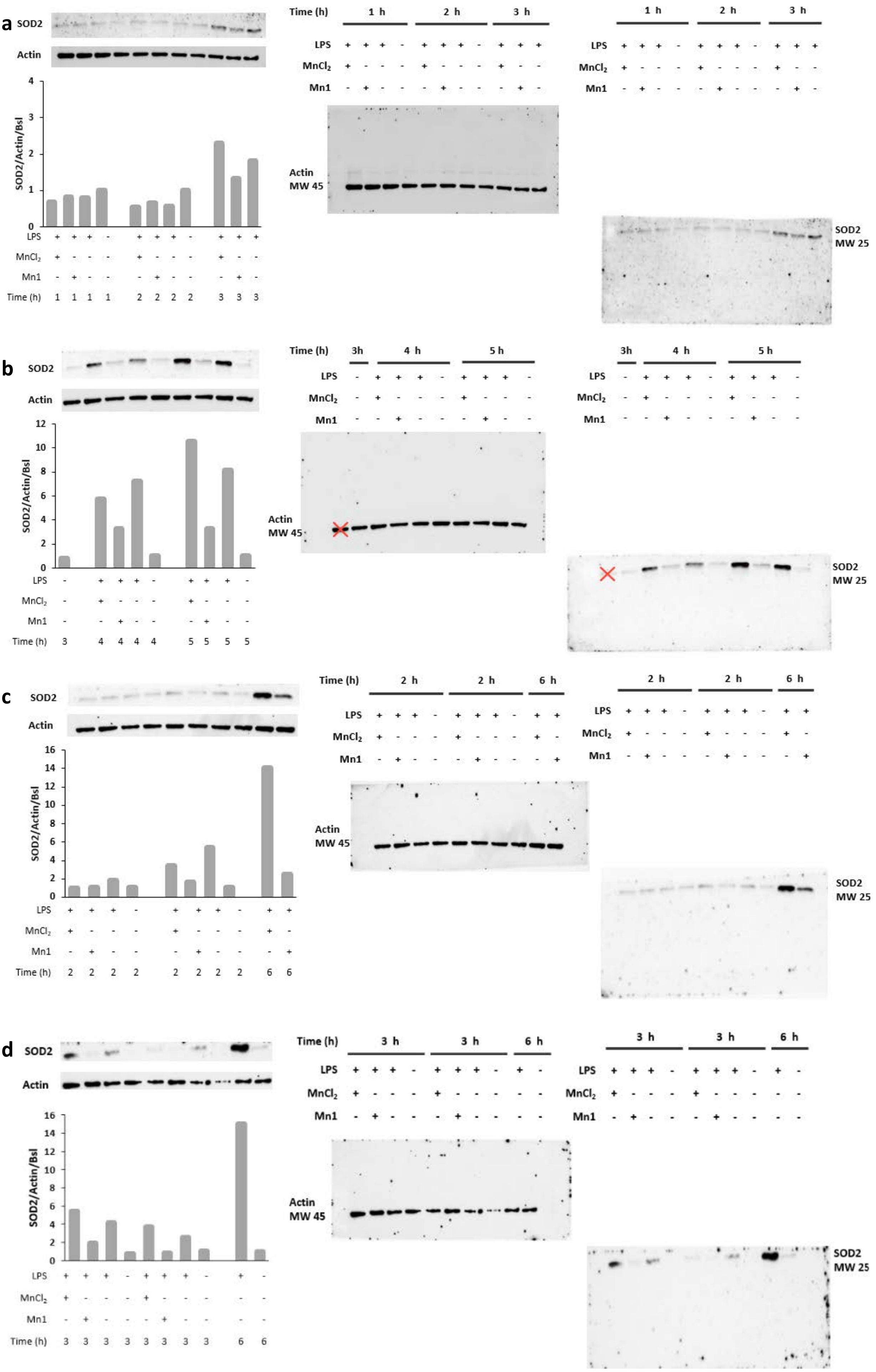
a
SOD2
Actin
SOD2/Actin/BsI
LPS
MnCl2
Mn1
Time (h)
1 h
2 h
3 h
Actin MW 45
SOD2 MW 25
b
SOD2
Actin
SOD2/Actin/BsI
LPS
MnCl2
Mn1
Time (h)
3h
4 h
5 h
Actin MW 45
SOD2 MW 25
c
SOD2
Actin
SOD2/Actin/BsI
LPS
MnCl2
Mn1
Time (h)
2 h
2 h
6 h
Actin MW 45
SOD2 MW 25
d
SOD2
Actin
SOD2/Actin/BsI
LPS
MnCl2
Mn1
Time (h)
3 h
3 h
6 h
Actin MW 45
SOD2 MW 25

Supplementary Figure S.3.1-A Kinetic study of SOD2 expression in LPS-activated HT29-MD2 cells. Full Western blots for Fig.1 after 1, 2, 3, 4, 5, 6 h. LPS (0.1 µg/mL)-stimulated HT29-MD2 cells were incubated with or without **Mn1** or $MnCl_2$ (100 µM) from 1 to 6 h.The SOD2 expression was measured by Western blot and normalized using actin as a loading control. The membrane was cut in two parts; the upper part was incubated with the actin antibody (middle panels in a, b, c, d) and the lower part with the SOD2 antibody (right panels in a, b, c, d). To minimize errors, precast commercial acrylamide gels were used, and to exploit a kinetic experiment, care was taken to ensure that gel migrations, transfers to a nitrocellulose membrane, incubation time with antibodies and revelation with ECL were carried out under the same conditions, in parallel and on the same day. Quantification is shown on the left panels in a, b, c, d.

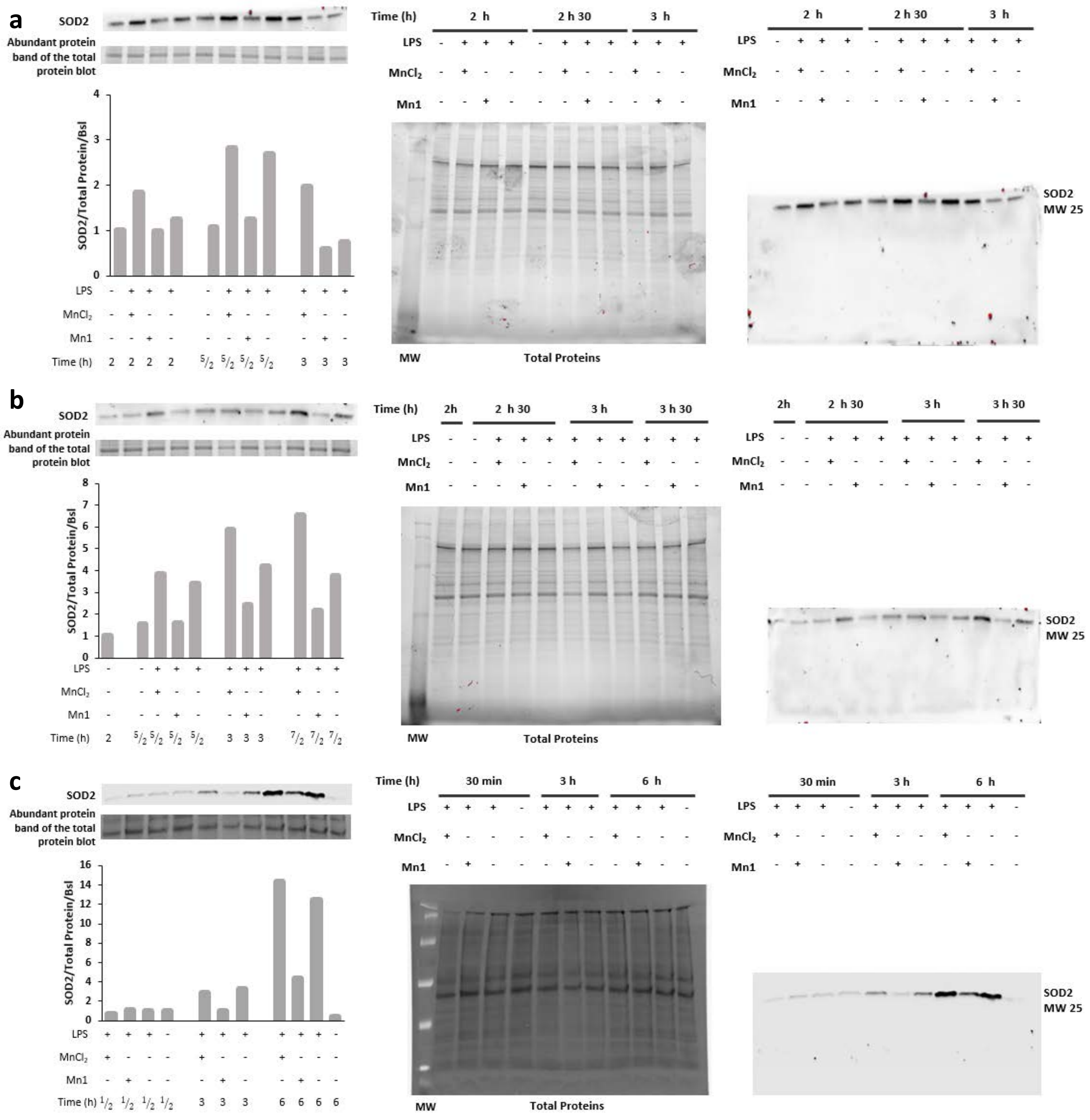

Supplementary Figure S.3.1-B Kinetic study of SOD2 expression in LPS-activated HT29-MD2 cells. Full Western blots for Fig. 1 (0.5, 2, 2.5, 3, 3.5, and 6 h). LPS (0.1 µg/mL)-stimulated HT29-MD2 cells were incubated with or without **Mn1** or $MnCl_2$ (100 µM) from 30 min to 6 h. The SOD2 expression was measured by Western blot and normalized by total protein amount using stain-free gels. **The full blots with total proteins are shown on the middle panels of a, b, c.** The membrane was cut in two parts and the lower part (shown here) was incubated with the SOD2 antibody (right panels in a, b, c). To minimize errors, precast commercial acrylamide gels were used, and to exploit a kinetic experiment, care was taken to ensure that gel migrations, transfers to nitrocellulose membrane, incubation time with antibodies and revelation with ECL were carried out under the same conditions, in parallel and on the same day. Quantification is shown on the left panels in a, b, and c. For clarity, an abundant protein present in the total protein blot is shown below the SOD2 blot.

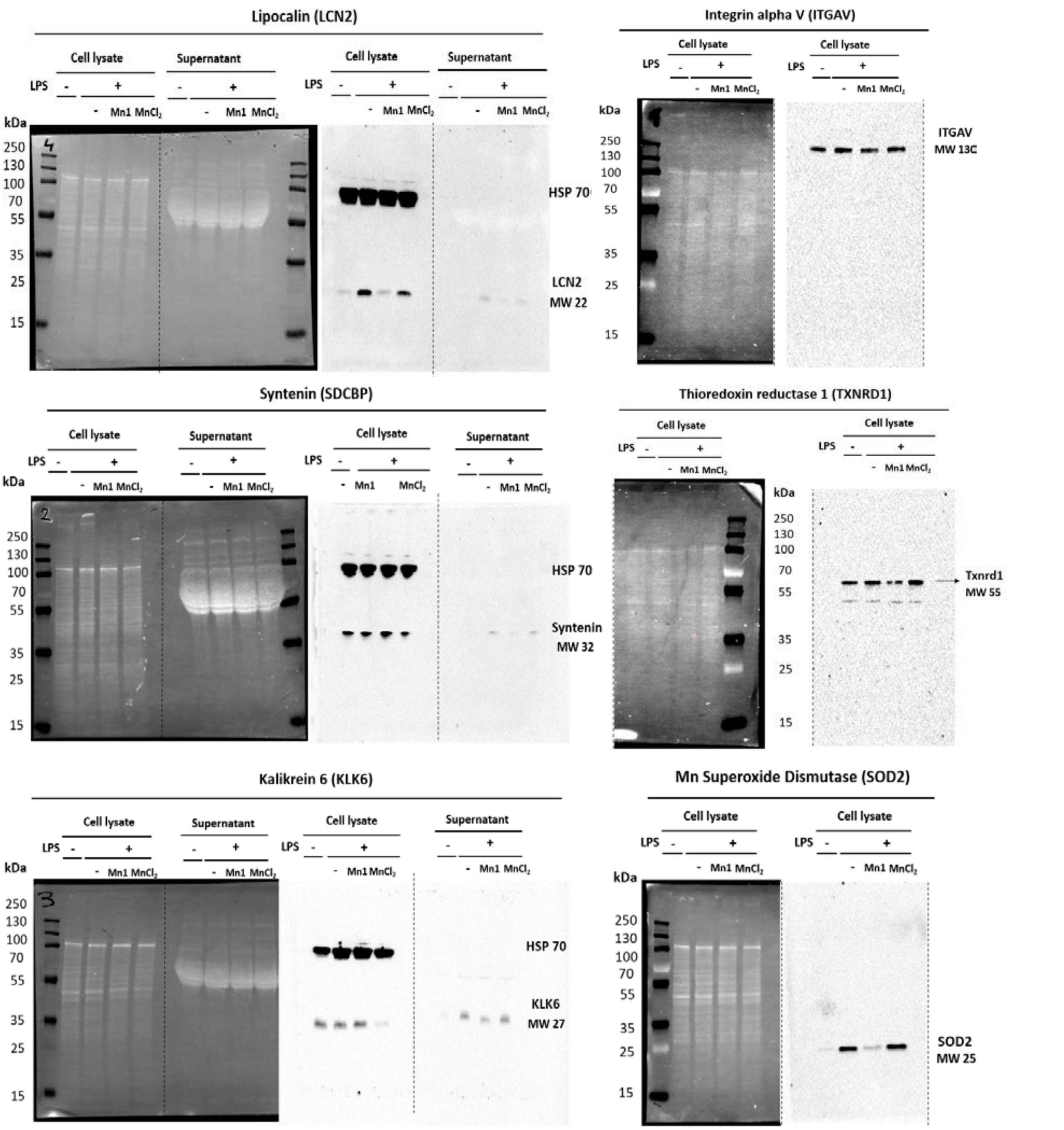

Supplementary Figure S.3.2 Full Western blots for validation of proteomic data for the selected proteins after incubation of HT29-MD2 cells for 6 h (Fig. 4). For each set of proteins, on the left is shown the full blot with total proteins and on the right is shown the revelation with the specific antibody. Proteins were quantified in cell lysates for LCN2, ITGAV, SDCBP, TXNRD1, KLK6, SOD2 and in supernatants for LCN2, SDCBP, and KLK6. HT29−MD2 cells were incubated for 6 h with just cell media, LPS (0.1 µg/mL), LPS **Mn1** (100 µM), and LPS MnCl2 (100 µM). See Table S.3.1 for antibody information. The abundance of each protein was normalized to the total amount of protein in each lane. HSP70 was used as a control for LCN2, SDCBP, and KLK6 blots. The fact that HSP70, known to be localized in the cell cytosol is only visible in the cell lysate, confirms the fact that LCN2, SDCBP, and KLK6 are also secreted and visible in the supernatant.

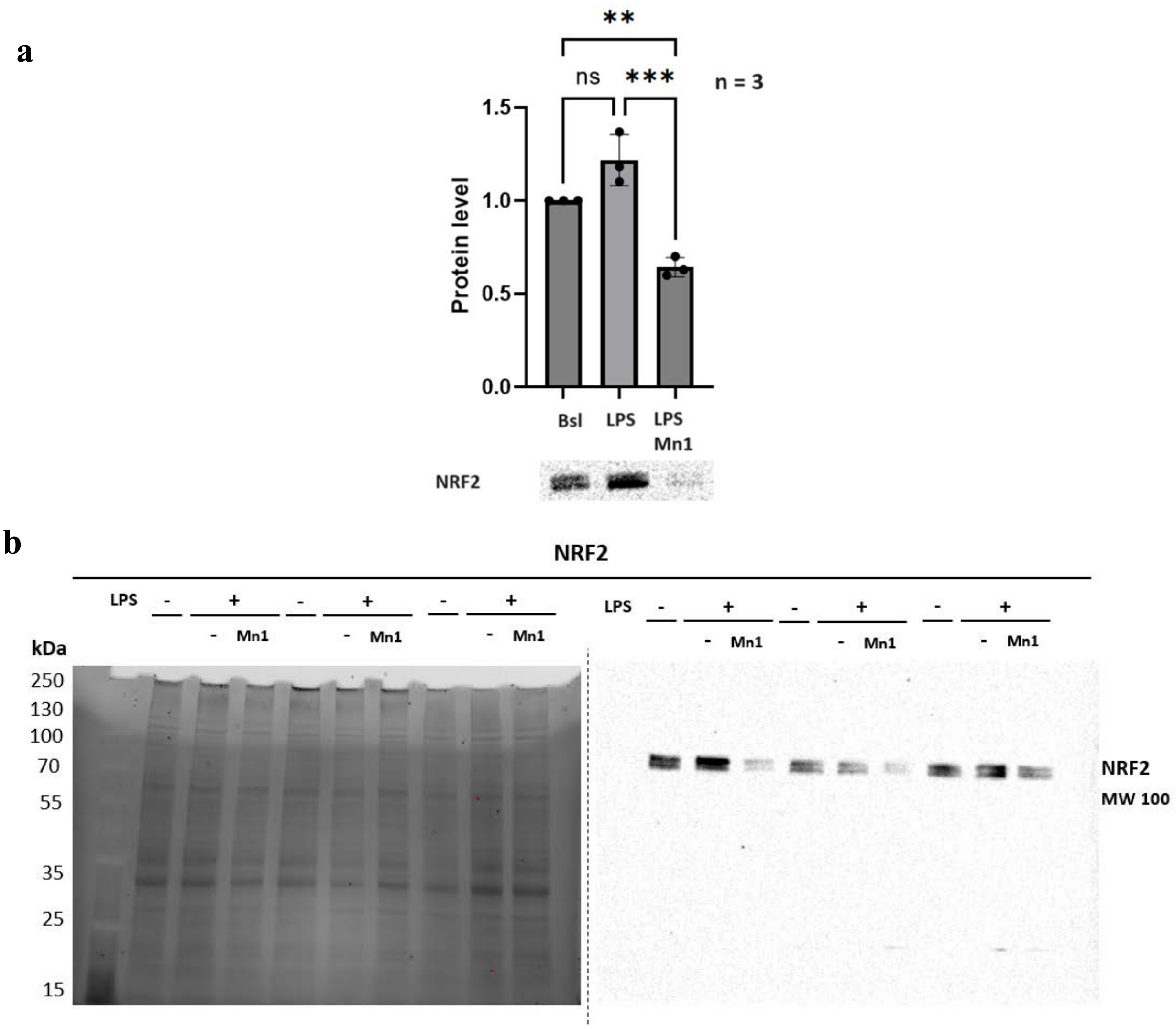

Supplementary Figure S.3.3 Western Blot quantitation of NRF2 in cell lysates. Proteins were quantified in cell lysates to deposit a similar number of proteins in each well.-HT29−MD2 cells were incubated for 6 h with just cell media (basal), LPS (0.1 µg/mL), and LPS **Mn1** (LPS 0.1 µg/mL and Mn1 100 µM). See Table S.3.1 for antibody information. **a** The protein level intensity was normalized on the basal sample, set at 1 for each independent experiment. The abundance of each protein was normalized to the total amount of protein in each lane. Data represent mean ± SEM for n independent biological experiments, indicated for each graph. The p-values were calculated using the ordinary one-way ANOVA test. The mean rank of each column was compared with the mean of every other column with (***) $p < 0.001$, (**) $p < 0.01$, and (*) $p < 0.05$. **b** Full gels used for NRF2 quantification in biologically independent triplicates (left: Stain free gel for total protein quantification, right: NRF2 revelation).

Gel imaging to evaluate total oxidation in whole HT29-MD2 cells incubated a. 15 min, b. 30 min, c. 1 h, d. 6 h, with cell media, LPS (0.1 µg/mL), LPS Mn1 (100 µM), LPS $MnCl_2$ (100 µM). Proteins were extracted and alkylated by IAM (200 mM) in denaturing buffer (1% SDS, Tris 150 mM pH 8.8, 1 % octyl β-glucopyranoside, protease inhibitors) at 37 ºC for 1 h. The maleimide functionalized fluorophore DyLight 448 was added for 1 h at 37 °C. SDS-page gel electrophoresis was performed in Tris-Glycine-SDS (1x) running buffer for 1 h at 25 mA (same amount of sample loaded in each well). Fluorescence imaging in blue light (460 nm) (left) and colorimetric imaging after 2 h Coomassie blue staining and five washes with MilliQ water (right) are shown. DyLight 448 dye excitation max at 493 nm and emission max at 518 nm.

a. 15 min

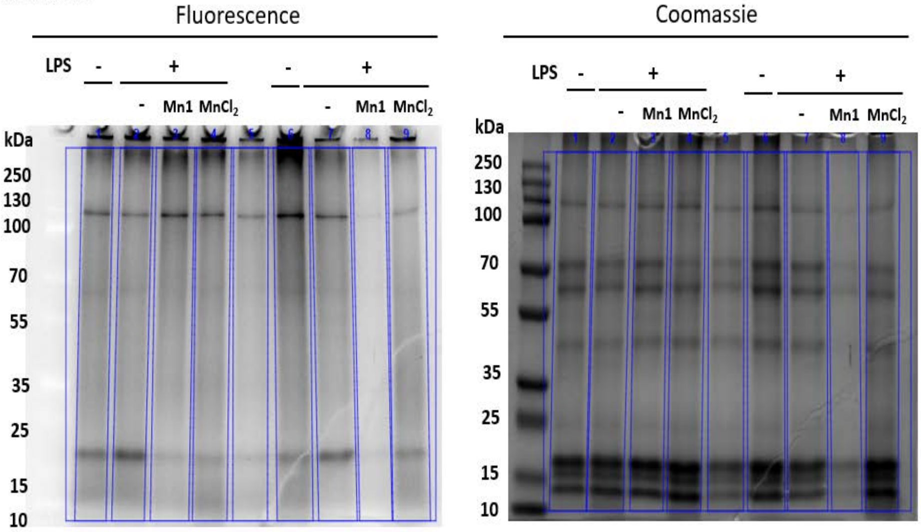

b. 30 min

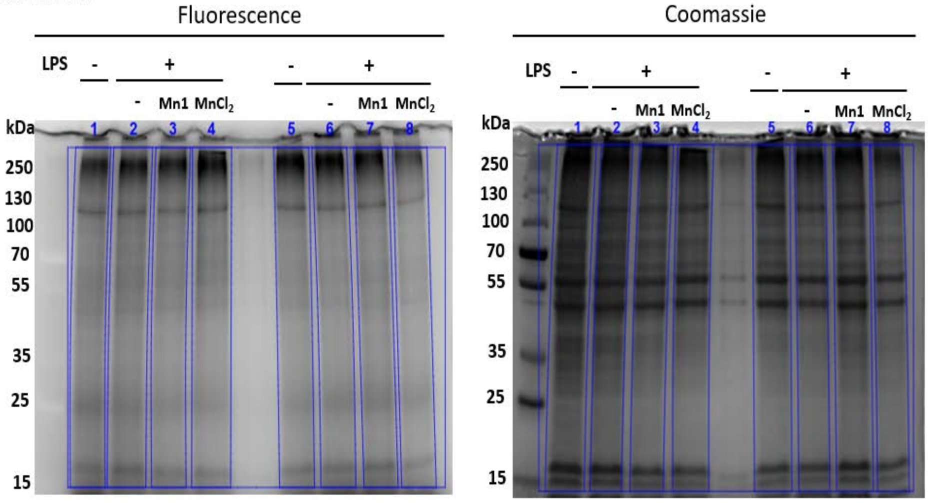

c. 1 h

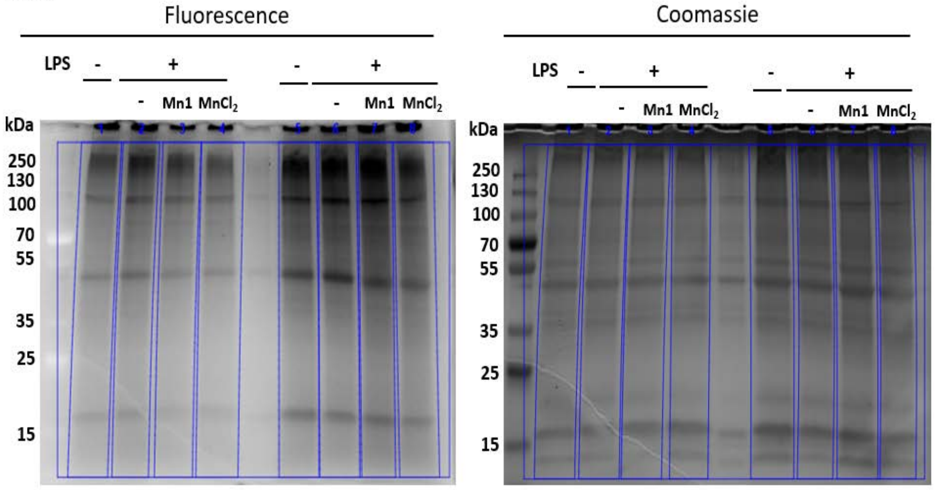

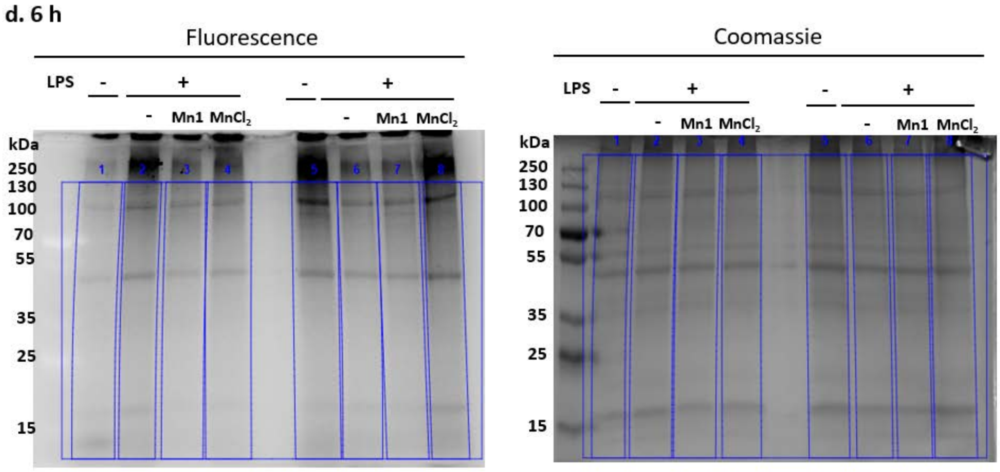

Supplementary Figure S.3.4 Gel imaging to evaluate total oxidation in whole HT29-MD2 cells incubated (Fig 5b) a. 15 min, b. 30 min, c. 1 h, d. 6 h, with cell media, LPS (0.1 µg/mL), LPS Mn1 (100 µM), LPS $MnCl_2$ (100 µM). Proteins were extracted and alkylated by IAM (200 mM) in denaturing buffer (1% SDS, Tris 150 mM pH 8.8, 1 % octyl β-glucopyranoside, protease inhibitors) at 37 ºC for 1 h. The maleimide functionalized fluorophore DyLight 448 was added for 1 h at 37 °C. SDS-page gel electrophoresis was performed in Tris-Glycine-SDS (1x) running buffer for 1 h at 25 mA (same amount of sample loaded in each well). Fluorescence imaging in blue light (460 nm) (left) and colorimetric imaging after 2 h Coomassie blue staining and five washes with MilliQ water (right) are shown. DyLight 448 dye excitation max at 493 nm and emission max at 518 nm.

Bibliography SI3

1. Chen, W. *et al.* Lipocalin-2 Exacerbates Lupus Nephritis by Promoting Th1 Cell Differentiation. *JASN* **31**, 2263–2277 (2020).
2. Du, R. *et al.* SDCBP/MDA-9/syntenin phosphorylation by AURKA promotes esophageal squamous cell carcinoma progression through the EGFR-PI3K-Akt signaling pathway. *Oncogene* **39**, 5405–5419 (2020).
3. Abdelwahab, E. M. M. *et al.* Wnt signaling regulates trans-differentiation of stem cell like type 2 alveolar epithelial cells to type 1 epithelial cells. *Respiratory Research* **20**, 204 (2019).
4. Bian, M., Wang, X., Sun, Y. & Liu, W. Synthesis and biological evaluation of gold(III) Schiff base complexes for the treatment of hepatocellular carcinoma through attenuating TrxR activity. *European Journal of Medicinal Chemistry* **193**, 112234 (2020).
5. Liao, H. *et al.* Myricetin Possesses Potential Protective Effects on Diabetic Cardiomyopathy through Inhibiting IκBα/NFκB and Enhancing Nrf2/HO-1. *Oxidative Medicine and Cellular Longevity* **2017**, e8370593 (2017).
6. Wang, P. *et al.* Macrophage achieves self-protection against oxidative stress-induced ageing through the Mst-Nrf2 axis. *Nat Commun* **10**, (2019).
7. Lizama, A. J. *et al.* Expression and bioregulation of the kallikrein-related peptidases family in the human neutrophil. *Innate Immun* **21**, 575–586 (2015).
8. Sugo, M. *et al.* Syntaxin 17 regulates the localization and function of PGAM5 in mitochondrial division and mitophagy. *The EMBO Journal* **37**, e98899 (2018).

Supplementary Information 4

UV-Vis spectroscopy. UV−visible spectra for **Mn1** (formation from enPI$_2$ ligand and anhydrous MnCl$_2$ 1.1 eq. in 4-(2-hydroxyethyl)-1-piperazineethanesulfonic acid buffer (HEPES) 50 mM at pH 7.4) titration experiments were recorded on a CARY 300 Bio UV−vis spectrophotometer with media as the reference. UV−visible spectra for all biological experiments were recorded on a SpectraMax M5 Series Multi-Mode Microplate Reader from Molecular Devices.

HT29-MD2 cell culture. HT29 human colon adenocarcinoma cells were obtained from the European Collection of Cell Cultures (Wiltshire, UK) and transfected to overexpress MD2, as previously described.[1] HT29-MD2 cells were seeded (3 x 10^6 cells/T-75 flask in 10 mL) to reach 80-90 % confluence in three days and cultured (37 °C, 5 % CO_2/air) in DMEM (Dulbecco's modified eagle medium) (Gibco) with 10% heat-activated Fetal bovine serum (FBS) (G.E. Healthcare Life Sciences), and 0.1% blasticidin (InvivoGen) at 10 µg/mL final concentration. Cell media was renewed every 2-3 days. Confluent cells were washed with Dulbecco's phosphate buffered saline (DPBS) 1X (Gibco) and detached (5-10 min, 37 °C) by trypsin-EDTA (2,2',2'',2'''-(Ethane-1,2-diyldinitrilo)tetraacetic acid also known as ethylenediaminetetraacetic acid) 0.05 % (Gibco). The reaction was quenched with fresh media. After centrifugation (3 min, 1,000 rpm), 10^6 cells (counted on Malassez lamella, 1/10 dilution) were suspended in fresh media (10 mL) and cultured. At least three cell passages were performed before experiments.

LPS cell activation. HT29-MD2 cells were seeded (200,000 cells/well in 12-well plates) to reach 80-90 % confluence after 3 days. Cells were incubated in LPS (Escherichia coli O55:B5, purified by phenol extraction, Merck) 0.1 µg/mL in 0.5 mL culture medium [labeled LPS], with/without **Mn1** (100 µM) or MnCl$_2$ (100 µM) [labeled LPS **Mn1**, LPS MnCl$_2$] at different time points (15 min to 6 h). Supernatants were collected and stored at -20 °C. Cell layers were washed once in DPBS 1X and lysed by Triton (100 µL, 1% final conc.) with protease inhibitors 1X (cOmplete™ Protease Inhibitor Cocktail, Roche). MSE Soniprep Ultrasonic disintegrator was used for sonication cell lysis. Cell lysates were harvested by scraping, sonicated, and stored at -20 °C. The compounds were previously shown to be non-toxic at these concentrations, as previously determined.[2,3,5,6]

Protein quantitation. Protein concentration in cell lysates was measured in 96-well plates by the BCA (bicinchoninic acid) method using a commercial kit (Interchim BC Assay Reagent A UP95424A, Reagent B UP95425A) according to the manufacturer's instructions, and using BSA (bovine serum albumin) and copper sulfate ($CuSO_4$) as standard (calibration curve 0-1 mg/mL).

IL-8 quantitation. Supernatants were transferred to Nunc™ Max iSorp™ 96-Well Clear Flat Bottom Transparent ELISA plates (Thermo Fisher Scientific). Quantification of IL-8 was performed using a commercial human IL-8 detection ELISA (Enzyme-linked immunosorbent assay) kit, containing IL-8-specific capture and detection antibodies, by following the instructions of the manufacturer (DuoSet #DY208). IL-8 standards and streptavidin- Horseradish peroxidase (HRP) were provided by R&D Systems. HRP-conjugated antibodies were from Rockland. TMB (3,3',5,5'-tetramethylbenzidine) substrate set was from BioLegend. Washings were performed with a HydroFlex™ microplate washer from TECAN.

Western blot. Cell lysates (20 µg/lane) were separated by Sodium dodecyl sulfate (SDS)-PAGE gels (150 V, 1 h) after protein denaturation (Laemmli buffer 1X, 95 °C, 5 min). Stain-free acrylamide gels (10 %, Bio-Rad) were activated with UV light in the ChemiDoc XRS+ System (Bio-Rad). For non-stain-free gels (10 %, Bio-Rad) actin was used for normalization. Proteins were transferred (1.3 A, 25 V, 7 min) to a nitrocellulose membrane (Bio-Rad). Blots were blocked with non-fat dry milk 5 %, (r.t., 1 h, Tween-20 0.05 % in phosphate buffered saline (PBS) 0.1 M) and then probed with primary antibodies (5 % non-fat dry milk, overnight, 5 °C). The blots were then probed (r.t., 2 h) with horseradish-peroxidase-conjugated secondary antibodies (diluted 10,000-fold, 5 % non-fat dry milk) after washings (PBS-Tween-20 0.05 %, x3), and revealed by chemiluminescence using the Clarity Western ECL substrate kit (Bio-Rad) according to the manufacturer's instructions. See Supplementary Table S.3.1 for more details on the selected antibodies.

SILAC metabolic labeling. Cell culture media, DMEM deficient in arginine and lysine (Invitrogen) was supplemented with dialyzed Fetal Bovine Serum (FBS) 10 % (deficient in arginine and lysine, Thermo Fisher Scientific), and blasticidin 0.1 % (10 µg/mL). For the heavy media, L-arginine-$^{13}C_6$ (arginine-6 or Arg6, at 86.2 µg/mL) and L-lysine-$^{13}C_6{}^{15}N_2$ (lysine-8 or Lys8, at 181.2 µg/mL), or for the light media, their light counterparts (arginine-0, at 84 µg/mL and lysine-0, at 146 µg/mL) were added (Thermo Fisher Scientific).[7] HT29-MD2 cells were seeded (2 x 10^6,

T-25 flask) and cultured (37 °C, 5 % CO_2/air) in light or heavy media. Confluent cells were washed with NaCl 0.9 % (3 mL), incubated with trypsin-EDTA 0.05 % (2 mL, 37 °C, 5-10 min), diluted in fresh media (at r.t.), and pelleted (800 rpm, 3 min). Finally, 2.5 x 10^5 to 5 x 10^5 cells were cultured in fresh media (3 mL).

SILAC cell treatment. HT29-MD2 cells were grown at least for three passages in heavy or light media prior to experiments. Heavy cells were incubated for 0 min, 15 min, 30 min, 1 h, or 6 h with heavy media to produce the Bsl conditions for the five time points. Light cells were incubated with light media supplemented with LPS (0.1 μg/mL), LPS + **Mn1** (100 μM), LPS + $MnCl_2$ (100 μM), and **Mn1** (100 μM) for 15 min, 30 min, 1 h, or 6 h. In a sample of light cells was added only light medium (0 min, no incubation). Cells were washed, trypsinated, and pelleted as described above. Cells were washed again with NaCl 0.9 % (3 mL x3). The heavy Bsl cells were added to the corresponding light cells (2 x 10^6 total, 1:1 ratio) for each time point and pelleted, creating the following samples: T0 (light cell media + heavy cell media at 0 min), and LPS (light media with LPS + heavy media), LPS **Mn1** (light media with LPS and **Mn1** + heavy media), LPS $MnCl_2$ (light media with LPS and $MnCl_2$ + heavy media), **Mn1** (light media with **Mn1** + heavy media) at different time points (15 min, 30 min, 1 h, or 6 h).

Media cytotoxicity (MTT assay). Cell growth and viability were assessed by the mitochondrial-dependent reduction of MTT (3-(4,5-dimethylthiazol-2-yl)-2,5-diphenyltetrazolium bromide) to formazan. HT29-MD2 cells were seeded (200,000 cells/well in 12-well plates) for 3 days. Cells were incubated (37 °C, 1 h) with MTT (0.5 mg/mL, Sigma Aldrich) in conventional cell culture media or depleted in lysine and arginine, complemented with light Lys-0, Arg-0, and heavy Lys-8, Arg-6. Culture media were removed, and cells were lysed in 0.04 M HCl in absolute ethanol. MTT reduction to formazan was monitored by absorbance (550 nm, 25 ºC).[4]

Incorporation rate. Cells from heavy media were extracted as described above, the pellets were washed with NaCl 0.9 % (x3) and resuspended in TCA (20 % v/v, 500 μL final v.). Cells were homogenized (2-3 min vortex, 5-10 min sonication) and centrifuged (13,000 rpm, 4 °C, 1 h, pH 1). Protein pellets were washed (x3) with ice-cold acetone (1-2 mL, 10 min, 4 ºC, 13,000 rpm). After evaporation under vacuum (5 min, SpeedVac System, Savant), protein pellets were resuspended in denaturing buffer (300 μL, Tris 150 mM pH 8.8, octyl β-D-glucopyranoside 1 %, Sigma-Aldrich, protease inhibitors 1X, complete tablets Easy pack, Roche) supplemented with dithiothreitol (DTT, 5 mM, Sigma-Aldrich). The solution was homogenized (1-2 min vortex, 1-2 min sonication), and cysteine reduction was carried out (56 °C, 30 min, stirring, pH 8). Iodoacetamide (IAM, 25 mM, Sigma-Aldrich) was added, the solution was homogenized, and alkylation of cysteines was carried out (r.t, 20 min, stirring, dark, pH 8). Trypsin (Trypsin/Lys-C Mix Mass Spec Grade, Promega) was added (3 μg) and incubated (37 °C, 18 h, pH 7). The digestion was quenched with trifluoroacetic acid (TFA, 99 % aqueous) until pH 1. Peptides were analyzed by LC/MS-MS (liquid chromatography tandem mass spectrometry) in technical triplicates, and protein identification was performed with the MaxQuant search engine (as described below). The minimal required heavy labeling efficacy is 95 %, where: $\text{Labeling efficacy (\%)} = \text{Intensities}\left[\frac{\text{H}}{(\text{H + L})}\right] \times 100$

Protein extraction from whole cells. Protein extraction was performed in acidic conditions to minimize artefactual Cys oxidation.[8] HT29-MD2 cell pellets were suspended in TCA (100 μL, 20 % v/v). Cells (kept on ice) were homogenized (2-3 min vortex, 15 min sonication), ice-cold TCA 20% (400 μL) was added (ice 10 min), and cells were centrifuged (13,000 rpm, 4 °C, 1 h, pH 1). Supernatants were discarded, and pellets were washed (x3) with ice-cold acetone (1-2 mL, 10 min, 4 °C, 13,000 rpm). After evaporation under vacuum (5 min) protein pellets were resuspended in denaturing buffer (600 μL, Urea 6 M, Tris 1.5 M pH 8.8, octyl β-glucopyranoside 1 %, protease inhibitors 1X) supplemented with IAM (200 mM). The solution was homogenized and thiol alkylation was carried out (37 °C, 1 h, stirring, dark, pH 8). The insoluble pellet was discarded, and proteins were precipitated in 600 μL ice-cold TCA 20 % (10 min), vortexed (15s), and centrifuged (13,300 rpm, 4 °C, 1 h).

Protein extraction from subcellular fractioned cells. The supernatant was removed. A subcellular protein fractionation kit for cultured cells (Thermo Fisher Scientific) containing cytosolic, plasma membrane & organelles, nuclear, chromatin-bound, and cytoskeletal extraction buffers, protease inhibitors, $CaCl_2$, and micrococcal nuclease was used following the manufacturer's protocol, except for the following modifications. IAM was added (in the dark) in all extraction buffers to a final 200 mM concentration. The pH was adjusted to 8 by the addition of Tris buffer (1.5 M, pH 8.8). The supernatant of each extracted fraction was transferred to a new tube for alkylation (37 °C, pH 7, 45 min, mixing, dark). Proteins were precipitated in ice-cold 600 μL TCA 20 % (10 min), and centrifuged (13,300 rpm, 4 °C, 1 h).

Protein Digestion. The supernatant was removed, and ice-cold acetone (1-2 mL) was added. Protein pellets were washed (x3) with ice-cold acetone (1-2 mL, 10 min, 4º C, 13,000 rpm). Trypsin was added (3 µg) and incubated (37 °C, 18 h, pH 7). The digestion was quenched with TFA (99 % aqueous) until pH 1.

Enrichment of oxidized cysteine-peptides, OcSILAC protocol in fractioned cells. After centrifugation of the TFA-acidified fractions, protein pellets were washed with ice-cold acetone (x3, 1-2 mL, 10 min, 4 ºC, 13,000 rpm). Dried protein pellets were incubated with 20 mM DTT in 600 µL denaturing buffer (37 °C, for 2 h, at pH 8). The insoluble pellet was removed, proteins were precipitated by ice-cold TCA 20% (600 µL, 13,000 rpm, 4 °C, 1 h). Protein pellets were washed similarly. Dried protein pellets were incubated with 0.34 mM biotin-HPDP (Thermo Fisher Scientific) in 700 µL denaturing buffer (37 °C, 1 h, stirring, pH 8). The insoluble pellet was removed, proteins were precipitated with ice-cold TCA, and pellets were washed similarly. After drying, proteolysis with trypsin was carried out (3 µg, 300 µL ammonium bicarbonate 50 mM, pH 7, 37 °C, 18 h). The digest was loaded on (60 µL) streptavidin resin (EZ-link, streptavidin agarose high capacity, Thermo Fisher Scientific) in Pierce centrifuge columns (0.8 mL, Thermo Fisher Scientific), and equilibrated with PBS 0.01 M (r.t, 1 h). The solution containing the non-retained fraction was recovered and acidified with 1 µL 99 % aqueous TFA. The resin was washed (x5, PBS 0.01 M 400 µL). Biotin release is obtained with DTT 20 mM (60 µL in NH_4HCO_3 50 mM) added to the resin (r.t, 2 h, stirring). The enriched fraction was eluted, the resin was washed (30 µL of NH_4HCO_3 50 mM), and the combined fractions were acidified with 1 µL 99 % aqueous TFA. Samples were stored at 4 °C for rapid use or at -20 °C until further use.

The non-retained fraction, containing peptides without cysteines (no-S) and with reduced cysteines (S-Red), and the enriched fraction, containing oxidized cysteines (S-Ox) were analyzed separately by LC-MS/MS.

LC-MS/MS. Peptide samples were desalted and concentrated on Sample Preparation Pipette Tips (ZipTip 15 µM with 0.6 µL C_{18} resin, Millipore). Reverse-phase LC was performed on a C18 column (Acclaim PepMap100, 3 µm, 75 µm i.d., 50 cm length), after desalting on a C18 pre-column (Acclaim PepMap 5 µm, 100 µm i.d. ×2 cm) with an UltiMate 3000 RSLC nano system coupled to a nano electrospray Q Exactive hybrid mass spectrometer (Thermo Fisher Scientific). Peptides were eluted at 45 ºC, at 300 nL/min, with a 180-min gradient of 1 % to 40 % of buffer B (H_2O/ACN 10:90, 0.1 % formic acid) vs. buffer A (H_2O/ACN 98:2, 0.1 % formic acid) and analyzed. Data-dependent acquisition Top10 positive mode with one survey MS scan (400–2,000 m/z, resolution 35,000) followed by MS/MS analysis of the most 10 intense precursors with a dynamic exclusion of 30 s. Each biological replicate was analyzed in three analytical replicates.

Data availability: see main text

Data analysis. Data analysis was performed using MaxQuant (version 1.5.3.8) with reviewed Swiss-Prot database (2017, March). The parameters for database searching were: multiplicity of two (Arg6, Lys8), trypsin specificity up to two missed cleavages, methionine oxidation, cysteine carbamidomethylation, asparagine and glutamine deamidation as variable modifications, initial peptide mass tolerance up to 5 ppm, fragment mass tolerance 10 ppm, decoy revert mode for identification validation and quantification on peptides Unique + Razor. Generated data were processed with an Excel macro developed to eliminate sequences that are not shared between at least two replicates.

In all cases, the quantification was performed using the light (treated) to heavy (Bsl) ratio (Light/Heavy or L/H) at the same time point T. The LT/HT ratios were compared to the L0/H0 ratios (T0 sample). Note that the L0/H0 sample was centered on 1 according to our preliminary validation (see Supplementary Information 1.A., Fig. S.1.2b, graph to the right).

Quantitative data were processed by an *in-house* R script as follows: the peptide sequences were separated into peptides without cysteines and peptides with cysteines. The group of Cys-containing peptides was processed to eliminate sequences with posterior error probability (PEP) < 0.05, seen in only one technical replicate, and separated into peptides with oxidized cysteines (S-Ox) and with reduced cysteines (S-Red). For protein expression, the protein ratios were recalculated by an in-house R script using the Light (L) and Heavy (H) ion intensities of the peptide without cysteine residues. For each protein in each biological replicate a weighted average ratio was calculated. The weighting factor accounts for the standard deviation of the peptide ratios measured in the technical replicates. To estimate the significance of up- or down-regulation, H/L ratios of LPS and H/L ratios of T0 were compared by t-test with False

Discovery Rate (FDR) correction and filtered using a statistical acceptance threshold: FDR <0.05 and "artificial within groups variance" S0=0.1. The statistic tests were performed with Perseus software[9] (1.6.10.43).

For Cys containing peptide a weighted average ratio was calculated as described above. To estimate the significance of up- or down oxidation a t-test was performed comparing the Cys containing peptide ratio to the related protein ratio. This allows to distinguish changes of Cys containing peptide level caused by redox events from those caused by changes of protein levels. The statistic tests were performed by an *in house* R script.

Functional enrichment analysis of the under-expressed and overexpressed proteins, for biological processes, molecular functions, and cellular components at each time point was performed using a combination of bioinformatic tools (DAVID, STRING, Perseus, UniProt, Gene Ontology) and further validation from literature.

Fluorescence gel imaging. Aliquots of 2 x 10^6 HT29-MD2 cells were resuspended in 600 µL TCA 20 % (pH 1). IAM alkylation and DTT reduction were performed in denaturing buffer, as described above. Dried protein pellets were incubated in 0.1 mg/mL maleimide fluorophore (DyLight Dye 488-Maleimide, Thermo Fisher Scientific) in 100 µL denaturing buffer (1 h, 37 °C, stirring, pH 7). Proteins were precipitated and protein pellets were washed as described above. Finally, to the protein pellets were added 100 µL Milli-Q water and Laemmli buffer (1X final, Bio-Rad). The samples and 5 µL of a protein molecular weight marker were loaded in SDS-PAGE (12 % Protean TGX Precast Protein Gels, Bio-Rad) in Tris-Glycine-SDS (1X) buffer (Sigma Aldrich) and electrophoresis was performed (25 mA/gel, 1 h). The gel was visualized by fluorescence imaging in blue light (Amersham Imager 680 blot and gel imager) and further stained with Coomassie blue dye (Thermo Fisher Scientific) for 2 h, rinsed with Milli-Q water (x5, 20 min) and visualized by colorimetric gel imaging.

Bibliography SI4

1. Lenoir, C. *et al.* MD-2 controls bacterial lipopolysaccharide hyporesponsiveness in human intestinal epithelial cells. *Life Sci.* **82**, 519–528 (2008).
2. Mathieu, E. *et al.* A Cell-Penetrant Manganese Superoxide Dismutase (MnSOD) Mimic Is Able To Complement MnSOD and Exerts an Antiinflammatory Effect on Cellular and Animal Models of Inflammatory Bowel Diseases. *Inorg. Chem.* **56**, 2545–2555 (2017).
3. Mathieu, E. *et al.* Intracellular location matters: rationalization of the anti-inflammatory activity of a manganese ii superoxide dismutase mimic complex. *Chem. Commun.* **56**, 7885–7888 (2020).
4. Vincent, A. *et al.* Evaluation of the compounds commonly known as superoxide dismutase and catalase mimics in cellular models. *J. Inorg. Biochem.* **219**, 111431 (2021).
5. Schanne, G. *et al.* Inertness of Superoxide Dismutase Mimics Mn(II) Complexes Based on an Open-Chain Ligand, Bioactivity, and Detection in Intestinal Epithelial Cells. *Oxid. Med. Cell. Longev.* **2022**, 1–16 (2022).
6. Zoumpoulaki, M. *et al.* Deciphering the Metal Speciation in Low-Molecular-Weight Complexes by IMS-MS: Application to the Detection of Manganese Superoxide Dismutase Mimics in Cell Lysates. *Angew. Chem. Int. Ed.* **134**, (2022).
7. Ong, S.-E. & Mann, M. A practical recipe for stable isotope labeling by amino acids in cell culture (SILAC). *Nat Protoc* **1**, 2650–2660 (2006).
8. Cao, Z., Tavender, T. J., Roszak, A. W., Cogdell, R. J. & Bulleid, N. J. Crystal Structure of Reduced and of Oxidized Peroxiredoxin IV Enzyme Reveals a Stable Oxidized Decamer and a Non-disulfide-bonded Intermediate in the Catalytic Cycle. *J. Biol. Chem.* **286**, 42257–42266 (2011).
9. Tyanova, S. & Cox, J. Perseus: A Bioinformatics Platform for Integrative Analysis of Proteomics Data in Cancer Research. in *Cancer Systems Biology* vol. 1711 133–148 (Springer New York, 2018).
